# Unfolding of RNA secondary structure impairs RNA stability to fine-tune phosphate starvation responses in rice roots

**DOI:** 10.1016/j.xplc.2025.101680

**Published:** 2025-12-17

**Authors:** Qiongli Jin, Ruiren Gao, Jiakai Yao, Zhengwei Huang, Kai Liu, Guangbo Wei, Weiguo Dong, Zhiye Wang

**Affiliations:** 1 State Key Laboratory of Plant Environmental Resilience, College of Life Sciences, Zhejiang University, Hangzhou, Zhejiang 310058, China

**Keywords:** *in vivo* RNA secondary structure, nutrient-deficient stress, RNA stability, translation, rice

## Abstract

The availability of essential macronutrients, such as inorganic phosphate (Pi) and nitrogen, limits global crop production. *In vivo* RNA secondary structure (RSS) regulates nearly all steps of the RNA life cycle and is dynamic under stress conditions. However, the roles of RSS in plant responses to nutrient deficiency remain unclear. Here, we used dimethyl sulfate mutational profiling with sequencing (DMS-MaPseq) to generate high-quality, deep-coverage *in vivo* RSS profiles of rice (*Oryza sativa*) roots in response to Pi deficiency (–P) or nitrogen deficiency (–N) stress. –P increased global RSS diversity and triggered RSS unfolding in thousands of transcripts. By comparing –P and –N RSS profiles, we identified –P-specific RSS-unfolding regions in rice roots. These regions, characterized by low GC content, were enriched within the coding sequences of many Pi starvation response transcripts. Ribosome profiling suggested that –P-specific RSS unfolding was not associated with translational regulation. In contrast, transcriptome-wide RNA decay assays under normal, –P, and Pi-refeeding conditions revealed global regulation of RNA stability in response to –P in rice roots; decreased RNA half-life was linked to RSS unfolding. Transcriptome analysis and analyses of transgenic rice plants with altered RSS demonstrated that –P-specific RSS unfolding lowers RNA stability, thus fine-tuning the accumulation of Pi starvation response transcripts and Pi homeostasis. This study systemically elucidates the dynamic roles and regulatory functions of RSS in the Pi starvation response in rice roots. Our findings underscore the importance of RSS in modulating nutrient-deficient stress responses.

## Teaser

This study systemically reveals the regulatory roles of *in vivo* RNA secondary structure (RSS) in the rice root phosphate (Pi) starvation response (PSR) and Pi homeostasis by impairing the RNA stability of PSR-related transcripts. It highlights the importance of RSS in modulating plant responses to nutrient deficiency. The transcriptome-wide *in vivo* RSS landscapes generated under Pi and nitrogen deficiency also provide a valuable resource for crop research.

## Introduction

Nutrient deficiency, particularly of phosphorus (P) and nitrogen (N), restricts global crop production (Schachtman and Shin, 2007; Bailey-Serres et al., 2019). For example, the macronutrient P is essential for plant growth and reproduction (Lambers, 2022). Plants absorb inorganic P (Pi), also termed orthophosphate, from the soil; however, due to its immobility, low solubility, or generally low abundance, limited P availability constrains crop yields on over 70% of the world’s arable land (Vance, 2001; López-Arredondo et al., 2014; Cong et al., 2020). In addition to nutrient uptake, roots sense environmental nutrient levels and respond to nutrient limitation (Gutiérrez-Alanis et al., 2018; Bouain et al., 2019). Indeed, plants have evolved numerous developmental and metabolic responses to cope with Pi limitation (Paz-Ares et al., 2022; Yang et al., 2024). The phosphate starvation response (PSR) includes remodeling of root system architecture (Péret et al., 2014; Abel, 2017), PSR signaling centered on PHR (PHOSPHATE STARVATION RESPONSE) transcription factors and SPX-domain-containing proteins (Puga et al., 2014, 2017; Wang et al., 2014; Jung et al., 2018), Pi absorption and translocation (Versaw and Garcia, 2017; Yamaji et al., 2017), and organic Pi metabolism and reuse (Paz-Ares et al., 2022). These PSR processes are regulated at multiple levels, such as transcription (Rubio et al., 2001; Zhou et al., 2008; Dai et al., 2012; Guo et al., 2015; Ruan et al., 2018), post-transcriptional gene silencing mediated by phosphate starvation–induced *miRNA399*–*OsIPS1/2* (Chiou et al., 2006; Franco-Zorrilla et al., 2007; Lin et al., 2008; Pant et al., 2008), protein phosphorylation (Chen et al., 2015b; Yang et al., 2020c), and ubiquitination (Pan et al., 2019). Investigation of additional layers of PSR regulation may provide target pathways for crop improvement, including enhanced low-Pi tolerance and improved Pi utilization.

RNA transcripts form complex secondary structures via base pairing. RNA secondary structure (RSS) in living cells is dynamic and dependent on cellular context (Xu et al., 2022). Several methods that couple small-molecule–mediated RNA modification with next- or third-generation sequencing have been developed to profile RSS *in vivo* at single-nucleotide resolution (Ding et al., 2014; Rouskin et al., 2014; Spitale et al., 2015; Weng et al., 2020; Yang et al., 2022a). Among these approaches, dimethyl sulfate (DMS) mutational profiling with sequencing (DMS-MaPseq) is widely used in studies of eukaryotic species due to its single-nucleotide resolution and high signal-to-noise ratio (Zubradt et al., 2017; Wang et al., 2019; Xu et al., 2022). In this method, DMS modifies the Watson–Crick face of unpaired adenosine (A) and cytosine (C) to *N*^1^-methyladenosine (m^1^A) and *N*^3^-methylcytidine (m^3^C) in RNA. These modifications are subsequently converted to cDNA mutations using thermostable group II intron reverse transcriptase (TGIRT) (Zubradt et al., 2017). A single RNA sequence can adopt various structures within the cell (Xu et al., 2022). Algorithms such as detection of RNA folding ensembles using expectation maximization (DREEM) (Tomezsko et al., 2020) have been developed to identify coexisting alternative RSS of the same transcripts based on DMS-MaPseq data. However, the diversity of transcriptome-wide *in vivo* RSS in plants has not yet been investigated. We recently optimized DMS-MaPseq for *in vivo* profiling of RSS in rice (*Oryza sativa*), facilitating the study of RNA structure–mediated regulation in crops (Jin et al., 2022).

RSS plays important regulatory roles in prokaryotic and eukaryotic cells (Wang et al., 2021; Zhu et al., 2021; Xu et al., 2022; Zhang and Ding, 2025). Studies of plant RNA structure have examined *in vivo* target-specific RNA structures or the transcriptome-wide RNA structurome, demonstrating that RSS regulates multiple molecular and biological processes, including miRNA biogenesis (Wang et al., 2018; Li et al., 2024; Yan et al., 2024), cleavage (Yang et al., 2020a), splicing (Ding et al., 2014; Deng et al., 2018; Liu et al., 2021), translation efficiency (Ding et al., 2014; Deng et al., 2018; Yang et al., 2020b, 2021; Xiang et al., 2023), alternative polyadenylation (Liu et al., 2021), stability (Yang et al., 2020b; Wu et al., 2024; Zhang et al., 2024), and phase separation (Zhang et al., 2019). RSS is reprogrammed and exerts regulatory effects through RNA decay or translational regulation in plants in response to environmental stresses, such as heat (Su et al., 2018) and salinity (Tack et al., 2020). RSS also participates in Pi homeostasis in rice. Rice *PHOSPHATE 1;2* (*OsPHO1;2*) encodes a Pi exporter and has an associated *cis*-natural antisense transcript known as *cis-NAT*_*PHO1;2*_ (Jabnoune et al., 2013). Reis and colleagues investigated the *in vitro* RSS of the *OsPHO1;2*–*cis-NAT*_*PHO1;2*_ interaction and proposed a model in which *cis-*NAT-mediated translational regulation of the cognate sense mRNA occurs via changes in RSS (Reis et al., 2021). Additionally, we previously identified the *in vivo* RSS of the 5′ untranslated region (UTR) of *OsPHO2* mRNA—encoding a key regulator of Pi homeostasis—using target-specific DMS-MaPseq. We discovered a target-adjacent nucleotide motif–like unfolded RNA structure downstream of the OsmiR399 target site (Jin et al., 2022). However, transcriptome-wide *in vivo* RSS profiling and the functions of RSS in response to nutrient-deficient stress remain elusive.

The root is the major plant tissue for nutrient absorption and transfer, as well as the primary tissue for sensing and responding to nutritional fluctuations in the soil (Gutiérrez-Alanis et al., 2018; Bouain et al., 2019). Here, we explored the transcriptome-wide dynamics of *in vivo* RSS and the roles of these structures in regulating responses to nutrient deficiency stress in rice roots. We obtained high-quality, deep-coverage *in vivo* RNA structurome data from rice roots in response to Pi deficiency (–P) and nitrogen deficiency (–N) using optimized DMS-MaPseq methods. Through integrated analysis of *in vivo* RNA structurome data with genome-wide ribosome profiling (Ribo-seq) and RNA half-life assays, we determined that –P-specifically induced (PSI) RSS unfolding is associated with RNA decay. Analysis of RSS-mutated transgenic rice plants further demonstrated that PSI alterations in RSS modulate RNA stability and Pi homeostasis.

## Results

### High-quality, deep-coverage *in vivo* RSS landscape of rice roots in response to Pi starvation

**Figure 1:**
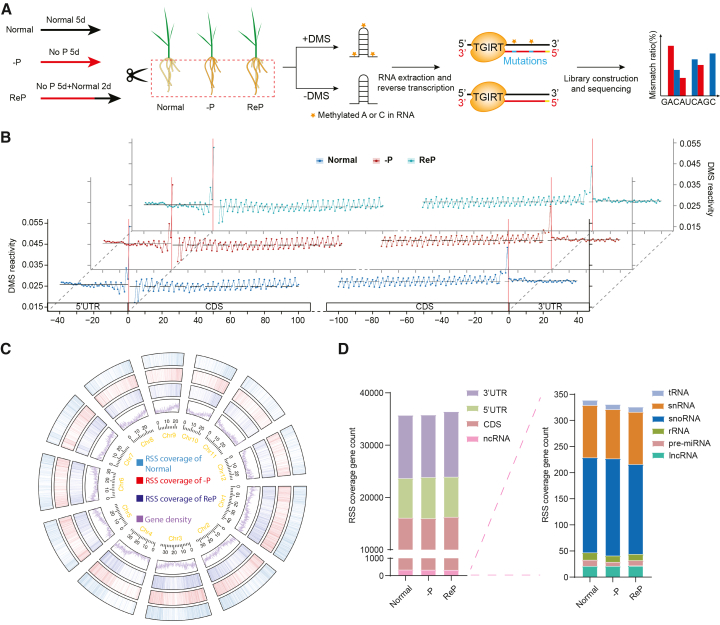
High-quality, deep-coverage *in vivo* RSS landscape in rice roots in response to Pi starvation (A) Schematic overview of transcriptome-wide *in vivo* RSS profiling using the DMS-MaPseq method. (B) Metaplots showing DMS reactivities across various mRNA regions under normal, –P, and ReP conditions. mRNAs were aligned according to start and stop codons (vertical red lines). Blue, orange, and cyan ribbons indicate standard errors from three biological replicates for normal, –P, and ReP samples, respectively. (C) Circos plot showing gene density and high-confidence RSS information coverage under normal, –P, and ReP conditions, demonstrating extensive genome-wide coverage of high-confidence RSS information. (D) Bar charts showing the distribution of high-confidence RSS regions across various RNA types, predominantly within CDS regions, 3′ UTRs, and 5′ UTRs of mRNAs. –P, Pi deficiency; ReP, Pi-refeeding following Pi deficiency; DMS, dimethyl sulfate.

To investigate RNA structural dynamics and their potential regulatory roles in the PSR of rice, we profiled the transcriptome-wide RNA structurome in roots under normal growth and Pi starvation conditions using our optimized DMS-MaPseq method (Figure 1A) (Jin et al., 2022). We sampled the roots of rice seedlings after a 5-day –P treatment and after a 5-day –P treatment followed by 2 days of Pi refeeding (hereafter, ReP), along with control samples grown under normal conditions (hereafter, normal) (Figure 1A).

For each sample, we prepared three biological replicates for DMS-treated samples (+DMS) and one reference control without DMS treatment (–DMS) for DMS-MaPseq. We obtained approximately 454–769 million clean reads for each DMS-treated replicate. More than 85% of the clean reads were uniquely mapped to the rice Nipponbare (Nip) reference genome (Ensembl Plants *Oryza sativa Japonica Group*, IRGSP 1.0) (Supplemental Table 1). Clustering analysis and principal component analysis (PCA) demonstrated high reproducibility among the three biological replicates for each sample (Supplemental Figure 1A and 1B). Changes in Pi concentration and PSR marker gene expression levels validated the effects of the –P treatments (Supplemental Figure 1C and 1D). Next, we examined the enrichment of mismatched nucleotides to assess DMS-MaPseq data quality, given that DMS methylates A and C in RNA; DMS lesions are decoded as mismatches in cDNA by the reverse transcriptase TGIRT (Zubradt et al., 2017). Compared with control samples, the mismatch percentages of A and C, but not guanosine (G) or uridine (U), were dramatically increased in all DMS-treated samples, indicating high signal-to-noise ratios (Supplemental Figure 1E).

Consistent with published *in vivo* RSS of mRNAs (Ding et al., 2014; Deng et al., 2018), we detected three-nucleotide periodicity across the coding sequence (CDS) and elevated DMS activity in the vicinity of the start codon within our RSS data, confirming high reliability (Figure 1B; Supplemental Figure 1F). Moreover, the RSS of *U1* small nuclear RNA (snRNA) predicted from DMS mutation signals was consistent with the published crystal structure (Krummel et al., 2009), further confirming the reliability of the DMS-MaPseq data (Supplemental Figure 1G). Next, we compared global RNA structural features and DMS activity among samples under normal and stress conditions. Intriguingly, –P did not alter global RNA structural features or average DMS activity across mRNAs at the genome-wide level (Figure 1B; Supplemental Figure 1H).

We then identified RNA regions with high-confidence (high-conf.) RSS information at the transcriptome-wide level. We divided each transcript into 100-nt windows (see methods). Windows harboring average mismatch counts of >20 for both A and C combined were regarded as high-conf. RSS information regions (see methods). Each DMS-treated biological replicate produced approximately 100,000 high-conf. RSS information windows associated with approximately 10,000 genes, representing ∼44% of the expressed genes (Supplemental Figure 2A). Because RSS information was highly reproducible among biological replicates (Supplemental Figure 1A, 1B, and 2B), we merged the three biological replicates to increase coverage. Notably, each merged sample contained approximately 190,000 high-conf. RSS information windows associated with ∼16,000 genes, constituting at least 60% of the expressed genes (Figure 1C; Supplemental Figure 2A). On average, each covered gene contained 11 high-conf. 100-nt RSS information windows (Supplemental Figure 2A). Regions with higher gene density encompassed more high-conf. RSS windows, confirming the reliability of the RSS data (Figure 1C). Additionally, high-conf. RSS information covered various RNA types, including mRNAs, non-coding RNAs, pre-miRNAs, ribosomal RNAs, transfer RNAs, snRNAs, and small nucleolar RNAs (Figure 1D). The majority of high-conf. RSS windows (99%) corresponded to mRNAs: 44% covered CDS regions, 34% covered 3′ UTRs, and 21% covered 5′ UTRs (Figure 1D). Notably, low coverage of non-coding RNAs was observed, which may be attributed to low expression levels or incomplete genome annotation (Figure 1D). These results indicate that the rice root RNA structurome data are highly reliable and provide deep coverage.

### –P increases RSS diversity in rice roots

Although –P did not alter ensemble DMS activity (Supplemental Figure 1H), we investigated whether –P stress induces changes in RSS. A single RNA sequence can adopt various structures within the cell (Xu et al., 2022). We first assessed whether –P stress affects *in vivo* RSS diversity using the RSS clustering algorithm DREEM (Tomezsko et al., 2020), which identifies alternative RNA conformations of the same RNA sequence based on DMS-MaPseq data (Tomezsko et al., 2020). Because DREEM analysis requires super-high sequencing depth (at least 50,000 reads per 100-nt window), and given the high quality and reproducibility of the DMS-MaPseq data among the three biological replicates (Supplemental Figures 1A, 1B, and 2B), we merged the three biological replicates for this analysis. Additionally, we divided each transcript into 100-nt windows and retained those with at least 50,000-read coverage. Approximately 5,575–7,354 100-nt windows (corresponding to 504–642 genes) met this threshold in each merged sample. We selected 4,110 windows common to all three growth conditions for DREEM analysis.

**Figure 2:**
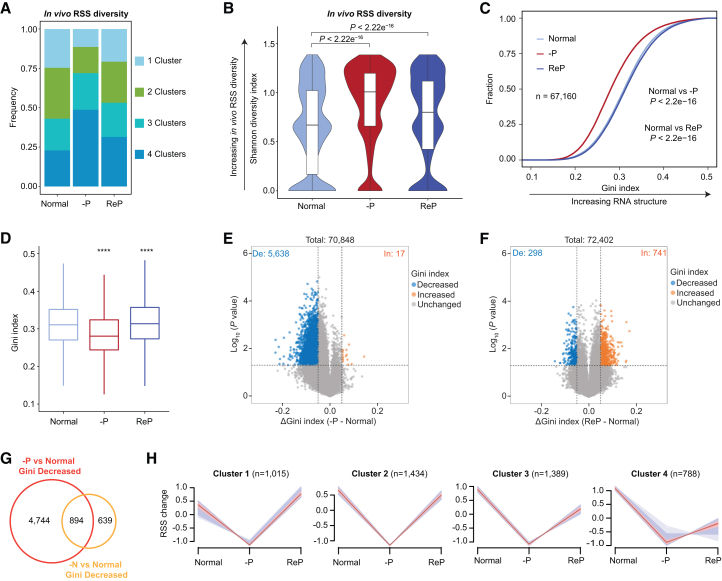
Pi starvation induces global RSS diversity and RSS unfolding (A) DREEM analysis showing increased RNA structural heterogeneity *in vivo* and elevated RSS diversity under –P compared with normal and ReP conditions. The DREEM algorithm groups sequencing reads derived from each structure into distinct clusters, each representing a unique RNA secondary structure. The number of clusters reflects the total number of alternative RNA structures within a 100-nt RNA window *in vivo*. (B) Violin plots showing increased Shannon diversity indices under –P compared with normal and ReP conditions. The *p* values were calculated using the Wilcoxon test. (C and D) Cumulative distribution curves (C) and boxplots (D) showing global decreases in Gini index under –P compared with normal and ReP conditions. *p* values were calculated using the Kolmogorov–Smirnov test in (C) and the Wilcoxon test in (D). ∗∗∗∗, *p* < 0.0001. (E and F) Volcano plots showing altered Gini indices of high-conf. RSS windows under –P (E) and ReP (F) compared with normal conditions. De, RSS windows with significantly decreased Gini indices; In, RSS windows with significantly increased Gini indices. Numbers of altered RSS windows are shown. (G) Venn diagram showing overlap between –P- and –N-induced RSS windows with decreased Gini indices. (H) Identification of clusters of windows exhibiting –P-induced decreases in Gini index using the R package Mfuzz. In (B) and (D), midlines and box edges indicate medians and quartiles, respectively. Whiskers extend to the farthest data point within 1.5 times the interquartile range (IQR) from box edges. –P, Pi deficiency; ReP, Pi-refeeding following Pi deficiency; –N, nitrogen deficiency.

DREEM showed that more than 76% of the tested windows could be clustered into two to four alternative RNA structures, suggesting widespread RSS heterogeneity *in vivo* (Figure 2A; Supplemental Table 2). Remarkably, –P treatment increased the ratio of windows with four alternative RNA structures but decreased the ratio of windows with one or two alternative RNA structures compared with samples under normal and ReP conditions (Figure 2A). We obtained similar results when analyzing windows with at least 25,000-read coverage: 11,564 100-nt windows (937 genes) (Supplemental Figure 3A). These findings indicate that –P increases RSS heterogeneity in rice roots.

To quantify RSS diversity, we utilized the Shannon diversity index to simultaneously measure the number and ratio of alternative RSS (Finkel et al., 2019) (see methods); a higher Shannon diversity index indicated greater RSS diversity. Consistent with the RSS clustering results (Figure 2A; Supplemental Figure 3A), –P increased the global Shannon diversity index, whereas ReP partially restored this index toward levels observed under normal conditions (Figure 2B; Supplemental Table 2). This pattern was also observed when analyzing windows with at least 25,000 read coverage across all three growth conditions (Supplemental Figure 3B). Our results constitute further evidence that –P enhances *in vivo* RSS diversity in rice roots.

In summary, DREEM analysis demonstrated that –P stress induces increased RSS diversity in rice roots, suggesting the occurrence of global RSS reprogramming in response to –P stress.

### –P stress triggers transcriptome-wide RSS unfolding in rice roots

To examine changes in RSS in response to Pi starvation in greater detail, we used the Gini index to indicate RNA conformation: a high Gini index reflects a highly folded RSS, whereas a low Gini index denotes unfolded RSS (Zubradt et al., 2017; Zhang et al., 2024). Gini indices of high-conf. RSS information windows were highly reproducible among the three biological replicates (Supplemental Figure 2B). Compared with normal growth conditions, the global Gini index was significantly decreased under –P but was restored to normal levels under ReP (Figures 2C and 2D). These results reveal transcriptome-wide RSS unfolding and increased RNA accessibility in response to –P.

We then identified RSS regions that were significantly altered by –P treatment. Consistent with the transcriptome-wide analysis, 5,638 windows (corresponding to 3,123 genes) showed significant decreases in Gini index upon –P treatment compared with normal conditions (*p* < 0.05, ΔGini index [–P – normal] ≤ −0.05); only 17 windows (corresponding to 16 genes) showed significant increases (*p* < 0.05, ΔGini index [–P – normal] ≥ 0.05) (Figure 2E; Supplemental Table 3). In contrast, relative to normal conditions, the Gini indices of only 298 windows (corresponding to 255 genes) significantly decreased (*p* < 0.05, ΔGini index [ReP – normal] ≤ −0.05), whereas those of 741 windows (corresponding to 639 genes) significantly increased (*p* < 0.05, ΔGini index [ReP – normal] ≥ 0.05) under ReP conditions (Figure 2F; Supplemental Table 3). These results indicate that –P triggers global RSS unfolding in rice roots.

Our results demonstrate that –P induces global changes in RSS, predominantly unfolding, suggesting that RSS plays important roles in regulating the PSR in rice roots.

### Identification of –P-specific RSS-unfolding transcripts

To further identify –P-specific RSS-unfolding regions, we profiled transcriptome-wide RSS in Nip plants subjected to 5 days of treatment with low levels of nitrogen (–N), another macronutrient essential for plant growth (Liu et al., 2022b). We used three DMS-treated (+DMS) biological replicates and one DMS-untreated (–DMS) control, yielding high-quality, deep-coverage RNA structurome data under –N conditions (Supplemental Figures 1 and 4A–4D; Supplemental note). Similar to –P stress, –N stress predominantly triggered RSS unfolding in a large number of transcripts (Supplemental Figure 4E and 4F). The Gini indices of 1,533 windows (corresponding to 1,136 genes) significantly decreased (*p* < 0.05, ΔGini index [–N – normal] ≤ −0.05), whereas those of only 158 windows (corresponding to 102 genes) significantly increased (*p* < 0.05, ΔGini index [–N – normal] ≥ 0.05) under –N relative to normal conditions (Supplemental Figure 4G; Supplemental Table 3). These –N-induced RSS-unfolding transcripts were enriched in Gene Ontology (GO) terms related to responses to nutrient levels, protein deubiquitination, protein transport, glyceraldehyde-3-phosphate metabolism, sphingolipid metabolism, and transcription (Supplemental Figure 4H). In contrast, –N-induced RSS-folding transcripts were associated with carbohydrate metabolism and trehalose metabolism during stress responses (Supplemental Figure 4I). Notably, transcripts of several genes involved in N sensing and signaling exhibited unfolding in response to –N stress, including the nitrate signaling gene NIN-like protein 3 (*OsNLP3*) (Liu et al., 2022a; Zhang et al., 2022) and the nitrate transporter gene NITRATE TRANSPORTER *1.1* (*OsNRT1.1*) (Fan et al., 2017), indicating a potential regulatory role for RSS in –N responses (Supplemental Figure 4J and 4K).

Next, we compared –P- and –N-induced RSS-unfolding windows. More than half of the –N-induced RSS-unfolding windows (58.3%) were also induced by –P (15.8%) (Figure 2G). We subtracted these overlapping windows from the –P-induced RSS-unfolding set to obtain candidate RSS-unfolding windows specifically induced by –P. We applied the Mfuzz algorithm (fuzzy c-means), based on Euclidean distance, to the Gini index of each window across normal, –P, and ReP conditions to cluster these candidate –P-specific RSS-unfolding windows into four groups (Figure 2H). RSS windows in clusters 1 to 3 were unfolded under –P but exhibited approximately normal folding after ReP (Figure 2H). We defined these RSS windows as putative PSI RSS-unfolding RNA regions, comprising 3,838 RSS windows that correspond to 2,437 genes (Supplemental Table 3).

### Features of PSI RSS-unfolding RNA regions

**Figure 3:**
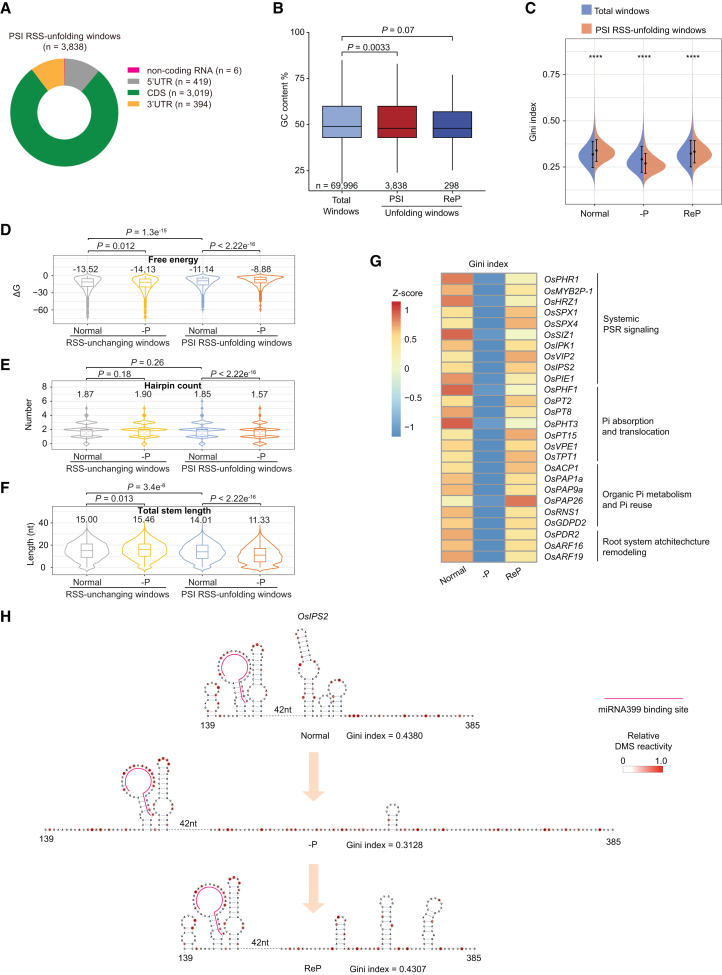
Characteristics of PSI RSS-unfolding regions (A) Percentages of PSI RSS-unfolding windows at various transcript locations. PSI RSS-unfolding windows were predominantly located in CDS regions, followed by 5′ UTRs, 3′ UTRs, and non-coding RNAs. Numbers of the indicated transcripts are shown. (B) Boxplots showing significantly lower GC content in PSI RSS-unfolding windows than in total RSS windows and unfolding RSS windows under ReP conditions. (C) Violin plots showing that the global Gini index of PSI RSS-unfolding windows was higher than that of total RSS windows under normal and ReP conditions but lower under –P conditions. *p* values were calculated using the Wilcoxon test. ∗∗∗∗, *p* < 0.0001. (D–F) Plots showing free energy (D), hairpin count (E), and total stem length (F) of RSS-unchanging windows (*n* = 3543) and PSI RSS-unfolding windows (*n* = 3838) under normal and –P conditions. Corresponding average values are shown. ΔG, Gibbs free energy change. (G) Heat map showing changes in Gini indices of PSI RSS-unfolding transcripts with known involvement in the PSR and Pi homeostasis. PSR, Pi starvation response. (H) RSS modeling of *OsIPS2* based on DMS-MaPseq data, showing unfolded RNA structure under –P compared with normal and ReP conditions. In (B and D–F), *p* values were calculated using the Wilcoxon test. Midlines and box edges indicate medians and quartiles, respectively. Whiskers extend to the farthest data point within 1.5 times the IQR from box edges. –P, Pi deficiency; ReP, Pi-refeeding following Pi deficiency; PSI, Pi starvation induced; RSS, RNA secondary structure.

We characterized the sequence and structural features of the PSI RSS-unfolding RNA regions. Most transcripts in these regions originated from protein-coding genes, and PSI RSS-altered regions were predominantly located within CDSs (79% of the total) (Figure 3A). Only six RSS windows were located in non-coding RNAs, including *OsIPS2* (Hou et al., 2005) (Figure 3A). Because RNA regions with low GC content are generally more flexible due to the lower stability of A-U base pairs relative to G-C base pairs (Chan et al., 2009), we assessed GC content in PSI RSS-unfolding regions. Indeed, we found that PSI RSS-unfolding windows, but not ReP-mediated RSS-unfolding windows, exhibited significantly lower GC content compared with genome-wide RSS windows (Figure 3B). These results indicate that PSI RSS-unfolding regions with low GC content form flexible RNA structures.

We subsequently compared the RNA structures of total and PSI RSS-unfolding windows under normal, –P, and ReP conditions. As expected, PSI RSS-unfolding windows showed significantly decreased RSS folding, as indicated by a lower Gini index, than total RSS windows under –P stress (Figure 3C). In contrast, under normal and ReP conditions, PSI RSS-unfolding windows displayed significantly higher RSS folding than total RSS windows (Figure 3C). These findings confirm pronounced changes in RSS within PSI RSS-unfolding RNA regions between normal and –P conditions, revealing the structural flexibility of these regions. Intriguingly, this feature and lower GC content were observed in both the –P/–N common and –N-unique RSS-unfolding regions, highlighting shared characteristics among RSS-unfolding regions induced by –P and –N (Supplemental Figures 5A–5C; Supplemental note).

Next, we assessed putative RSS features responsive to –P. We selected the top 5% of RSS windows with the lowest ΔGini index (|–P –Normal|) values to serve as control RSS-unchanging windows (*n* = 3543). We integrated our DMS-MaPseq data into the RSS feature analysis (see methods). The results showed that, in PSI RSS-unfolding windows, free energy and unpaired ratio values increased; hairpin count, internal loop count, and total and average stem lengths decreased from normal to –P conditions (Figures 3D–3F; Supplemental Figures 6A–6C). In contrast, these RSS indices in RSS-unchanging windows were comparable between the two conditions (Figures 3D–3F; Supplemental Figures 6A–6C), validating the fidelity of the RSS analysis. To further evaluate RSS features of PSI RSS-unfolding regions, we compared RSS indices of PSI RSS-unfolding windows with those of RSS-unchanging windows under normal conditions. Relative to RSS-unchanging windows, PSI RSS-unfolding windows exhibited higher free energy and unpaired ratio values, whereas hairpin and internal loop counts were similar (Figures 3D and 3E; Supplemental Figure 6A and 6B). This pattern suggests that PSI RSS-unfolding windows contain shorter stems. Consistent with observations thus far, both the total and average stem lengths of PSI RSS-unfolding windows were shorter than those of control RSS-unchanging windows (Figure 3F; Supplemental Figure 6C). In summary, these results demonstrate that PSI RSS-unfolding predominantly occurs in regions with shorter stems and higher free energy.

To explore the roles of transcripts exhibiting PSI RSS unfolding, we performed GO enrichment analysis. PSI RSS-unfolding transcripts were enriched in multiple pathways, including responses to nutrient levels, P metabolism, transcription, RNA metabolism, translation, proteolysis, cell communication, and defense responses (Supplemental Figure 6D), suggesting that PSI RSS unfolding broadly affects cellular processes. Notably, transcripts of many known PSR genes were unfolded under –P conditions (Figure 3G). These included genes involved in systemic PSR signaling, such as *OsPHR1* -) (Zhou et al., 2008), *OsSPX1* (*Syg1/Pho81/XPR1*) (Wang et al., 2014), *OsVIP2* (Zhu et al., 2019), and *OsIPS2* (Hou et al., 2005); genes involved in Pi absorption and transfer, such as *OsPHF1* (Chen et al., 2011), *OsPT2* (Liu et al., 2010), and *OsPT8* (Jia et al., 2011); genes involved in organic Pi metabolism and Pi reuse, such as *OsACP1* (Deng et al., 2022) and *OsPAP26* (Gao et al., 2017); and genes involved in root system architecture remodeling, such as *OsPDR2* (Ticconi et al., 2009) and *OsARF16* (Shen et al., 2013) (Figure 3G). To validate –P-induced RSS unfolding in these key Pi homeostasis and PSR signaling genes, we modeled *in vivo* RSS based on DMS activity data. Indeed, all three examined RNA regions exhibited –P-mediated RSS unfolding, consistent with their DMS reactivity profiles (Figure 3H; Supplemental Figure 7). These results indicate an additional layer of PSR regulation at the RSS level.

### Global PSI RSS unfolding is not related to translational regulation in roots

**Figure 4:**
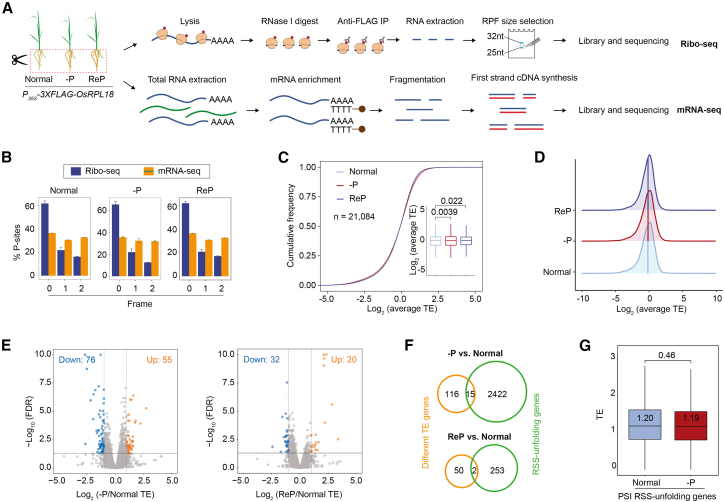
–P-specific RSS unfolding is not related to translational regulation (A) Schematic overview of the workflow for TRAP-based Ribo-seq. (B) Bar plots showing pronounced 3-nt periodicity at the 5′ ends of 28-nt RPFs (dark blue), but not at the 5′ ends of mRNA-seq reads (orange), across reading frames in normal, –P, and ReP samples. Error bars represent standard errors from biological replicates (*n* = 2). “0”, “1”, and “2” denote first, second, and third nucleotide positions, respectively, at the 5′ ends of the sequences. (C) Cumulative curves and boxplots showing comparable global TE under normal, –P, and ReP conditions. *p* values for boxplots were calculated using the Wilcoxon test. (D) Ridgeline plots showing comparable global TE distributions under normal, –P, and ReP conditions. (E) Volcano plots showing individual transcripts with altered TE under –P and ReP relative to normal conditions. Down, transcripts with significantly decreased TE; Up, transcripts with significantly increased TE. Numbers of transcripts with altered TE are shown. (F) Venn diagrams showing that few genes exhibited both altered TE and RSS unfolding under –P or ReP conditions. (G) Boxplots showing comparable global TE of genes with PSI RSS unfolding under normal and –P conditions. *p* values were calculated using the Wilcoxon test. –P, Pi deficiency; ReP, Pi-refeeding following Pi deficiency; TE, translational efficiency.

Ribosomes unwind mRNA secondary structures during translation, resulting in a global decline in RNA structure within highly translated transcripts, as observed in zebrafish (Beaudoin et al., 2018). This observation prompted us to investigate whether PSI RSS unfolding was coupled with increased translation. We performed translating ribosome affinity purification (TRAP)-based ribosome profiling (Ribo-seq) of rice roots under normal, –P, and ReP conditions (Figure 4A). We generated transgenic plants harboring constitutive 35S promoter–driven ribosomal protein L18 (RPL18) fused with 3×FLAG tag (*P*_*35S*_*-3×FLAG-OsRPL18*) in the Nip background. These transgenic plants did not display obvious differences in morphology or PSR relative to wild-type (WT) plants (Supplemental Figures 8A–8C, 8H, and 8I). We sampled roots of *P*_*35S*_*-3×FLAG-OsRPL18* plants and performed affinity purification of ribosome-RNA complexes using anti-FLAG antibodies. We then digested the samples with RNase I to obtain ribosome-protected fragments (RPFs) and performed high-throughput sequencing (Figure 4A; Supplemental Figure 8D; Supplemental Table 1). Corresponding strand-specific mRNA-seq was conducted in parallel (Figure 4A; Supplemental Table 1).

Clustering analysis and PCA demonstrated high reproducibility among biological replicates of each sample (Supplemental Figure 8E and 8F). Consistent with published data obtained by conventional ultracentrifugation-based Ribo-seq (Hsu et al., 2016), more than 95% of RPFs mapped to protein-coding regions in the Ribo-seq data (Supplemental Figure 8G). Additionally, more than 61% of the 5′ termini of 28-nt RPFs mapped to the first nucleotide of individual codons, exhibiting strong 3-nt periodicity (Figure 4B). These results support the high quality of the Ribo-seq data.

We calculated the translational efficiency (TE) of each mRNA, defined as the ratio of Ribo-seq reads to mRNA-seq reads for each protein-coding open reading frame (Bazin et al., 2017). –P did not induce obvious global changes in TE (Figures 4C and 4D; Supplemental Table 4). Relative to normal conditions, the TEs of 55 genes were significantly increased, whereas those of 76 genes were significantly decreased under –P conditions (|log_2_ fold change [FC]| ≥ 1, false discovery rate < 0.05) (Figure 4E; Supplemental Table 4). These findings are consistent with previous Ribo-seq results from *Arabidopsis thaliana* roots exposed to long-term (7-day) –P treatment (Bazin et al., 2017), in which fewer than 300 mRNAs displayed significant TE changes. We then evaluated the relationship between –P-mediated changes in RSS and TE. Fewer than 1% of –P-specific RSS-unfolding transcripts were associated with changes in TE under –P conditions (Figure 4F). Similar results were obtained for ReP relative to normal conditions (Figures 4E and 4F). Consistent with these observations, the global TEs of PSI RSS-unfolding transcripts did not differ between normal and –P conditions (Figure 4G). Overall, these results suggest that –P-specific RSS unfolding is not related to translational regulation.

### Regulation of global RNA stability in rice roots in response to –P

**Figure 5:**
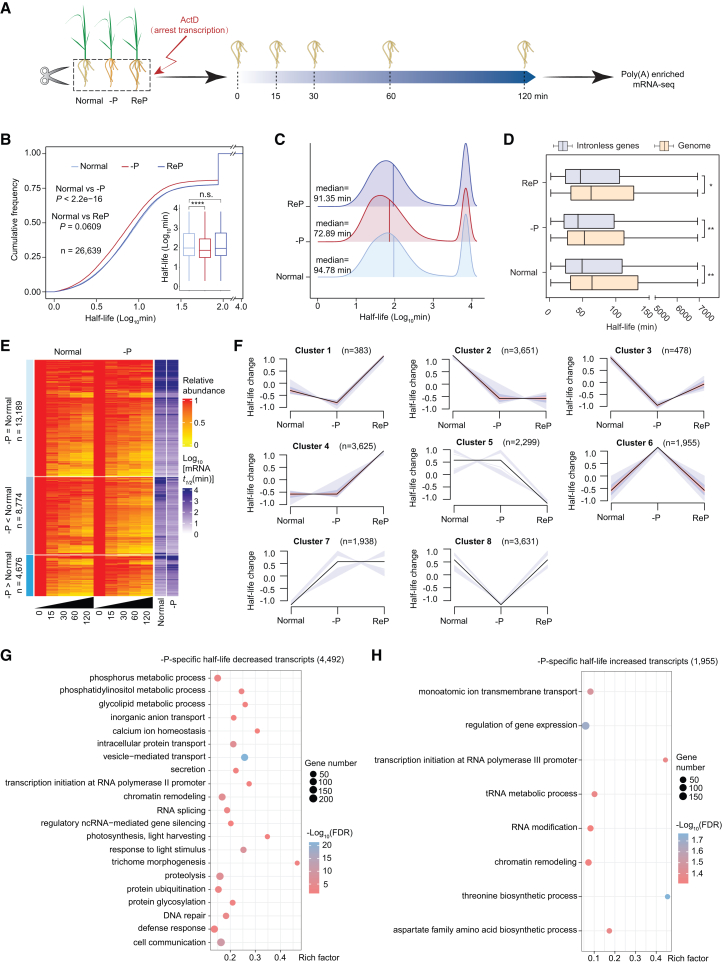
RNA stability–mediated PSR of rice roots (A) Schematic overview of the workflow for RNA decay analysis. (B) Cumulative curves and boxplots showing significantly reduced global RNA half-lives under –P compared with normal and ReP conditions. *p* values for cumulative curves were calculated using the Kolmogorov–Smirnov test; *p* values for boxplots were calculated using the Wilcoxon test. (C) Ridgeline plots showing decreased global RNA half-lives under –P compared with normal and ReP conditions. (D) Boxplots showing shorter half-lives of intronless genes (*t*_1/2_ < 6931 min) compared with the transcriptome (*t*_1/2_ < 6931 min) under normal, –P, and ReP conditions. *p* values for boxplots were calculated using the Wilcoxon test. ∗, *p* < 0.05; ∗∗, *p* < 0.01. (E) Heatmap showing RNA decay over 120 min in Nip under normal and –P conditions. RNA decay dynamics were quantified by tracking changes in mean relative RNA abundance over time, with decay rates expressed as RNA half-life (*t*_1/2_ in minutes). (F) Identification of gene clusters with –P-induced changes in half-life using the R package Mfuzz. (G and H) GO analysis of transcripts with –P-specific decreases (G) and increases (H) in half-life. In (B) and (D), midlines and box edges indicate medians and quartiles, respectively. Whiskers extend to the farthest data point within 1.5 times the IQR from the box edges. –P, Pi deficiency; ReP, Pi-refeeding following Pi deficiency.

Several studies have revealed that RSS regulates RNA stability (Yang et al., 2022b; Wu et al., 2024), prompting us to investigate the relationship between –P-induced RSS unfolding and RNA stability. We performed a transcriptome-wide RNA decay assay in rice roots under normal, –P, and ReP conditions (Figure 5A). Roots were infiltrated with the transcriptional inhibitor actinomycin D (ActD), sampled at 0, 15, 30, 60, and 120 min, and subjected to poly(A)-enriched RNA-seq (Sorenson et al., 2018) (Figure 5A). We obtained three highly reproducible biological replicates for each condition, with an average of 38 million reads per replicate (Supplemental Figure 9A and 9B; Supplemental Table 1). After normalization of sequencing data and removal of low-abundance mRNAs, we used transcript abundances to model decay rates for each gene (see methods). Similar to previous genome-wide studies of RNA decay in plants (Sorenson et al., 2018; Wu et al., 2024), we observed a broad range of mRNA half-lives (*t*_1/2_); the median mRNA half-life was 94.78 min under normal growth conditions (Figures 5B and 5C; Supplemental Table 5). Consistent with earlier findings that intronless mRNAs generally exhibit shorter half-lives than other mRNAs (Narsai et al., 2007; Wu et al., 2024), we observed the same pattern under all three growth conditions (Figure 5D). To further validate the fidelity of the RNA decay data, we randomly selected three transcripts with short, medium, and long half-lives under all three growth conditions; we confirmed their RNA stability via quantitative reverse-transcription PCR (RT-qPCR) (Supplemental Figure 9C and 9D). These results support the high fidelity and quality of the transcriptome-wide RNA decay dataset.

Global RNA stability was significantly reduced during –P treatment but was restored after ReP (Figures 5B and 5C). The median RNA half-life decreased to 72.89 min in response to –P treatment (Figure 5C), compared with 94.78 min under normal conditions, indicating that –P inhibits global RNA stability. The half-lives of 8,774 transcripts were shortened, whereas those of 4,676 transcripts were prolonged, under –P compared with normal conditions (Figure 5E). As expected, transcripts with shortened or prolonged half-lives exhibited significant overlap between the –P vs. normal and –P vs. ReP comparisons (Supplemental Figure 10). We subsequently applied soft clustering—using the Mfuzz algorithm (fuzzy c-means) with Euclidean distance—to RNA half-life values across normal, –P, and ReP conditions to group transcripts whose half-lives were altered by –P and restored by Pi refeeding. These transcripts were classified as –P-specific half-life–decreased transcripts (4,492 genes in clusters 1, 3, and 8) or –P-specific half-life–increased transcripts (1,955 genes in cluster 6) (Figure 5F; Supplemental Table 5). GO analysis revealed that –P-specific RNA stability–decreased genes were enriched in pathways including phosphatidylinositol metabolism, glycolipid metabolism, anion transport, transcription by RNA polymerase (Pol) II, RNA splicing, RNA silencing, photosynthesis, proteolysis, protein transport, DNA repair, and cell communication (Figure 5G). In contrast, –P-specific RNA stability–increased genes were enriched in GO terms related to transcription by RNA Pol III, tRNA metabolism, RNA modification, and amino acid biosynthesis (Figure 5H). Taken together, these results indicate that RNA stability mediates the PSR in rice roots.

### Downregulation of RNA half-life and RSS in roots under –P

**Figure 6:**
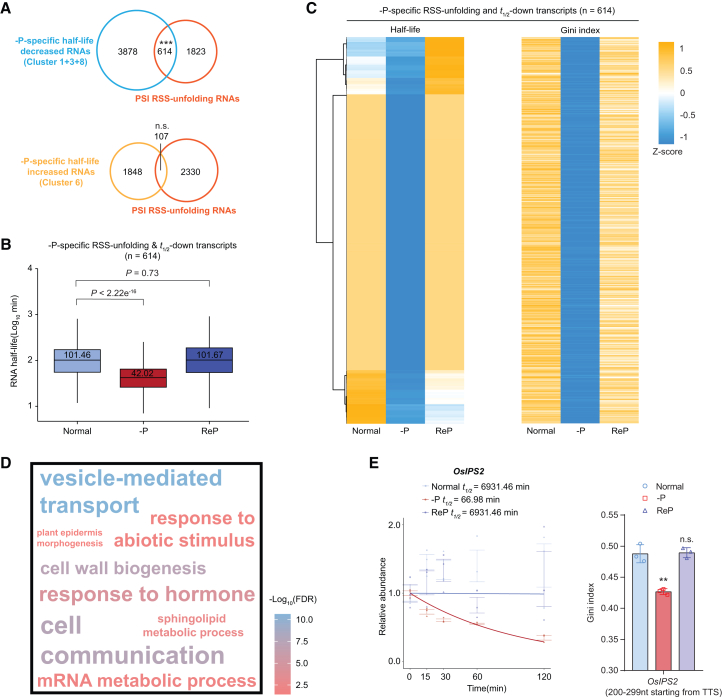
Synergistic downregulation of RNA stability and RSS under –P conditions (A) Venn diagrams showing significant overlap of PSI RSS-unfolding RNAs with –P-specific RNAs exhibiting reduced half-lives, but not with –P-specific RNAs displaying increased half-lives. *p* values were calculated using a hypergeometric test. (B) Boxplots showing significant global decreases in half-lives of –P-specific RSS-unfolding & *t*_1/2_-down transcripts under –P, but not ReP, compared with normal conditions. *p* values were calculated using the Wilcoxon test. Midlines and box edges indicate medians and quartiles, respectively. Whiskers extend to the farthest data point within 1.5 times the IQR from the box edges. (C) Heatmap showing coordinated downregulation of RNA stability and RSS for –P-specific RSS-unfolding & *t*_1/2_-down transcripts under –P conditions. (D) GO analysis of –P-specific RSS-unfolding & *t*_1/2_-down transcripts. Font size indicates the number of associated transcripts. (E) Both RNA half-life profiles and Gini indices of *OsIPS2* decreased in response to –P and were restored after ReP. In half-life profiles, relative RNA abundances following transcriptional inhibition are shown, with bars indicating means ± standard error of the mean (*n* = 3); thick lines indicate modeled values. Half-lives (*t*_1/2_) are indicated for each treatment. In Gini index bar plots: TTS, transcription termination site; *p* values were calculated using an unpaired two-tailed Student’s *t* test. ∗∗, *p* < 0.01; n.s., not significant. –P, Pi deficiency; ReP, Pi-refeeding following Pi deficiency.

We evaluated the relationship between –P-induced changes in RNA stability and –P-specific changes in RSS. Global RNA half-lives of PSI RSS-unfolding transcripts decreased under –P but were restored after ReP (Supplemental Figure 11A). Notably, the magnitude of the decrease in half-life was greater for these transcripts (median reduction of 26.33 min) than for the transcriptome overall (median reduction of 21.89 min), suggesting that RSS unfolding contributes to RNA decay (Figure 5C; Supplemental Figure 11A). Consistent with this interpretation, we observed substantial overlap between PSI RSS-unfolding transcripts and transcripts with decreased RNA half-lives under –P, but not with those exhibiting increased half-lives (Figure 6A). These 614 overlapping transcripts were designated –P-specific RSS-unfolding & *t*_1/2_-down transcripts (Supplemental Table 5). Similar to the Gini index, the global half-lives of these transcripts were significantly reduced in response to –P and were fully restored after ReP (Figures 6B and 6C).

GO analysis showed that these –P-specific RSS-unfolding & *t*_1/2_-down transcripts are involved in responses to abiotic stimuli, hormone responses, sphingolipid metabolism, mRNA metabolism, vesicle-mediated transport, and other processes (Figure 6D). Among these transcripts, we identified several known PSR genes that participate in Pi homeostasis and PSR signaling, such as *OsIPS2*, *OsSPX1*, *OsPAP9a*, and *OsPT15* (Figure 6E; Supplemental Figure 11B–11D; Supplemental Table 5). Several other genes, including the root hair formation- and tillering–related gene *OsRBOHE* (*Respiratory Burst Oxidase Homolog E*) (Zhao et al., 2025), the RNA decay–related gene *OsXRN3* (5′–3′ exoribonuclease) (Han et al., 2023), and the potassium transfer- and drought stress response–related gene *OsHAK1* (*High-affinity Potassium Transporter 1*) (Chen et al., 2015a, 2017), also exhibited concurrent reductions in RNA stability and Gini index under –P conditions (Supplemental Figure 11E−11G). These results indicate a broad role for –P-induced RSS unfolding in RNA stability regulation during Pi starvation.

### PSI RSS unfolding represses RNA stability to fine-tune RNA expression and Pi homeostasis

**Figure 7:**
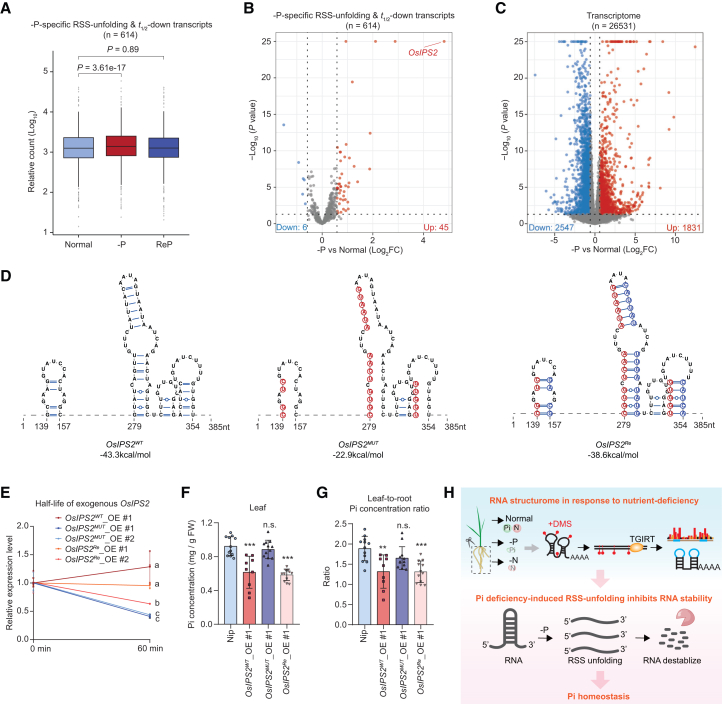
–P-specific RSS unfolding represses RNA stability to fine-tune steady-state PSR transcript levels and Pi homeostasis (A) Boxplots showing a moderate global increase in steady-state levels of –P-specific RSS-unfolding & *t*_1/2_-down transcripts under –P, but not ReP, compared with normal conditions. Midlines and box edges indicate medians and quartiles, respectively. Whiskers extend to the farthest data point within 1.5 times the IQR from box edges. (B and C) Volcano plots showing expression changes in –P-specific RSS-unfolding & *t*_1/2_-down transcripts (B) and across the transcriptome (C). Up, upregulated transcripts (–P vs. normal, log_2_FC > 0.585, *p* < 0.05); down, downregulated transcripts (–P vs. normal, –log_2_FC > 0.585, *p* < 0.05). Differential expression analysis was performed using DESeq2, with *p* values calculated via the Wald test. (D) Modeled RSS of PSI RSS-unfolding regions (139–157 and 279–354 nt from the TSS) of *OsIPS2* (*OsIPS2*^*WT*^) and corresponding RSS-mutated (*OsIPS2*^*MUT*^) and RSS compensatory-mutated (*OsIPS2*^*Re*^) variants. Predicted ΔG values are shown. RSS-mutated nucleotides are highlighted by red circles, and compensatory-mutated nucleotides are highlighted by blue circles. (E) Target-specific RNA decay showing half-lives of exogenous *OsIPS2* in *OsIPS2*^*WT*^_OE, *OsIPS2*^*MUT*^_OE, and *OsIPS2*^*Re*^_OE transgenic lines. Relative expression levels of transcripts were normalized to the 0-min time point (ratio arbitrarily set to 1), with standard deviations calculated from three biological replicates. Different lowercase letters indicate significant differences based on an unpaired two-tailed Student’s *t* test (*p* < 0.05). (F and G) Leaf Pi concentration (F) and leaf-to-root Pi concentration ratio (G) in Nip, *OsIPS2*^*WT*^_OE #1, *OsIPS2*^*MUT*^_OE #1, and *OsIPS2*^*Re*^_OE #1 plants. ∗∗, *p* < 0.01; ∗∗∗, *p* < 0.001; unpaired two-tailed Student’s *t* test. n.s., not significant. (H) Schematic summarizing the high-quality RNA structurome under nutrient-deficient conditions, indicating that Pi-starvation–induced RSS-unfolding reduces RNA stability and contributes to fine-tuning Pi homeostasis in rice roots. –P, Pi deficiency; ReP, Pi-refeeding following Pi deficiency; –N, nitrogen deficiency; DMS, dimethyl sulfate.

Next, we examined the levels of –P-specific RSS-unfolding & *t*_1/2_-down transcripts. Compared with normal conditions, global steady-state levels of these transcripts significantly increased under –P conditions and returned to basal levels after ReP (Figure 7A). This apparent discrepancy between decreased RNA stability and increased steady-state abundance indicates that feedback regulation mediated by RSS unfolding affects gene expression. Although expression changes for approximately 92% of these transcripts were less than 1.5-fold (–P vs. normal, log_2_[FC] < 0.585, *p* < 0.05) (Figure 7B), RNA levels of the majority of –P-specific RSS-unfolding & *t*_1/2_-down transcripts (∼68%) tended to increase under –P compared with normal conditions (–P vs. normal, log_2_[FC] > 0) (Figure 7B). This pattern was not evident at the transcriptome-wide level (Figure 7C). Instead, a slight overall decrease in expression was detected; 1,831 and 2,547 transcripts were significantly upregulated and downregulated, respectively, by more than 1.5 fold (–P vs. normal, log_2_|[FC]| ≥ 0.585) under –P conditions (Figure 7C). The inverse relationship between expression level and RNA stability of –P-specific RSS-unfolding transcripts suggests that PSI RSS unfolding represses RNA stability, providing feedback to fine-tune steady-state transcript levels.

To validate the role of PSI RSS unfolding in RNA stability and Pi homeostasis, we performed RSS mutagenesis and generated transgenic plants to examine PSI RSS-unfolding–mediated changes in RNA stability *in vivo*. Considering potential side effects of RSS mutations on amino acid sequences and the feasibility of functional assessment, we selected the well-characterized –P-induced lncRNA *OsIPS2*. This transcript represents a –P-specific RSS-unfolding & *t*_1/2_-down RNA (Figures 3H and 6E) whose expression is induced by OsPHR2 under –P conditions (Figure 7B) (Zhou et al., 2008). The –P-induced RSS-unfolding regions of *OsIPS2*, located at nucleotides 139–157 and 279–354 from the transcription start site (TSS) and excluding the miR399-binding region (Franco-Zorrilla et al., 2007), form four stem-loop structures under normal conditions but adopt a single-stranded conformation under –P stress (Figure 3H). We introduced point mutations into this region to mimic the –P-induced single-stranded conformation (*IPS2*^*MUT*^), along with compensatory mutations to restore the RSS of this region to the stem-loop structure (*IPS2*^*Re*^) (Figure 7D). The minimum thermodynamic free energy (Δ*G*) of these PSI RSS-unfolding regions increased from −43.3 kcal/mol (*OsIPS2*^*WT*^) to −22.9 kcal/mol (*OsIPS2*^*MUT*^) and was restored to −38.6 kcal/mol (*OsIPS2*^*Re*^) by compensatory mutation (Figure 7D), indicating successful modulation of RSS.

We overexpressed full-length WT, mutated (MUT), and compensatory mutated (Re) *OsIPS2* transcripts driven by the 35S promoter to generate stable transgenic plants. We randomly selected one *OsIPS2*^*WT*^_OE line and two individual *OsIPS2*^*MUT*^_OE and *OsIPS2*^*Re*^_OE lines to measure RNA half-lives of exogenously expressed *OsIPS2* under normal conditions. Compared with the stable RNA half-life of *OsIPS2*^*WT*^, the half-life of *OsIPS2*^*MUT*^ (harboring an unfolded RSS) was significantly reduced (Figure 7E), whereas the half-life of *OsIPS2*^*Re*^ (harboring a restored stem-loop RSS) was comparable to that of *OsIPS2*^*WT*^ (Figure 7E). Half-lives of the control unstable transcript (Os01g0389200) were similar across all examined transgenic lines, indicating effective transcriptional arrest via chemical treatment (Supplemental Figure 12A). In parallel, compared with *OsIPS2*^*WT*^, the steady-state expression of *OsIPS2*^*MUT*^ was significantly reduced in *OsIPS2*^*MUT*^_OE plants, whereas expression of the control gene *HPT* was significantly elevated (Supplemental Figure 12B and 12C). The opposite trend was observed in *OsIPS2*^*Re*^_OE transgenic plants (Supplemental Figure 12B and 12C). Collectively, these results confirm that –P-induced RSS unfolding represses RNA stability.

*OsIPS1/2* serve as key repressors of Pi translocation from roots to shoots (Franco-Zorrilla et al., 2007). Consistent with earlier findings, we observed significant decreases in leaf Pi concentration and the leaf-to-root Pi concentration ratio in both *OsIPS2*^*WT*^_OE and *OsIPS2*^*Re*^_OE plants relative to Nip (Figures 7F and 7G). In contrast, *OsIPS2*^*MUT*^_OE plants exhibited leaf Pi concentrations and leaf-to-root Pi ratios comparable to those of Nip (Figures 7F and 7G). These results indicate that –P-induced RSS unfolding regulates Pi homeostasis (Figure 7H).

## Discussion

Rice is a staple cereal crop that sustains more than half of the world’s population (FAO, 2018; Sen et al., 2020). Nutrient deficiency remains a major constraint on global rice production and quality (Fahad et al., 2019). Elucidation of the mechanisms underlying crop responses to fluctuations in nutrient availability is essential for developing varieties with high nutrient-use efficiency. In this study, we obtained high-quality, deep-coverage *in vivo* RNA structuromes from rice roots under normal and nutrient-deficient conditions (–P and –N) using optimized DMS-MaPseq methods (Figure 1). High-conf. RSS information covered more than 60% of expressed transcripts under each growth condition. –P induced global increases in RSS diversity and unfolding, predominantly within CDS regions of mRNAs (Figures 2 and 3). To investigate the regulatory functions of PSI RSS unfolding, we performed translatome profiling and transcriptome-wide RNA decay analyses under –P stress (Figures 4 and 5). Integrated multi-omics analyses and studies of RSS-mutated transgenic plants revealed that repression of PSI RSS-unfolding–mediated RNA stability fine-tunes the expression of PSR-related transcripts and Pi homeostasis under –P conditions (Figures 6 and 7). Collectively, these findings highlight a regulatory role for RSS-unfolding–mediated RNA decay in the PSR of rice roots.

*In vivo* RNA structure is dynamic and influenced by the cellular physicochemical environment (Bevilacqua et al., 2016; Vandivier et al., 2016; Wang et al., 2021). Using *in vivo* RSS probing technologies, several recent studies have investigated RNA structure reprogramming in response to abiotic and biotic stresses in plants (Zhu et al., 2021; Zhang and Ding, 2025). Our high-quality, in-depth RNA structurome data revealed dynamic alterations of *in vivo* RSS in rice roots under –P and –N conditions. In particular, –P triggered widespread RSS unfolding (Figure 2). Transcripts harboring PSI RNA-unfolding regions participate in multiple PSR processes (Figure 3G), as well as RNA metabolism, translation, and vesicle-mediated transport (Supplemental Figure 6D), indicating broad functions of RSS in plant adaptation to –P stress. Feature analysis showed that PSI RNA-unfolded regions exhibit low GC content and short stem length, consistent with flexible RSS (Figure 3). Similarly, –N-induced RSS-unfolding regions also displayed low GC content and flexible RSS features (Supplemental Figures 5A–5C). These observations are consistent with previous genome-wide RSS profiling studies using Structure-seq in *Arabidopsis* (Ding et al., 2014; Tack et al., 2020) and rice (Deng et al., 2018), which detected that mRNAs with highly flexible RSS tend to be associated with plant responses to environmental stimuli. Although RSS features share common characteristics in –P- and –N-induced RSS remodeling, PSI- and –N-unique RSS-unfolding transcripts were enriched in distinct stress-specific biological processes (Supplemental Figures 5E and 6D), suggesting divergent regulatory roles of RSS unfolding under different stress conditions.

Notably, the –P-induced transcriptome-wide increase in RSS diversity suggests that PSI RNA unfolding is an active, rather than passive, process (Figure 2). Ribo-seq further indicated that PSI RNA unfolding is not a secondary consequence of enhanced translation (Figure 4). It is possible that currently uncharacterized PSR-related RNA-binding proteins (RBPs) and/or RNA helicases contribute to this process. Indeed, we found that several RNA helicase- and RBP-encoding genes were significantly upregulated under –P conditions (Supplemental Figure 13), making them promising candidates for future functional studies. Additionally, RNA conformation is influenced by cations, heavy metals, and diverse metabolites (Bevilacqua et al., 2016). Given that P is a key element for the biosynthesis of essential metabolites, –P causes substantial changes in primary and secondary metabolism in plants (Pant et al., 2015; Dissanayaka et al., 2021). These metabolic alterations might also contribute to global changes in RSS. Taken together, these mechanisms may underlie the specific RSS responses observed under –P and –N nutritional stresses.

RNA structure is crucial for governing nearly all aspects of RNA metabolism (Xu et al., 2022). Genome-wide RNA decay assays and analyses of transgenic plants exhibiting RSS mutagenesis revealed RNA structure–mediated regulation of PSR in rice roots (Figures 5, 6, and 7). Accelerated Pi recycling from organic forms is an important PSR in plants (Paz-Ares et al., 2022), but the underlying mechanisms remain underexplored. RNA constitutes a major intracellular P pool. Endonucleases, such as S-like RNases (RNSs), are upregulated upon Pi starvation to degrade cytosolic or extracellular RNAs (Supplemental Figure 1D) (Bariola et al., 1994; Gho et al., 2020). Indeed, our transcriptome-wide RNA decay assay revealed a decrease in global RNA half-life under –P conditions (Figure 5). Notably, PSI RSS-unfolding predominantly occurred within CDS regions, rather than UTRs. This pattern differs from the RNA decay driven by temperature-induced RSS unfolding at UTR regions in *Arabidopsis* (Yang et al., 2022b) and rice (Su et al., 2018), suggesting that CDS regions exert regulatory functions through RSS beyond their canonical role in protein encoding. Furthermore, –P-induced RNSs belong to the RNase T2 family, which comprises nonspecific single-stranded RNA endonucleases (Megel et al., 2019). PSI RSS-unfolding within CDS regions could enhance transcript accessibility to RNSs, thereby facilitating RNA degradation. In addition to endonuclease induction, RSS unfolding within CDS regions might represent an additional regulatory mechanism that accelerates RNA decay and Pi recycling under –P conditions.

Furthermore, global steady-state levels of transcripts with reduced RNA stability due to PSI RSS-unfolding increased under –P conditions (Figure 7A). These transcripts included many important PSR- and stress response–related genes (Figure 6; Supplemental Figure 11). Our analysis of transgenic plants carrying *OsIPS2* RSS mutations confirmed that PSI RSS-unfolding decreases RNA stability and attenuates its function in Pi translocation from roots to shoots (Figure 7D–7G). These findings suggest that feedback mediated by PSI RSS-unfolding fine-tunes the expression of key PSR transcripts, thereby maintaining appropriate root responses to Pi deficiency. Such RSS-mediated feedback regulation may also enable rapid downregulation of pivotal PSI transcripts after Pi refeeding. Future validation of additional PSI RSS-mediated functions in other PSR-related transcripts, including non-coding RNAs, could help identify candidate RSS elements for enhancing low-Pi tolerance. Researchers could use CRISPR-Cas9–based precise mutagenesis to modify PSR-related RSS regions, modulating transcript stability and thereby enhancing nutrient uptake in rice.

In conclusion, we generated a high-quality, high-coverage RNA structurome of rice roots under normal and nutrient-deficient conditions. Integrated analyses of RSS features, the translatome, and transcriptome-wide RNA decay assays, along with analyses of transgenic plants exhibiting RSS mutagenesis, uncovered repressed RSS-unfolding–mediated RNA stability as an adaptive response of rice to Pi deficiency stress. These findings provide novel insights into the regulatory roles of *in vivo* RSS in mediating plant responses to nutrient deficiency. Additionally, the transcriptome-wide *in vivo* RSS landscape under nutrient-deficient conditions represents a valuable resource for related crop studies and offers potential targets for crop improvement.

## Methods

### Plant materials and growth conditions

The *japonica* rice (*Oryza sativa* subsp. *japonica*) cultivar Nip was used in this study. Seeds were soaked in 1% (v/v) HNO_3_ solution for 16 h to break dormancy, germinated in pure water at 37°C for 3 days, and then cultured hydroponically. Germinated seedlings were grown in a hydroponic nutrient solution (0.8225 mM NH_4_NO_3_, 0.2 mM NaH_2_PO_4_·2H_2_O, 0.1915 mM K_2_SO_4_, 0.547 mM MgSO_4_·7H_2_O, 0.366 mM CaCl_2_·2H_2_O, 0.0005 mM MnCl_2_·4H_2_O, 0.003 mM H_3_BO_3_, 0.0001 mM (NH_4_)_6_Mo_7_O_24_·4H_2_O, 0.0004 mM ZnSO_4_·7H_2_O, 0.0002 mM CuSO_4_·5H_2_O, 0.04 mM EDTA-Fe, and 0.2 mM MES; pH adjusted to 5.5 with 5 M NaOH) (Yoshida et al., 1976).

For the RSS mutagenesis assay, Nip plants were transformed with the binary vectors P_35S_-OsIPS2^WT^, P_35S_-OsIPS2^MUT^, and P_35S_-OsIPS2^Re^ by *Agrobacterium tumefaciens* (strain EHA105)–mediated transformation (Toki et al., 2006) to generate the corresponding *OsIPS2*^*WT*^_OE, *OsIPS2*^*MUT*^_OE, and *OsIPS2*^*Re*^_OE transgenic lines. Positive transgenic plants were confirmed by genomic PCR in the T0 generation, followed by RT-qPCR in the T1 generation to verify exogenous expression of *OsIPS2*. The transgenic line *P*_*35S*_*-3×FLAG-OsRPL18* in the Nip background was generated in a similar manner for TRAP-based Ribo-seq (described in the following subsection: Construction of P35S-3×FLAG-OsRPL18). Transgenic lines in the T1 generation were identified by immunoblotting using an anti-FLAG antibody (ABclonal, cat# AE005).

For hydroponic culture, rice seedlings were grown in a greenhouse under a 14-h light (30°C)/10-h dark (24°C) photoperiod, with approximately 300 μmol m^−2^ s^−1^photon flux density and ∼60% relative humidity. Ten-day-old seedlings were subjected to normal (200 μM Pi, 822.5 μM N), Pi-deficient (–P, 0 μM Pi, 822.5 μM N), or N-deficient (–N, 200 μM Pi, 41.125 μM N) conditions for 5 days. For Pi refeeding (ReP), –P-treated seedlings were transferred to normal conditions and cultured for an additional 2 days.

### Vector construction

#### *Construction of P*_*35S*_*-OsIPS2*^*WT*^, *P*_*35S*_*-OsIPS2*^*MUT*^, *and P*_*35S*_*-OsIPS2*^*Re*^

Full-length *OsIPS2* carrying an RSS mutation (*OsIPS2*^*MUT*^) or a compensatory RSS mutation (*OsIPS2*^*Re*^) was synthesized (GeneScript); the corresponding sequences are provided in Supplemental Table 6. Full-length *OsIPS2*^*MUT*^ and *OsIPS2*^*Re*^ fragments were amplified from the synthesized plasmids. The full-length *OsIPS2*^*WT*^ sequence was amplified from Nip cDNA using KOD-Plus-Neo Hot Start polymerase (TOYOBO). PCR products of *OsIPS2*^*WT*^, *OsIPS2*^*MUT*^, and *OsIPS2*^*Re*^ were inserted into the BamHI/SalI-digested pCAMBIA1300-P_35S_ vector to generate the P_35S_-OsIPS2^WT^, P_35S_-OsIPS2^MUT^, and P_35S_-OsIPS2^Re^ constructs, respectively.

#### *Construction of P*_*35S*_*-3×FLAG-OsRPL18*

The CDS of *OsRPL18* (Os05g0155100) was amplified from Nip cDNA using KOD-Plus-Neo Hot Start polymerase (TOYOBO) and ligated into pCAMBIA-P_35S_-3×FLAG to obtain P_35S_-3×FLAG-OsRPL18.

All constructs were confirmed by sequencing. Primers used to generate all constructs are listed in Supplemental Table 6.

### Measurement of Pi and total P contents

Cellular Pi and total P concentrations in rice roots and leaves were measured as previously described (He et al., 2021). Briefly, cellular Pi concentrations were determined using a continuous flow analyzer (SAN++, SKALAR, Breda, the Netherlands). Total P concentrations were measured by Inductively Coupled Plasma–Optical Emission Spectrometry (Optima 7300DV; PerkinElmer, Waltham, MA, USA).

### Measurement of nitrogen content

Root samples were harvested and dried at 65°C for 1 week. Dried samples were ground to a fine powder using a tissue grinder (JINXIN). Total N content was determined by Elemental Analysis–Isotope Ratio Mass Spectrometry (EA-IRMS; Thermo Scientific Flash 2000/Delta V Advantage).

### *In vivo* DMS modification

*In vivo* DMS modification was performed as previously described (Wang et al., 2019; Jin et al., 2022). Briefly, after nutrient-deficient treatments, the roots of rice seedlings were collected and cut into ∼1.5-cm segments. Samples were immersed in 20 mL of 1× DMS reaction solution (40 mM HEPES pH 7.5; 100 mM KCl; and 0.5 mM MgCl_2_ in DEPC-treated H_2_O) in a 50-mL Corning tube. After 200 μL DMS (MACKIN) had been added to a final concentration of 1%, the reaction solution was vigorously vortexed until the DMS was fully dissolved. Mock treatments were performed by adding an equal volume of deionized water instead of DMS. Samples were treated in DMS reaction buffer or mock solution at room temperature (RT) for 5 min under vacuum (∼12 psi), then incubated at 30°C with shaking at 250 rpm for 10 min. After addition of 5 mL β-mercaptoethanol (Sigma) to quench the DMS reaction, samples were incubated for 5 min under vacuum at RT. Samples were washed three times with 50 mL DEPC-treated H_2_O, blotted dry with paper towels, and immediately frozen in liquid nitrogen. Three biological replicates were prepared for each DMS-treated sample, and one biological replicate was prepared for each mock-treated sample.

All manipulations involving DMS were conducted using appropriate safety equipment, including lab coats and double gloves. All disposable materials were discarded as hazardous waste. DMS treatments were performed in a chemical fume hood with strong airflow (>200 feet per minute).

### Genome-wide DMS-MaPseq library generation and sequencing

Genome-wide DMS-MaPseq library generation was performed as previously described, with minor modifications (Wang et al., 2018, 2019; Jin et al., 2022). DMS-treated and untreated root samples were ground into powder; total RNA was isolated using TRIzol reagent (Thermo Fisher) in accordance with the manufacturer’s instructions. Total RNA was treated with TURBO DNase (Thermo Fisher) and purified using an RNeasy Mini kit (Qiagen). Each 1 μg DNase-treated RNA sample was processed using the TruSeq Stranded Total RNA Sample Prep Kit (Illumina). rRNA was removed using the Ribo-Zero Plant Kit (Illumina). For reverse transcription, TGIRT-III enzyme (InGex) was used instead of SuperScript II. Illumina sequencing adaptors and indices were introduced by performing no more than 15 cycles of PCR amplification. Genome-wide DMS-MaPseq libraries were sequenced in 2 × 150-nt paired-end mode on the NovaSeq platform at Novogene.

### DMS-MaPseq data analysis

An average of 600 million paired-end 150-nt raw reads were obtained for each DMS-MaPseq library. TrimGalore (v0.6.6) (Martin, 2011) was used to remove the Illumina universal adaptor sequence (AGATCGGAAGAGCACACGTCTGAACTCCAGTCA) and to filter low-quality reads (quality score <25). For TGIRT-generated libraries, 2-nt sequences were trimmed from the 5′ end (Zubradt et al., 2017). The “Quality Filter” function of the FASTX-Toolkit (v0.0.14) (Gordon and Hannon, 2010) was used for additional filtering; at least 80% of bases were required to display a quality score >25.

TopHat2 (v2.1.1) (Kim et al., 2013) was used to align reads to the IRGSP-1.0 reference genome (Kawahara et al., 2013). The following parameters were applied: “tophat2 -p 20 --library-type fr-firststrand --no-novel-juncs -N 15 --read-gap-length 10 --read-edit-dist 15 --max-insertion-length 5 --max-deletion-length 5 -g 3,” allowing up to 10% mismatches. Uniquely mapped reads were selected using “grep -E “ˆ@|NH:i:1.”

To prevent overlap of mismatch signals derived from forward- and reverse-strand transcripts at the same genome positions, the “view” function of SAMtools (v1.9) (Li et al., 2009) was used to separate uniquely mapped BAM files into forward- and reverse-strand files according to SAM flags.

Mismatch and coverage counts for each nucleotide were calculated using the in-house Python script “CountMismatch2Bed.py” (https://github.com/changhaoli/TAMU_02RSS) and the BEDTools (v2.29.2) (Quinlan and Hall, 2010) “genomecov” function with the parameters “-d -split.” Raw DMS reactivity was calculated by dividing mismatch counts by coverage at each nucleotide position. Genome-wide DMS reactivity was assumed to be comparable across nutrient conditions. Therefore, DMS reactivity values for each sample were normalized by the ratio of the average DMS reactivity in that sample to the average DMS reactivity in samples under normal conditions.

### Analysis of changes in RSS

To identify transcript regions with significant RNA structural differences across conditions, a sliding window approach was used to calculate the Gini index across the transcriptome, as previously described (Zubradt et al., 2017) with minor modifications. Specifically, each gene was divided into non-overlapping 100-nt windows according to genomic coordinates (from smallest to largest positions) based on the IRGSP-1.0 reference genome. For genes located on the positive strand, window segmentation began at the annotated TSS and proceeded sequentially in the 5′ to 3′ direction of the transcript. For genes on the negative strand, window partitioning began at the annotated transcription termination site (TTS) and extended along increasing genomic coordinates, corresponding to the 3′ to 5' orientation of the transcript. Only windows with an average minimum of 20 mismatch counts per nucleotide, restricted to A and C mismatches, were used to calculate the Gini index.

For each window, two metrics were calculated to identify RSS alterations across conditions. First, the average Gini index for each window was calculated across three biological replicates under each condition. The change in Gini index (ΔGini) was then computed as the Gini index of the nutrient-deficient sample minus the Gini index of the normal sample. Second, the statistical significance of Gini index differences between conditions was determined by Student’s *t* test. Thresholds used to define significant differential windows were a *p* value < 0.05 and |ΔGini| ≥ 0.05. The 100-nt windows in regions with structural changes spanning both CDS and UTRs were classified as UTR regions.

### Secondary structure modeling

Raw DMS-induced mismatch ratios were normalized prior to RNA structure modeling (Lan et al., 2022). All raw DMS reactivities were divided by the median of the highest 5% of mutation rates to obtain normalized DMS reactivities. Normalized reactivities exceeding 1.0 were winsorized by setting values to 1.0.

RSS was predicted from normalized DMS reactivity values using the fold function of RNAstructure (v6.1) (Reuter and Mathews, 2010). To minimize noise signals not derived from A and C, DMS reactivities for guanine and thymine bases were set to −999 (unavailable constraints).

RNA structures were visualized using VARNA (v3-93) (Darty et al., 2009). Bases were color-coded according to normalized DMS reactivities.

### Analysis of *in vivo* RSS diversity

Alternative RSS conformations of transcripts were detected across the transcriptome using DREEM (Tomezsko et al., 2020). As in the Gini index analysis, the transcriptome (TIGR reference) was divided into 100-nt windows. Only windows covered by more than 50,000 or 25,000 reads were analyzed by DREEM with default parameters. For each window, iteration results yielding the highest number of clusters were used for subsequent analyses.

To evaluate RSS diversity, the Shannon diversity index was calculated as follows:

$$\text{Shannon}\;\text{index}=-\sum_{i=1}^{c}p_{i}\;\ln\;p_{i}$$

Here, *c* is the cluster number (1, 2, 3, or 4), and *p*_*i*_is the percentage of *i* clusters.

### RNA secondary structure feature analysis

To examine RNA secondary structure features of PSI RSS-unfolding windows, structures for each 100-nt window were modeled using RNAstructure (v6.1) (Reuter and Mathews, 2010), with corresponding FASTA sequences and normalized DMS reactivity data as inputs. Predicted structures were output in dot-bracket notation, then analyzed using ViennaRNA/forgi (v2.1.1) (Thiel et al., 2019) to quantify RSS features.

### Ribo-seq library construction and sequencing

TRAP-based Ribo-seq was performed as previously described (Juntawong et al., 2015). Library construction comprised three major steps: ribosome-associated RNA isolation, small-RNA library preparation, and PCR amplification.

Normal, –P-, and ReP-treated *P*_*35S*_*-3×FLAG-OsRPL18* rice roots were harvested and ground to powder in liquid nitrogen. To isolate ribosome-associated RNA, 0.4 g of powdered sample was lysed in 2 mL polysome extraction buffer (20 mM Tris-HCl pH 7.4, 150 mM NaCl, 5 mM MgCl_2_, 1% Triton X-100, 1 mM DTT, 100 μg/mL cycloheximide [Sigma], and 1 pellet/50 mL complete EDTA-free protease inhibitor). After incubation on ice for 10 min and centrifugation at 16,000 × g for 15 min at 4°C, the supernatant was treated with RNase I (Epicentre) to digest exposed RNA regions and to dissociate polysomes into monosomes. After addition of 18 μL SUPERase-In RNase inhibitor (Thermo Fisher) to terminate the reaction, anti-FLAG antibody–conjugated magnetic beads were added to the supernatant; the mixture was then incubated for 2 h with gentle rocking at 4°C to enrich monosomes. Enriched beads were washed three times with wash buffer (20 mM Tris-HCl pH 8.0, 140 mM KCl, 35 mM MgCl_2_, 50 μg/mL cycloheximide, 50 μg/mL chloramphenicol, and 1 pellet/50 mL complete EDTA-free protease inhibitor). Subsequently, TRIzol (Thermo Fisher) was used for RPF extraction. RPFs were resolved by electrophoresis on a 10% urea-PAGE gel. Cy3-labeled 25- and 35-nt RNA markers were used to guide size selection, and RPFs were recovered via gel extraction.

The RPFs were treated with T4 polynucleotide kinase (New England Biolabs) to remove 3′ phosphoryl groups. Following dephosphorylation, RNA fragments were ligated to 3′ DNA adaptors using T4 RNA ligase 2 (truncated KQ). After gel excision to remove free 3′ DNA adaptors, cDNA was synthesized using SuperScript II reverse transcriptase with a Ribo-seq RT primer. rDNA was depleted from cDNA using a streptavidin-biotin approach with biotinylated in-house anti-rRNA probes and Dynabeads MyONE magnetic beads C1 (Thermo Fisher). rDNA-depleted cDNA was circularized using CircLigase (Lucigen) and amplified with KOD-Plus-Neo Hot Start polymerase (TOYOBO). PCR products were digested with PmeI (New England Biolabs) to remove 25- and 35-nt RNA markers derived from the original library.

Finally, PCR products were purified by 3% agarose gel purification and sequenced in 2 × 150-nt paired-end mode on the Illumina NovaSeq platform at Annoroad. Sequences of the 3′ adaptor, Ribo-seq RT primer, 5′ PCR primer, and 3′ PCR primer used in this study are listed in Supplemental Table 6.

### Ribo-seq data analysis

Adaptor trimming and removal of low-quality reads (quality score <20) from raw Ribo-seq and input mRNA-seq reads were performed using TrimGalore (v0.6.6) (Zhang et al., 2021). For paired-end Ribo-seq libraries, only read 1 was used in downstream analysis.

PCR duplicates were removed based on unique molecular identifiers (UMIs) using the “clumpify.sh” script from the BBMap (v38.18) (Bushnell, 2014) package. The Fastx_trimmer command from FASTX-Toolkit (v0.0.14) (Gordon and Hannon, 2010) was used to trim UMIs at the beginning and end of each read. Deduplicated reads were aligned to the IRGSP-1.0 reference genome (Kawahara et al., 2013) via STAR (v2.7.10b) (Dobin et al., 2013). Multi-mapped reads were excluded, and only uniquely mapped reads were retained for subsequent analyses. The R package DESeq2 (v1.46.0) (Love et al., 2014) was employed for sample clustering and PCA to examine reproducibility among biological replicates. The R package riboWaltz (v2.0) (Lauria et al., 2018) was used to evaluate Ribo-seq data quality, including assessment of RPF length distribution and the percentage of P-sites located within annotated transcript regions (5′ UTR, CDS, and 3′ UTR). Only reads mapping to CDSs were used to calculate TE, defined as the ratio of normalized Ribo-seq read counts to normalized RNA-seq read counts (Ingolia et al., 2009).

### Ribo-seq–associated mRNA-seq library construction and analysis

To construct Ribo-seq–associated mRNA-seq libraries, total RNA was extracted from samples using TRIzol (Thermo Fisher). Genomic DNA was removed by treatment with TURBO DNase (Thermo Fisher). DNase-treated RNA was processed via library construction steps, including mRNA isolation and fragmentation, first- and second-strand cDNA synthesis, end repair, adaptor ligation, and PCR amplification. Libraries were prepared using a Stranded mRNA-seq Lib Prep kit (ABclonal) and sequenced in 2 × 150-nt paired-end mode on the Illumina NovaSeq platform at Annoroad.

TrimGalore (v0.6.6) (Martin, 2011) was used for adaptor trimming and quality filtering of raw mRNA-seq reads. Clean reads were aligned to the IRGSP-1.0 reference genome (Kawahara et al., 2013) via STAR (v2.7.10b) (Dobin et al., 2013). Uniquely mapped BAM files were analyzed using featureCounts (v2.0.1) (Liao et al., 2014) to quantify relative mRNA expression levels.

### Immunoblot analysis

Immunoblot analysis was performed as previously described (Wang et al., 2018). Input, immunoprecipitate, and flow-through fractions from Ribo-seq were mixed with SDS loading buffer (80 mM Tris-HCl pH 6.8, 2% SDS, 10% glycerol, 0.1 M DTT, and 0.005% bromophenol blue) and heated at 95°C for 5 min. Proteins in the supernatant were subjected to immunoblotting. Membranes were probed with a primary antibody against FLAG (ABclonal, cat# AE005, IB 1:5000), followed by an HRP (Horseradish Peroxidase)-conjugated goat anti-mouse IgG secondary antibody (Thermo Fisher, cat# 31431).

### Transcriptional arrest assay for transcriptome-wide RNA decay

A transcriptional arrest assay was performed as previously described, with minor modifications (Sorenson et al., 2018; Chantarachot et al., 2020). Rice roots were harvested, cut into small disks (∼1 cm), and immersed in 10 mL ActD incubation buffer (15 mM sucrose, 1 mM PIPES pH 6.5, 1 mM KCl, 1 mM sodium citrate, and 50 μM ActD [MCE]). Initial (T_0_) samples were frozen immediately after 5 min of vacuum infiltration (∼12 psi). Remaining samples were subjected to two additional rounds of 5-min of vacuum infiltration. After three infiltration rounds, samples were incubated at 30°C with shaking at 250 rpm. Samples were collected at 15, 30, 60, and 120 min, then frozen in liquid nitrogen and ground into powder.

### Transcriptome-wide RNA decay library construction and analysis

Total RNA was extracted from powdered samples (for the RNA decay assay) using TRIzol (Thermo Fisher) and treated with TURBO DNase (Thermo Fisher). DNase-treated RNA was subjected to library construction using a Stranded mRNA-seq Lib Prep kit (ABclonal). Libraries were sequenced in 2 × 150-nt paired-end mode on the NovaSeq X Plus platform at Annoroad.

Raw RNA decay sequencing reads were processed by adaptor trimming and quality filtering using TrimGalore (v0.6.6) (Martin, 2011). Clean reads were aligned to the IRGSP-1.0 reference genome (Kawahara et al., 2013) via HISAT2 (v2.1.0) (Kim et al., 2015) with default parameters. Uniquely mapped reads were selected using “grep -E “ˆ@|NH:i:1” for downstream analyses. The featureCounts (v2.0.1) (Liao et al., 2014) package was used to obtain raw read counts for all annotated genes. The DESeq2 (v1.46.0) (Love et al., 2014) package was used for sample clustering and PCA.

Data normalization and modeling of mRNA decay were performed using the Bioconductor RNAdecay package (v1.26.0) (Reed et al., 2019). Seven stably expressed genes were used for data normalization: Os09g0535000, Os01g0658400, Os12g0507200, Os04g0667800, Os07g0597000, Os10g0320400, and Os01g0652600. To determine transcript decay rates, half-lives were calculated using the formula: *t*_1/2_ = ln(2)/α. An RNA decay heatmap was generated using ComplexHeatmap (v2.24.0) (Gu et al., 2016).

### Identification of intronless genes

The number of introns in rice genes was determined based on genome annotation (GFF3 file). Genes containing zero introns were considered intronless and used for downstream analyses.

#### Δ*G* prediction

Watson–Crick base pairs corresponding to RSS mutations were computationally removed using RNAstructure (v6.4) to simulate single-stranded RSS caused by point mutations. Perturbed structures were converted to connectivity table (CT) format using the dot2ct utility in the RNAstructure software. Subsequently, ensemble free energy (Δ*G*) values were calculated using efn2 in RNAstructure with CT files as input.

### Target-specific RNA half-life measurement

Roots of 15-day-old *OsIPS2*^*WT*^_OE, *OsIPS2*^*MUT*^_OE, and *OsIPS2*^*Re*^_OE transgenic rice plants grown under normal conditions were harvested. Root tissues were cut into small disks (∼1 cm) and immersed in 10 mL incubation buffer (15 mM sucrose, 1 mM PIPES pH 6.5, 1 mM KCl, 1 mM sodium citrate, and 1 mM cordycepin [Solarbio]). After 5 min of vacuum infiltration (∼12 psi), initial (0 min) samples were collected and immediately frozen. Remaining samples were subjected to two additional 5-min rounds of vacuum infiltration. After three infiltration rounds, samples were incubated with shaking at RT. Tissues were collected 60 min after the first vacuum release.

Total RNA was extracted using TRIzol (Thermo Fisher) and treated with TURBO DNase (Thermo Fisher). DNase-treated RNA was subjected to reverse transcription; expression levels of exogenous *OsIPS2* were quantified by RT-qPCR. The stably expressed gene Os01g0658400 served as an internal control. Primer sequences are listed in Supplemental Table 6.

### GO analysis

GO analysis was performed using AmiGO2 (Ashburner et al., 2000; Carbon et al., 2009; Aleksander et al., 2023).

### Quantification and statistical analysis

Details of statistical analyses, including tests performed, sample sizes (*n*) for each measurement, and *p* values, are provided in figure legends and figures.

## Resource availability

### Lead contact

Zhiye Wang (wangzhiye1@zju.edu.cn, College of Life Sciences, Zhejiang University).

### Materials availability

The results of DMS-MaPseq, Ribo-seq, and transcriptome-wide RNA decay assays generated in this study are provided in Supplemental Tables 1–5. The raw sequencing data from the DMS-MaPseq, Ribo-seq, and transcriptome-wide RNA decay assays have been deposited in the Genome Sequence Archive (GSA) under accession number GSA: CRA025390. Plasmids and transgenic plants generated in this study are available from the corresponding author upon reasonable request.

## Funding

This work was supported by grants from the 10.13039/501100012166National Key Research and Development Program of China (2021YFF1000402), the 10.13039/501100004731Natural Science Foundation of Zhejiang Province, China (LR24C150001), the 10.13039/501100001809National Natural Science Foundation of China (32170262), and the Fundamental Research Funds for the Central University (226-2024-00102) to Z.W.

## Acknowledgments

The authors declare no competing interests.

## Author contributions

Z.W. conceived the project and designed the experiments; Q.J., R.G., J.Y., Z.H., and G.W. performed the experiments; Q.J. conducted the data analyses, with assistance from K.L. and W.D.; and Z.W. and Q.J. wrote the manuscript with input from all authors.

## Supplemental information

Document S1. Supplemental Figures 1–13 and Supplemental note

Supplemental Table 1. Summary of processed NGS data

Supplemental Table 2. DREEM data of this study

Supplemental Table 3. RSS data of this study

Supplemental Table 4. Ribo-seq data of this study

Supplemental Table 5. Transcriptome-wide RNA decay data of this study

Supplemental Table 6. Primers and probes used in this study

Document S2. Article plus supplemental information
