# Supplementary material for: Unfolding of RNA secondary structure impairs RNA stability to fine-tune phosphate starvation responses in rice roots
Source: Plant Commun. 2025 Dec 17;7(4):101680. doi: 10.1016/j.xplc.2025.101680 (PMC13084106; doi:10.1016/j.xplc.2025.101680)
Supplement: Document S1. Supplemental Figures 1–13 and Supplemental note [file mmc1.pdf]

**Plant Communications, Volume *7***

## Supplemental information

# Unfolding of RNA secondary structure impairs RNA stability to fine-tune phosphate starvation responses in rice roots

**Qiongli Jin, Ruiren Gao, Jiakai Yao, Zhengwei Huang, Kai Liu, Guangbo Wei, Weiguo Dong, and Zhiye Wang**

**Supplemental Information**

# Unfolding of RNA secondary structure impairs RNA stability to fine-tune phosphate starvation responses in rice roots

Qiongli Jin[1], Ruiren Gao[1,2], Jiakai Yao[1,2], Zhengwei Huang[1], Kai Liu[1], Guangbo Wei[1], Weiguo Dong[1], Zhiye Wang[1,*]

**Affiliations:**

[1]State Key Laboratory of Plant Environmental Resilience, College of Life Sciences, Zhejiang University, Hangzhou, Zhejiang, 310058, China.

[2]These authors contributed equally: Ruiren Gao, Jiakai Yao.

* Correspondence: wangzhiye1@zju.edu.cn (Z.W.)

**Supplemental note**

**Generating a high-quality, deep-coverage RNA structurome from rice roots in response to N deficiency stress**

Rice roots were sampled after a 5-day N deficiency treatment (hereafter, –N), accompanied by control (normal) samples that were the same controls used for the –P samples. Three biological replicates were performed for DMS-treated samples and one biological replicate for the non-DMS-treated sample. Approximately 491–595 million clean reads were generated for each DMS-treated sample. More than 87.6% of the clean reads were uniquely mapped to the rice cv. Nipponbare reference genome IRGSP 1.0 (Supplemental Table 1).

Both clustering and PCA results exhibited high reproducibility among DMS-treated biological replicates of –N samples (Supplemental Figure 1A and 1B). The significantly reduced total N levels and dramatically increased expression of the –N response gene *OsNRT2.3* validated the success of –N treatment (Supplemental Figure 1C and 1D). Moreover, the mismatch percentages of A and C, but not G and U, were much higher in the three DMS-treated samples than in the non-DMS-treated control, showing the high signal-to-noise ratio of our data (Supplemental Figure 1E). Similar to published RNA structure data across the mRNAs (Ding *et al.*, 2014; Deng *et al.*, 2018), a 3-nt periodicity across the CDS and high DMS activity in the vicinity of the start codon were observed in our –N RNA structurome data, confirming the high reliability of the data (Supplemental Figure 4A).

Next, the RNA regions with high-conf. RNA structural information in the –N RNA structurome data were identified (see Methods). Each DMS-treated –N biological replicate obtained an average of 94,581 high-conf. RSS information windows associated with ~10,000 genes, accounting for ~42% of expressed genes (Supplemental Figure 2A). Notably, the Gini indexes of high-conf. RSS information windows exhibited high reproducibility among the three DMS-treated biological replicates (Supplemental Figure 4B). Therefore, these three biological samples were merged, yielding 187,406 high-conf. RSS information windows (corresponding to 16,148 genes) accounting for 60.80% of

expressed genes under –N conditions (Supplemental Figure 2A). Each covered gene harbored an average of 11.61 high-conf. RSS information 100-nt windows (Supplemental Figure 2A). These high-conf. RSS information windows were distributed across the whole rice genome and were associated with gene density (Supplemental Figure 4C). These windows mainly covered mRNAs, followed by small nucleolar RNAs, snRNAs, lncRNAs, rRNAs, tRNAs, and primary miRNAs (Supplemental Figure 4D).

These results confirm the high quality and deep coverage of our –N RNA structurome data.

**The features of –N induced RSS-unfolding RNA regions**

To characterize the sequence and structural features of the –N induced RSS-unfolding RNA regions, we divided –N induced RSS-unfolding windows into two subsets: –P/–N common RSS-unfolding windows (894 of total 1533, defined as –P&–N RSS-unfolding windows) and –N-unique RSS-unfolding windows (639 of total 1533) (Figure 2G). We then conduced an in-depth analysis of these two sets of RSS windows, including assessments of GC content, Gini index alterations under different nutrient conditions and GO enrichment analysis.

The results revealed that both –P&–N and –N-unique unfolding windows had a significantly lower GC content than the total windows (Supplemental Figure 5A). Furthermore, under –P and –N conditions, the Gini index of –P&–N RSS-unfolding windows was lower than that of the total RSS windows; under normal conditions, however, it was higher than that of total RSS windows, and comparable under ReP conditions (Supplemental Figure 5B). A similar trend in Gini index alteration was observed in –N-unique RSS-unfolding windows: their Gini index was lower than that of total RSS windows under –N conditions, but the opposite was true under other conditions (Supplemental Figure 5C). These results reflected dramatic RSS unfolding in both –P&–N and –N-unique RSS-unfolding RSS regions when shifting from normal to –P and/or –N conditions, demonstrating the structural flexibility of RSS in these regions.

We further performed GO enrichment analysis to investigate the functions of transcripts with –P&–N or –N-unique RSS-unfolding transcripts. Several enriched pathways were shared between –P&–N and –N-unique RSS-unfolding transcripts, such as lipid metabolism, vesicle-mediated transport, and protein transport (Supplemental Figure 5D and 5E). These shared pathways were also enriched in PSI RSS-unfolding transcripts (Supplemental Figure 6D). Of note, the amide biosynthetic process was specifically enriched in –N-unique RSS-unfolding transcripts, but not in either PSI or –P&–N RSS-unfolding transcripts (Supplemental Figure 5E). As amide biosynthesis is a critical pathway in plant N assimilation and metabolism (Liu et al., 2022), this GO result highlights the specificity of –N-unique RSS-unfolding.

Comparing the above results of –N induced RSS-unfolding analysis (Supplemental Figure 5) with the PSI RSS-unfolding analysis results (Figure 3; Supplemental Figure 6), RSS-unfolding induced by –P and –N stresses exhibited both commonalities and distinctions. On one hand, RNA regions with RSS-unfolding induced by –P and –N stresses showed low GC content, indicating that RNA structures flexibly respond to these stresses. In addition, transcripts with –P and –N-induced RSS-unfolding shared some pathways, confirming cross-talk between phosphate and nitrogen metabolism and stress responses (Paz-Ares et al., 2022). On the other hand, GO analysis also identified specific enriched pathways in PSI and –N-unique RSS-unfolding transcripts, suggesting that RSS-unfolding plays certain regulatory roles in response to different stresses.

## Supplemental Figures

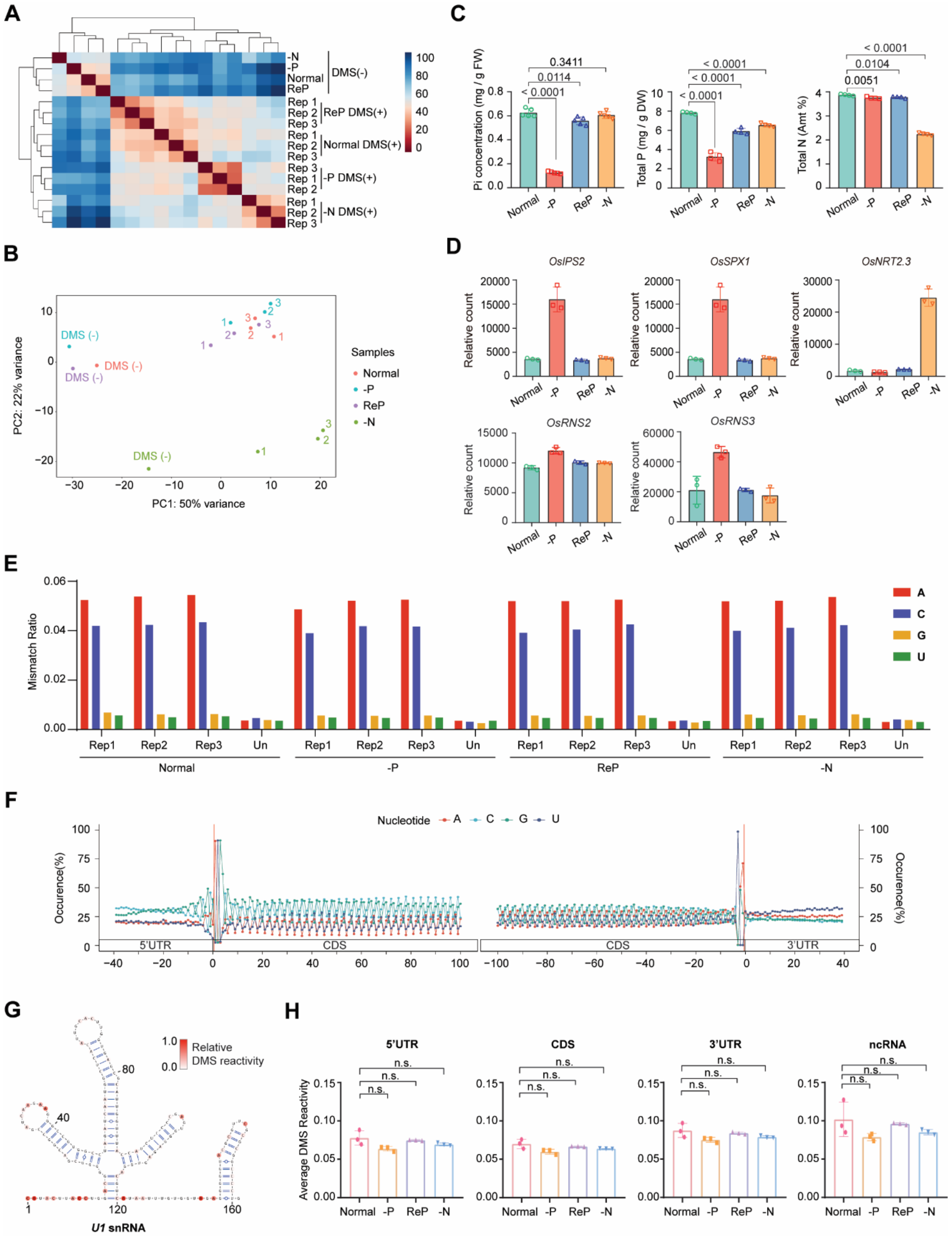


**Supplemental Figure 1. High quality and reproducibility of the *in vivo* RSS data.**

**(A and B)** Clustering **(A)** and PCA **(B)** showing the high reproducibility across three biological repeats of DMS-MaPseq from each sample.

**(C)** The Pi, total P, and total N concentrations in normal, –P, ReP, and –N samples. The *P* values were calculated using an unpaired two-tailed Student's *t*-test.

**(D)** The expression of the PSI genes *OsIPS2*, *OsSPX1*, *OsRNS2*, *OsRNS3*, and the –N-induced gene *OsNRT2.3* in normal, –P, ReP, and –N samples.

**(E)** As and Cs exhibit a higher mismatch ratio than Gs and Us in DMS-treated but not untreated (Un) samples, indicating a high signal-to-noise ratio in DMS-MaPseq.

**(F)** Average of AUGC content of all mRNAs.

**(G)** Predicted secondary structure of the *U1-5* snRNA modeled according to DMS-MaPseq data; this structure is consistent with the published crystal structure (Krummel et al., 2009). Color-coded A and C residues display different radiometric DMS signals.

**(H)** Bar diagrams revealing a lack of significant difference in the average DMS reactivity of 5′ UTRs, CDS regions, 3′ UTRs, or lncRNAs among normal, –P, ReP, and –N samples. The *P* values were calculated using an unpaired two-tailed Student's *t*-test. n.s., no significant.

–P, Pi deficiency; ReP, Pi-refeeding following Pi deficiency; –N, nitrogen deficiency.

**A**

| DMS(+) Sample name | Expressed genes | High Conf. 100nt RSS Windows | Covered Genes | Covered genes/ expressed genes | No. of RSS windows/ Covered genes |
|---|---|---|---|---|---|
| Normal Rep1 | 23,584 | 94,217 | 10,173 | 43.14% | 9.26 |
| Normal Rep2 | 24,977 | 115,275 | 11,917 | 47.71% | 9.67 |
| Normal Rep3 | 23,280 | 77,907 | 8,982 | 38.58% | 8.67 |
| **Normal merge** | **26,854** | **190,350** | **16,412** | **61.12%** | **11.60** |
| -P Rep1 | 23,685 | 95,108 | 9,869 | 41.67% | 9.64 |
| -P Rep2 | 23,714 | 93,976 | 9,980 | 42.08% | 9.42 |
| -P Rep3 | 24,398 | 105,611 | 10,995 | 45.07% | 9.61 |
| **-P merge** | **26,779** | **193,774** | **16,288** | **60.82%** | **11.90** |
| ReP Rep1 | 24,518 | 107,824 | 11,235 | 45.82% | 9.60 |
| ReP Rep2 | 24,128 | 101,692 | 10,584 | 43.87% | 9.61 |
| ReP Rep3 | 24,190 | 103,895 | 10,899 | 45.06% | 9.53 |
| **ReP merge** | **27,026** | **194,316** | **16,617** | **61.49%** | **11.69** |
| -N Rep1 | 23,944 | 100,981 | 10,518 | 43.93% | 9.60 |
| -N Rep2 | 23,687 | 92,849 | 10,014 | 42.28% | 9.27 |
| -N Rep3 | 23,612 | 89,914 | 9,992 | 42.32% | 9.00 |
| **-N merge** | **26,559** | **187,406** | **16,148** | **60.80%** | **11.61** |

**B**

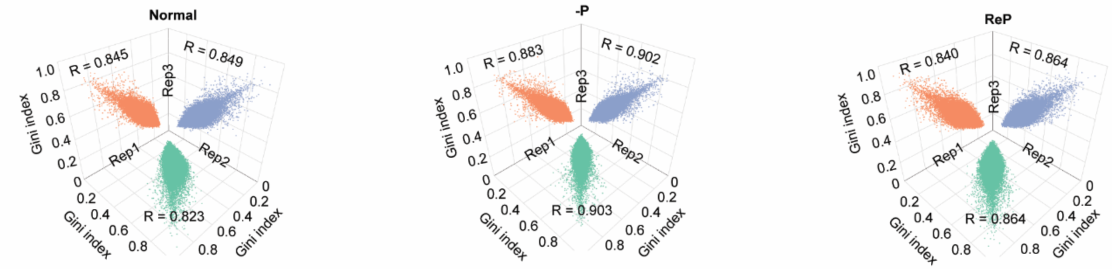


**Supplemental Figure 2. Deep-coverage of the *in vivo* RSS data.**

**(A)** Summary of the number of windows and genes with high-conf. 100-nt RSS windows in each sample, indicating that the RNA structurome data provide reliable RSS information for ~60% of all expressed genes. RSS, RNA secondary structure.

**(B)** 3D scatterplots showing the high reproducibility of Gini index values among three biological repeats in normal, –P, and ReP samples. *R* is the Pearson's correlation coefficient.

–P, Pi deficiency; ReP, Pi-refeeding following Pi deficiency; –N, nitrogen deficiency.

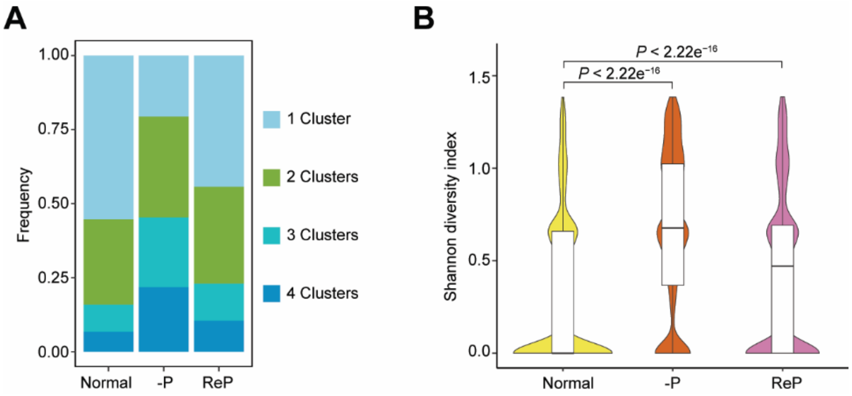


**Supplemental Figure 3. DREEM analysis showing Pi starvation–induced global RSS heterogeneity in 100-nt RNA windows with more than 25,000-read coverage.**

**(A)** DREEM analysis showing the increased prevalence of RNA structural heterogeneity in the transcriptome *in vivo*; –P induces RSS diversity compared with normal and ReP conditions. The number of clusters represents the total number of alternative RNA structures of a 100-nt RNA window *in vivo*.

**(B)** Violin plots showing the increased Shannon diversity index in –P compared with normal and ReP conditions. The *P* values were calculated using a Wilcoxon test. The midlines and box edges indicate the medians and quartiles, respectively. The whiskers extend to the farthest data point within 1.5 times the IQR from the box edges.

–P, Pi deficiency; ReP, Pi-refeeding following Pi deficiency.

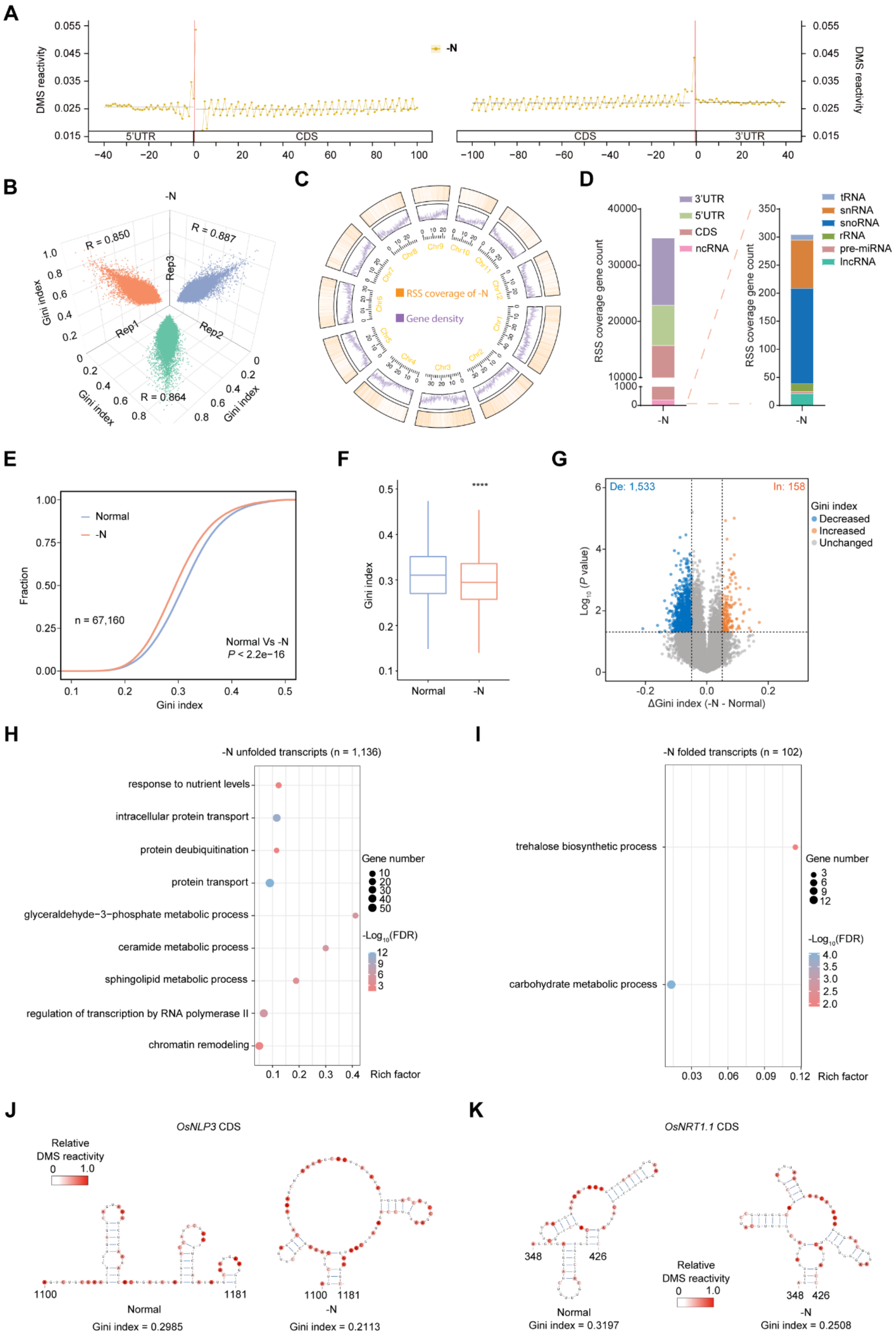
A
DMS reactivity
-N
5'UTR
CDS
3'UTR
B
-N
R = 0.850
R = 0.887
R = 0.864
Rep1
Rep2
Rep3
Gini index
C
RSS coverage of -N
Gene density
D
RSS coverage gene count
3'UTR
5'UTR
CDS
ncRNA
tRNA
snRNA
snoRNA
rRNA
pre-miRNA
lncRNA
-N
E
Normal
-N
Fraction
n = 67,160
Normal Vs -N
P < 2.2e−16
Gini index
F
****
Gini index
Normal
-N
G
De: 1,533
In: 158
Gini index
Decreased
Increased
Unchanged
Log10 (P value)
ΔGini index (-N - Normal)
H
-N unfolded transcripts (n = 1,136)
response to nutrient levels
intracellular protein transport
protein deubiquitination
protein transport
glyceraldehyde−3−phosphate metabolic process
ceramide metabolic process
sphingolipid metabolic process
regulation of transcription by RNA polymerase II
chromatin remodeling
Gene number
-Log10(FDR)
Rich factor
I
-N folded transcripts (n = 102)
trehalose biosynthetic process
carbohydrate metabolic process
Gene number
-Log10(FDR)
Rich factor
J
OsNLP3 CDS
Relative
DMS reactivity
1100
1181
Normal
Gini index = 0.2985
-N
Gini index = 0.2113
K
OsNRT1.1 CDS
348
426
Normal
Gini index = 0.3197
Relative
DMS reactivity
-N
Gini index = 0.2508

**Supplemental Figure 4. –N induces global changes in RSS.**

**(A)** Metaplots showing DMS reactivities along different segments of mRNAs under –N conditions. mRNAs were aligned by their start/stop codons (vertical red lines). Yellow ribbon indicates the standard error from three biological repeats for –N samples.

**(B)** 3D scatterplots showing the high reproducibility of Gini index values among three biological repeats in –N samples. *R* is the Pearson correlation coefficient.

**(C)** Circos plot of the gene density and high-confidence RSS information coverage under N conditions, showing the high-coverage of high-confidence RSS information genome-wide.

**(D)** Bar diagram showing high-confidence RSS regions located at various types of RNA in –N samples, mainly at CDS regions, 3′ UTRs, and 5′ UTRs of mRNAs.

**(E and F)** Cumulative curves **(E)** and box plots **(F)** showing the moderate global decrease in Gini index in –N compared to normal conditions. The *P* values were calculated using a Kolmogorov-Smirnov test in **E** and a Wilcoxon test in **F**, respectively. The midlines and box edges indicate the medians and quartiles, respectively. The whiskers extend to the farthest data point within 1.5 times the IQR from the box edges. ****, $P < 0.0001$.

**(G)** Volcano plot of altered Gini indexes of high-conf. RSS windows under –N compared with normal conditions. De, RSS windows with significantly decreased Gini indexes; In, RSS windows with significantly increased Gini indexes. The numbers of corresponding altered RSS windows are shown.

**(H and I)** GO analysis of –N unfolded **(H)** and folded **(I)** transcripts.

**(J and K)** Modeling RSS of *OsNLP3* **(J)** and *OsNRT1.1* **(K)** based on DMS-MaPseq data showing the unfolded RNA structure under –N compared with normal conditions.

–N, nitrogen deficiency.

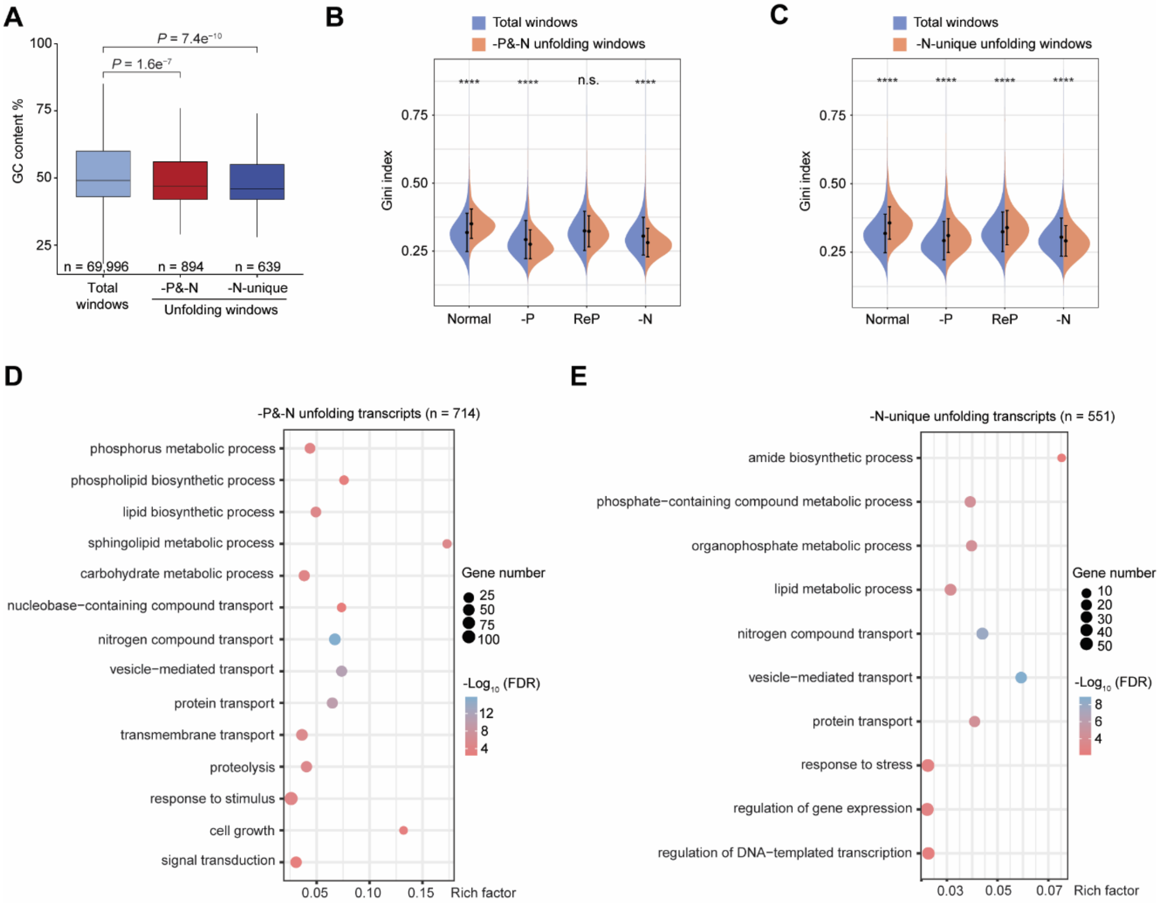


**Supplemental Figure 5. The features of –N induced RSS-unfolding regions.**

**(A)** Box plots showing significantly lower GC contents in both –P&–N and –N-unique RSS-unfolding windows than in total RSS windows. The midlines and box edges indicate the medians and quartiles, respectively. The whiskers extend to the farthest data point within 1.5 times the IQR from the box edges.

**(B and C)** Violin plots showing that the global Gini index of –P&–N (**B**) and –N-unique (**C**) RSS-unfolding windows under different nutrient conditions.

**(D and E)** GO analysis of –P&–N (**D**) and –N-unique (**E**) RSS-unfolding transcripts.

In (**A-C**), the $P$ values were calculated using a Wilcoxon test. ****, $P < 0.0001$; n.s., no significant.

–P, Pi deficiency; ReP, Pi-refeeding following Pi deficiency; –N, nitrogen deficiency.

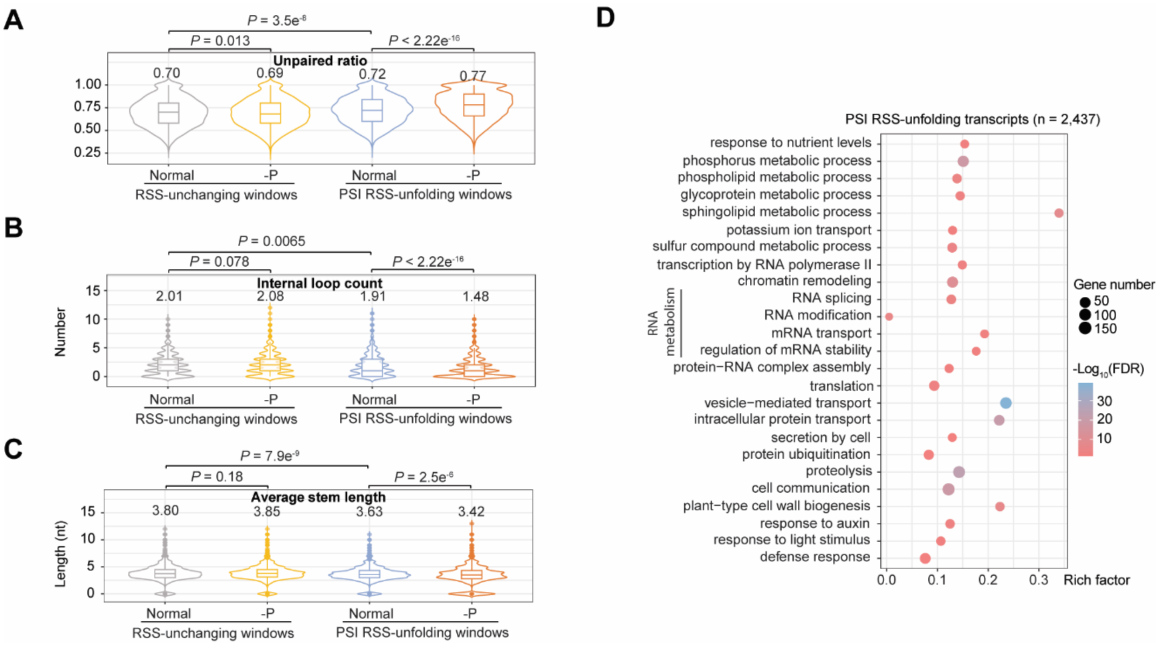


**Supplemental Figure 6. RSS features and GO analysis of PSI RSS-unfolding regions.**

**(A-C)** Diagrams showing the unpaired ratio (**A**), internal loop number (**B**), and average stem length (**C**) of RSS-unchanging windows (n = 3,543) and PSI RSS-unfolding windows (n = 3,838) under normal and –P conditions. The corresponding average values are shown. The midlines and box edges indicate the medians and quartiles, respectively. The whiskers extend to the farthest data point within 1.5 times the interquartile range (IQR) from the box edges. The *P* values were calculated using a Wilcoxon test. The average values are shown.

**(D)** GO analysis of PSI RSS-unfolding transcripts.

–P, Pi deficiency; PSI, Pi starvation induced; RSS, RNA secondary structure.

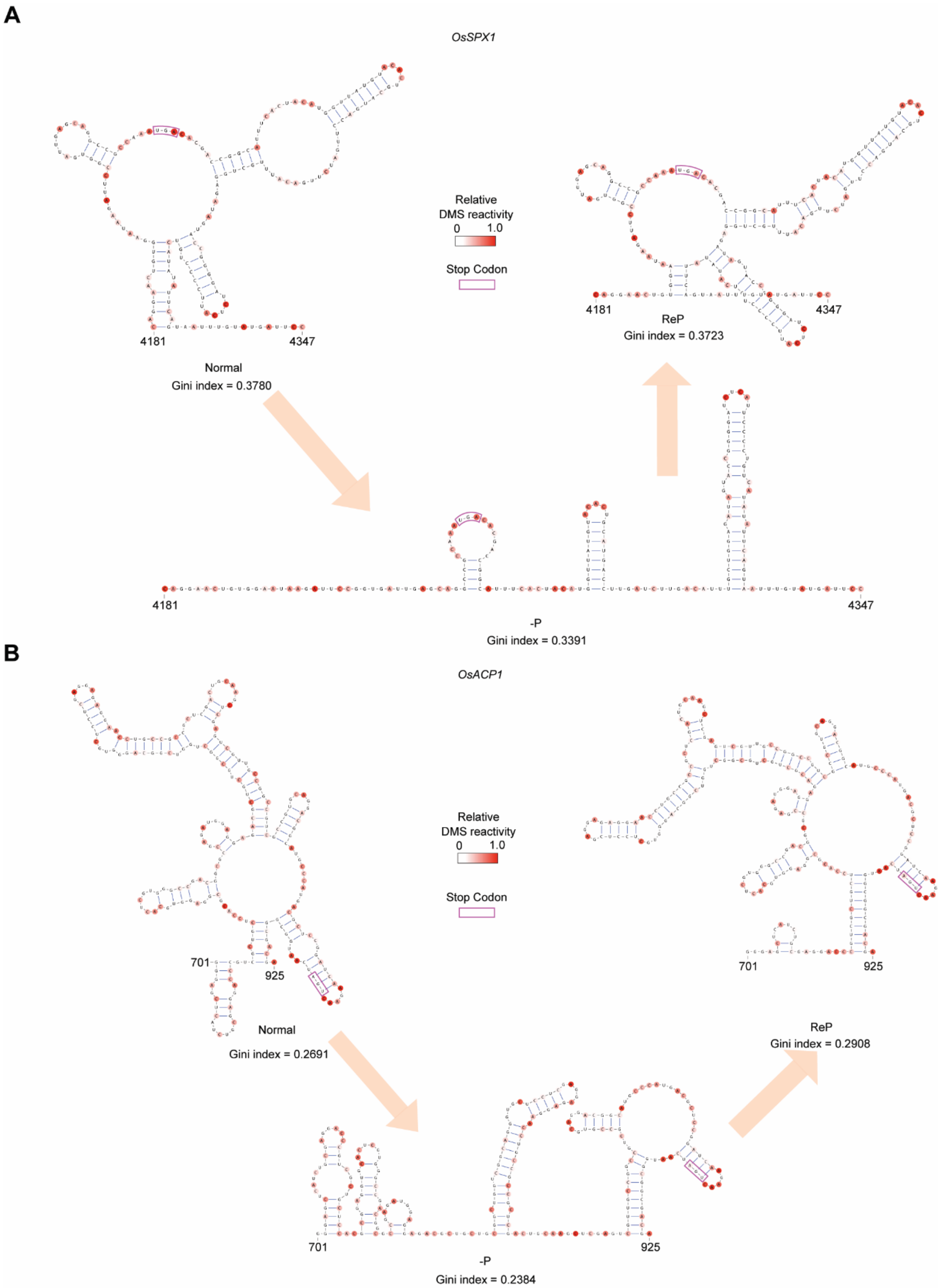


**Supplemental Figure 7. The RSS of PSR transcripts is unfolded in response to –P.**

**(A and B)** Modeling RSS of *OsSPX1* **(A)** and *OsACP1* **(B)** transcripts based on DMS-MaPseq data showing the unfolded RNA structures under –P compared with normal and ReP conditions. –P, Pi deficiency; ReP, Pi-refeeding following Pi deficiency.

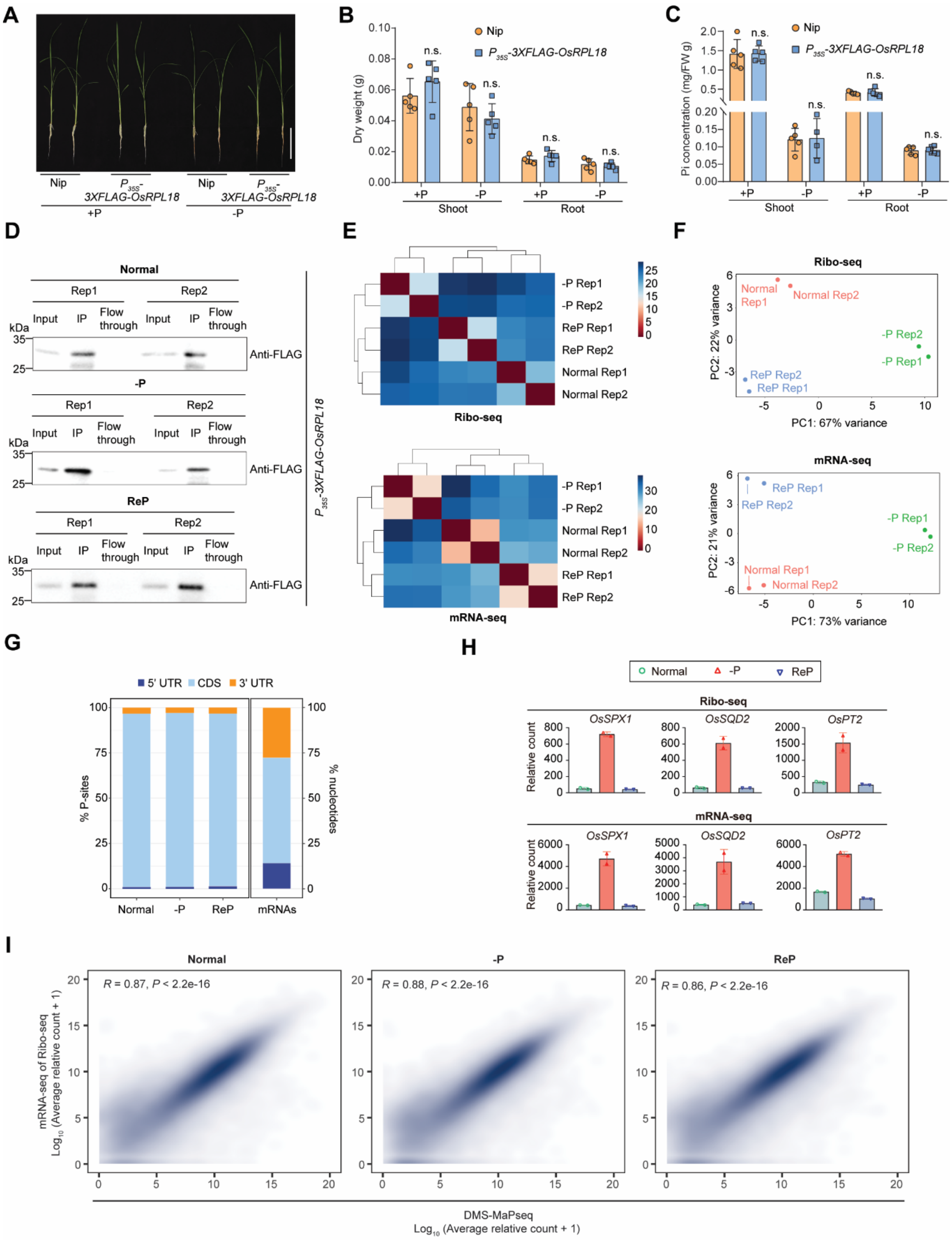

**Supplemental Figure 8. High quality of the Ribo-seq data.**

**(A)** Image showing no obvious difference in growth between Nip and *P*$_{35S}$*-3XFLAG-OsRPL18* under both normal (+P) and –P conditions. +P, normal condition. Bar = 10 cm.

**(B and C)** Bar plots showing no significant differences in either dry weight **(B)** or Pi concentration **(C)** in shoots and roots between Nip and $P_{35S}$*-3XFLAG-OsRPL18* under both normal (+P) and –P conditions. The *P* values were calculated using an unpaired two-tailed Student's *t*-test. n.s., no significant. Error bars with standard deviation were calculated from three biological replicates. +P, normal condition.

**(D)** Immunoblots showing highly efficient affinity purification of polysome–RNA complexes. IP, immunoprecipitate.

**(E and F)** Clustering **(E)** and PCA **(F)** showing high reproducibility among two biological replicates of TRAP-seq samples.

**(G)** Percentages of 5′ ends of 28-nt RPFs mapped at the 5′ UTR, CDS, and 3′ UTR in Ribo-seq data under normal, –P, and ReP conditions. The percentages of 5′ UTR, CDS, and 3′ UTR length in total mRNAs served as a control.

**(H)** Expression of the PSI genes *OsSPX1*, *OsSQD2*, and *OsPT2* based on Ribo-seq and mRNA-seq data for $P_{35S}$*-3XFLAG-OsRPL18* under normal, –P, and ReP conditions.

**(I)** Dot diagrams showing a high correlation of transcriptome data between mRNA-seq of Ribo-seq and DMS-MaPseq under normal, –P, and ReP conditions. *R* is the Pearson correlation coefficient. *P* values were calculated using the Pearson correlation test.

–P, Pi deficiency; ReP, Pi-refeeding following Pi deficiency.

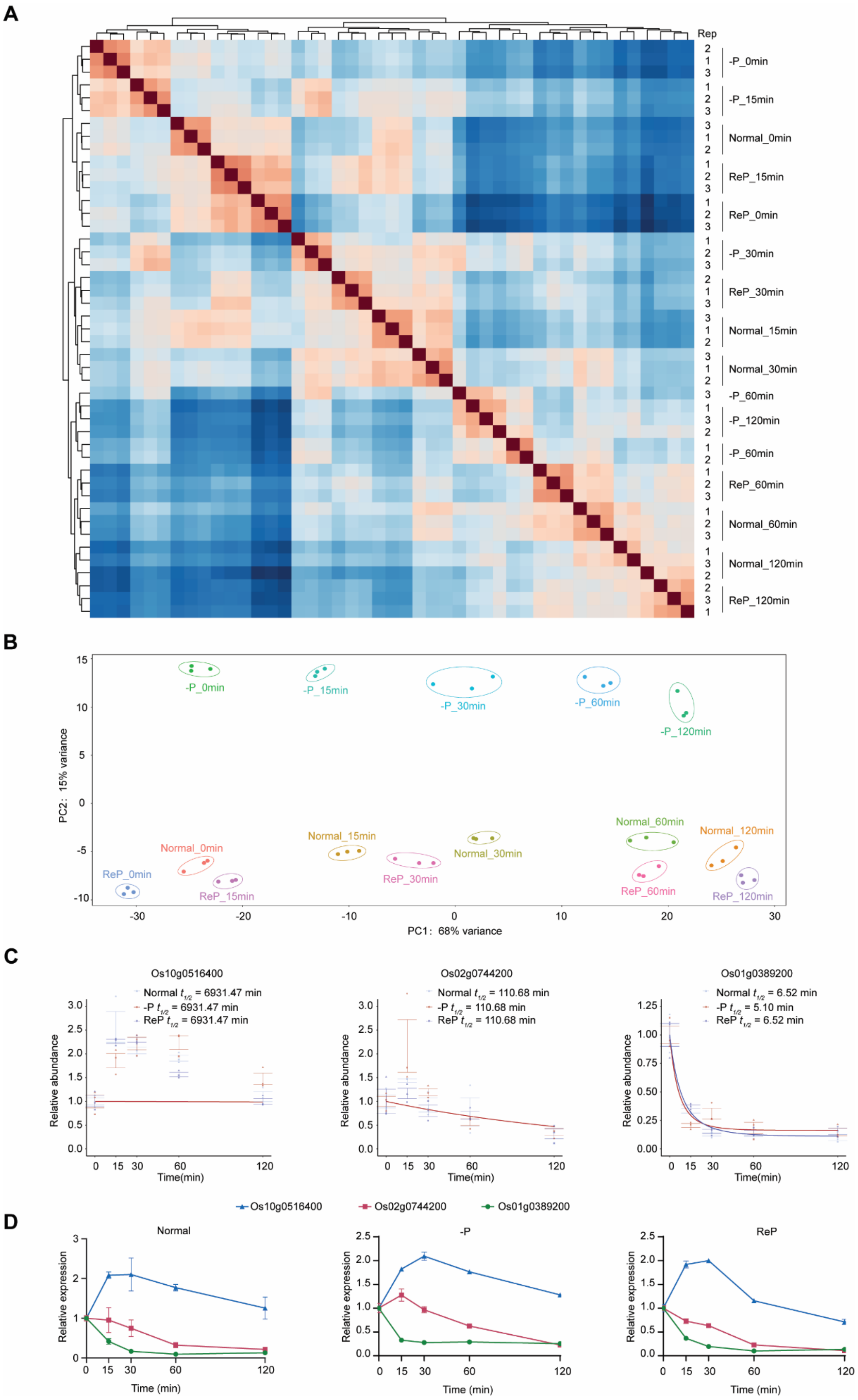
A
Rep
-P_0min
-P_15min
Normal_0min
ReP_15min
ReP_0min
-P_30min
ReP_30min
Normal_15min
Normal_30min
-P_60min
-P_120min
-P_60min
ReP_60min
Normal_60min
Normal_120min
ReP_120min
B
PC2: 15% variance
PC1: 68% variance
-P_0min
-P_15min
-P_30min
-P_60min
-P_120min
Normal_0min
ReP_0min
ReP_15min
Normal_15min
ReP_30min
Normal_30min
Normal_60min
ReP_60min
Normal_120min
ReP_120min
C
Os10g0516400
Normal $t_{1/2}$ = 6931.47 min
-P $t_{1/2}$ = 6931.47 min
ReP $t_{1/2}$ = 6931.47 min
Os02g0744200
Normal $t_{1/2}$ = 110.68 min
-P $t_{1/2}$ = 110.68 min
ReP $t_{1/2}$ = 110.68 min
Os01g0389200
Normal $t_{1/2}$ = 6.52 min
-P $t_{1/2}$ = 5.10 min
ReP $t_{1/2}$ = 6.52 min
Relative abundance
Time(min)
D
Os10g0516400
Os02g0744200
Os01g0389200
Normal
-P
ReP
Relative expression
Time (min)

**Supplemental Figure 9. High quality and fidelity of the RNA decay data.**

**(A and B)** Clustering (**A**) and PCA (**B**) showing high reproducibility among three biological replicates of the RNA decay samples.

**(C)** Profile of the decay of selected transcripts with various half-lives under normal, –P, and ReP conditions. Relative RNA abundances following the inhibition of transcription are shown, with bars indicating the means ± standard error of the mean, $n = 3$; thick lines indicate modeled values. The half-life ($t_{1/2}$) is indicated for each treatment.

**(D)** Target-specific RNA decay assay confirming the half-lives of the selected transcripts, reflecting the high fidelity of our transcriptome-wide RNA decay assay.

–P, Pi deficiency; ReP, Pi-refeeding following Pi deficiency.

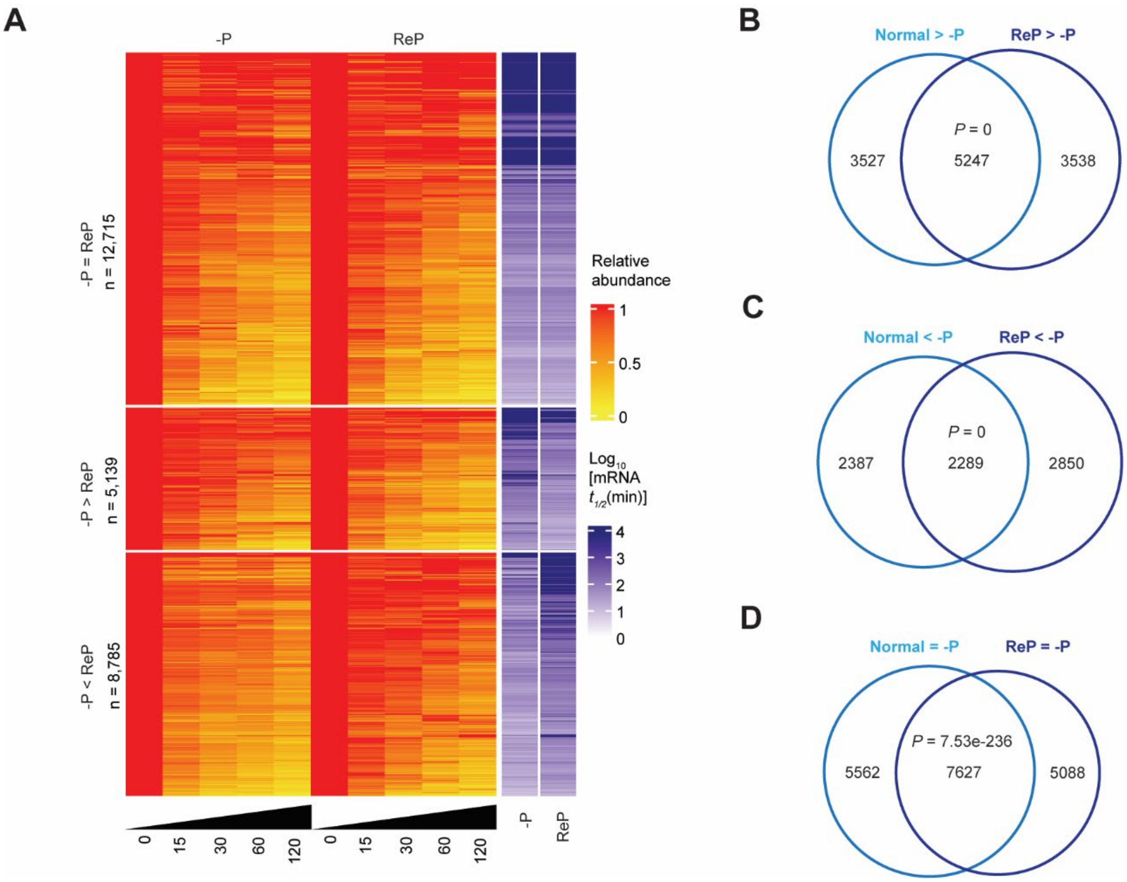


**Supplemental Figure 10. Pi starvation induces a global decrease in RNA stability.**

**(A)** Heatmap of RNA decay over 120 min in Nip under –P and ReP conditions. RNA decay dynamics were quantified by tracking the change in mean relative RNA abundance over time, with decay rates expressed as RNA half-life ($t_{1/2}$ in minutes).

**(B-D)** Venn diagrams showing the significantly overlapped transcripts with shortened (**B**), prolonged (**C**), and unchanged (**D**) half-lives between the –P vs Normal and –P vs ReP comparisons. *P* values were calculated with hypergeometric test.

–P, Pi deficiency; ReP, Pi-refeeding following Pi deficiency.

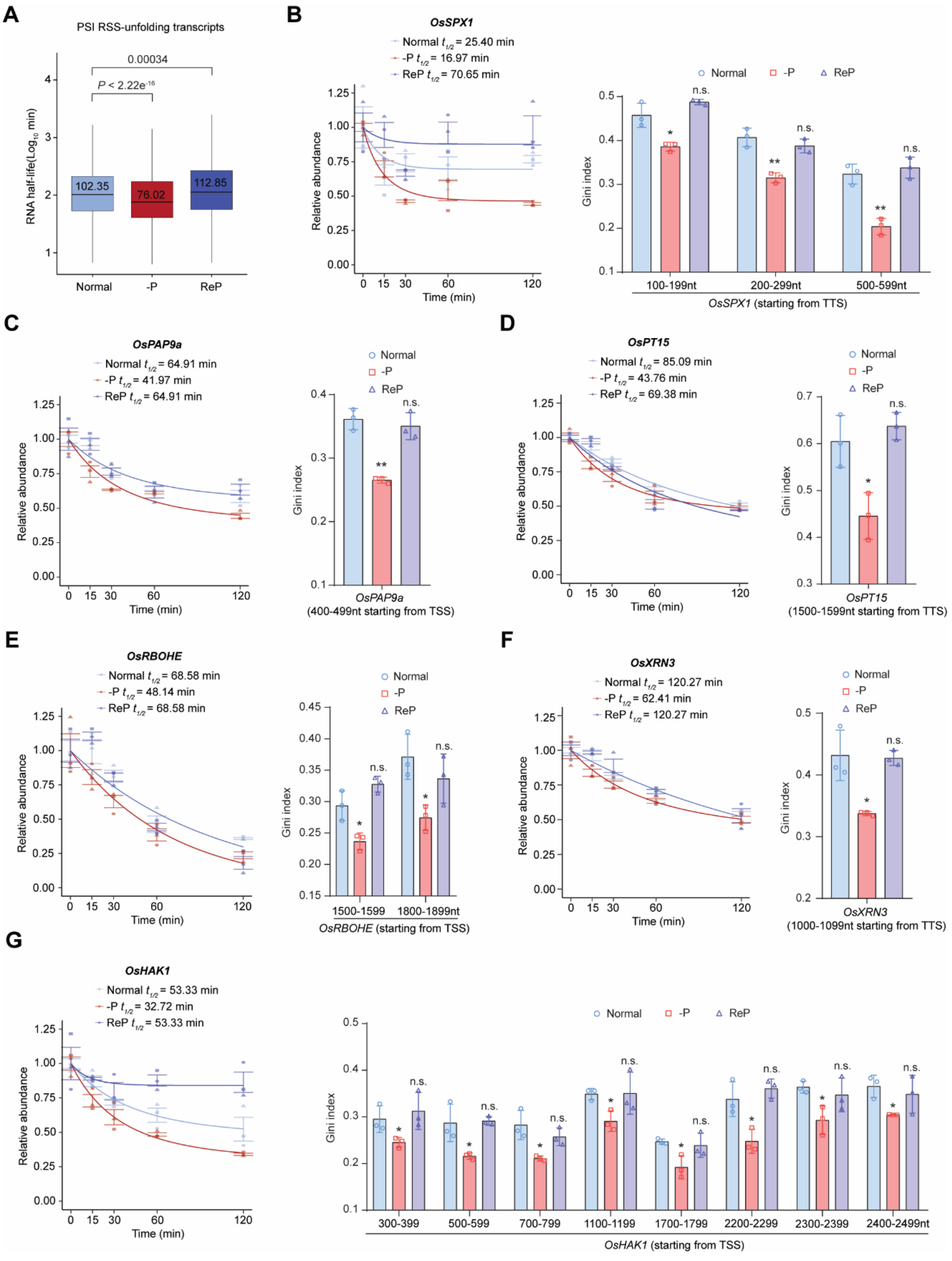


**Supplemental Figure 11. Examples of transcripts with lower RNA stability and RSS Gini indexes under –P in roots.**

**(A)** Box plots showing a global decrease in the half-lives of –P RSS-unfolded transcripts under –P, but not ReP, compared to normal conditions. The *P* values were calculated using a Wilcoxon test. The midlines and box edges indicate the medians and quartiles, respectively. The whiskers extend to the farthest data point within 1.5 times the IQR from the box edges.

**(B-G)** Both the half-lives and Gini indexes of *OsSPX1* **(B)**, *OsPAP9a* **(C)**, *OsPT15* **(D)**, *OsRBOHE* **(E)**, *OsXRN3* **(F)**, and *OsHAK1* **(G)** decreased in response to –P and were rescued after ReP. In the half-life profile, relative RNA abundances following the inhibition of transcription are shown, with bars indicating the means ± standard error of the mean, $n = 3$; thick lines indicate modeled values. The half-life ($t_{1/2}$) is indicated for each treatment. In the Gini index bar plots, the *P* values were calculated using an unpaired two-tailed Student's *t*-test. **, $P < 0.01$; *, $P < 0.05$; n.s., no significant.

–P, Pi deficiency; ReP, Pi-refeeding following Pi deficiency.

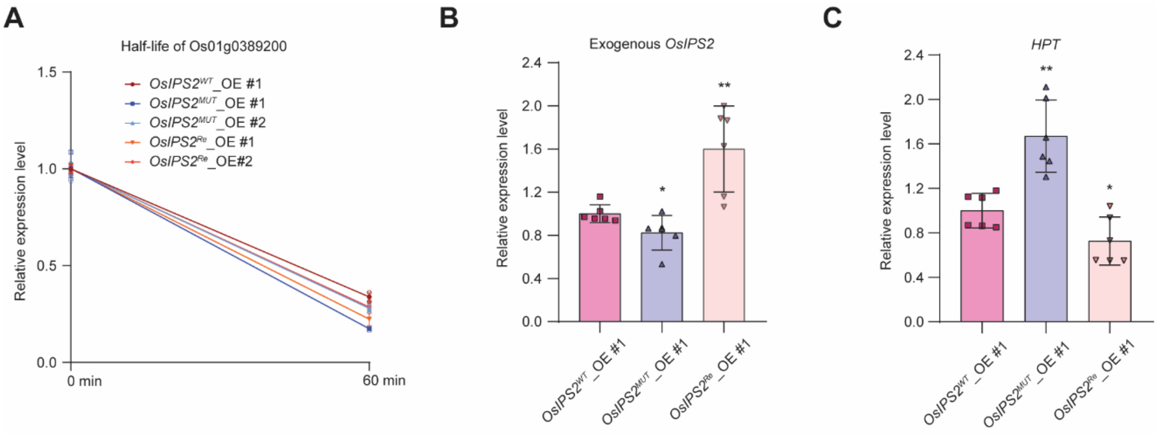


**Supplemental Figure 12. The transcription was successfully stopped by chemical treatment and the expression levels of exogenous *OsIPS2* and *HPT* transcripts in Nip and transgenic plants.**

**(A)** Target-specific RNA decay of the half-lives of control unstable transcripts of Os01g0389200 in the *OsIPS2*$^{WT}$_OE, *OsIPS2*$^{MUT}$_OE, and *OsIPS2*$^{Re}$_OE transgenic lines. The relative expression levels of the transcripts were normalized to those at the 0-min time point, where the ratio was arbitrarily set to 1, with standard deviation calculated from three biological repeats.

**(B and C)** Bar plots showing the expression levels of exogenous *OsIPS2* (**B**) and *HPT* transcripts (**C**) in roots of Nip, *OsIPS2*$^{WT}$_OE #1, *OsIPS2*$^{MUT}$_OE #1 and *OsIPS2*$^{Re}$_OE #1 plants. The relative expression levels of genes were normalized to those of *OsIPS2*$^{WT}$_OE #1, where the ratio was arbitrarily set to 1 with SD calculated from two biological repeats with three technical repeats. *$P < 0.05$; **$P < 0.01$, as determined using an unpaired two-tailed Student's *t*-test.

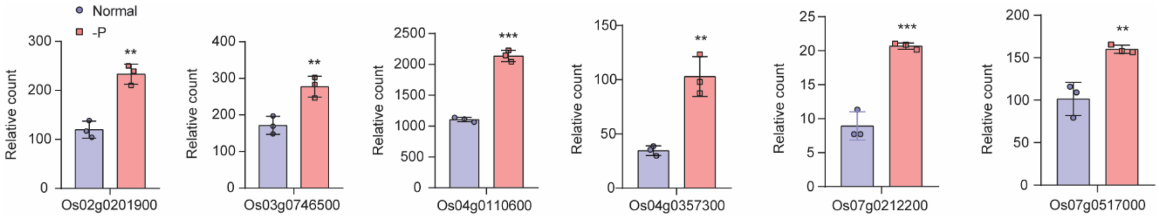


**Supplemental Figure 13. Several RNA helicases and RBP genes are induced by –P.**

The expression levels of Pi starvation induced RNA helicases and RNA binding protein-encoding genes under normal and –P conditions. ** $P < 0.01$ *** $P < 0.001$; unpaired two-tailed Student's *t*-test. –P, Pi deficiency.
