# Supplementary material for: Unfolding of RNA secondary structure impairs RNA stability to fine-tune phosphate starvation responses in rice roots
Source: Plant Commun. 2025 Dec 17;7(4):101680. doi: 10.1016/j.xplc.2025.101680 (PMC13084106; doi:10.1016/j.xplc.2025.101680)
Supplement: Document S2. Article plus supplemental information [file mmc8.pdf]

Plant Communications
Research article

CellPress
Partner Journal

Unfolding of RNA secondary structure impairs RNA stability to fine-tune phosphate starvation responses in rice roots

Qiongli Jin[1], Ruiren Gao[1,2], Jiakai Yao[1,2], Zhengwei Huang[1], Kai Liu[1], Guangbo Wei[1], Weiguo Dong[1] and Zhiye Wang[1,*]

[1]State Key Laboratory of Plant Environmental Resilience, College of Life Sciences, Zhejiang University, Hangzhou, Zhejiang 310058, China

[2]These authors contributed equally

***Correspondence: Zhiye Wang (wangzhiye1@zju.edu.cn)**

https://doi.org/10.1016/j.xplc.2025.101680

ABSTRACT

The availability of essential macronutrients, such as inorganic phosphate (Pi) and nitrogen, limits global crop production. *In vivo* RNA secondary structure (RSS) regulates nearly all steps of the RNA life cycle and is dynamic under stress conditions. However, the roles of RSS in plant responses to nutrient deficiency remain unclear. Here, we used dimethyl sulfate mutational profiling with sequencing (DMS-MaPseq) to generate high-quality, deep-coverage *in vivo* RSS profiles of rice (*Oryza sativa*) roots in response to Pi deficiency (–P) or nitrogen deficiency (–N) stress. –P increased global RSS diversity and triggered RSS unfolding in thousands of transcripts. By comparing –P and –N RSS profiles, we identified –P-specific RSS-unfolding regions in rice roots. These regions, characterized by low GC content, were enriched within the coding sequences of many Pi starvation response transcripts. Ribosome profiling suggested that –P-specific RSS unfolding was not associated with translational regulation. In contrast, transcriptome-wide RNA decay assays under normal, –P, and Pi-refeeding conditions revealed global regulation of RNA stability in response to –P in rice roots; decreased RNA half-life was linked to RSS unfolding. Transcriptome analysis and analyses of transgenic rice plants with altered RSS demonstrated that –P-specific RSS unfolding lowers RNA stability, thus fine-tuning the accumulation of Pi starvation response transcripts and Pi homeostasis. This study systemically elucidates the dynamic roles and regulatory functions of RSS in the Pi starvation response in rice roots. Our findings underscore the importance of RSS in modulating nutrient-deficient stress responses.

Key words: *in vivo* RNA secondary structure, nutrient-deficient stress, RNA stability, translation, rice

Jin Q., Gao R., Yao J., Huang Z., Liu K., Wei G., Dong W., and Wang Z. (2026). Unfolding of RNA secondary structure impairs RNA stability to fine-tune phosphate starvation responses in rice roots. Plant Comm. **7**, 101680.

INTRODUCTION

Nutrient deficiency, particularly of phosphorus (P) and nitrogen (N), restricts global crop production (Schachtman and Shin, 2007; Bailey-Serres et al., 2019). For example, the macronutrient P is essential for plant growth and reproduction (Lambers, 2022). Plants absorb inorganic P (Pi), also termed orthophosphate, from the soil; however, due to its immobility, low solubility, or generally low abundance, limited P availability constrains crop yields on over 70% of the world's arable land (Vance, 2001; López-Arredondo et al., 2014; Cong et al., 2020). In addition to nutrient uptake, roots sense environmental nutrient levels and respond to nutrient limitation (Gutiérrez-Alanis et al., 2018; Bouain et al., 2019). Indeed, plants have evolved numerous developmental and metabolic responses to cope with Pi limitation (Paz-Ares et al., 2022; Yang et al., 2024). The phosphate starvation response (PSR) includes remodeling of root system architecture (Péret et al., 2014; Abel, 2017), PSR signaling centered on PHR (PHOSPHATE STARVATION RESPONSE) transcription factors and SPX-domain-containing proteins (Puga et al., 2014, 2017; Wang et al., 2014; Jung et al., 2018), Pi absorption and translocation (Versaw and Garcia, 2017; Yamaji et al., 2017), and organic Pi metabolism and reuse (Paz-Ares et al., 2022). These PSR processes are regulated at multiple levels, such as transcription (Rubio et al., 2001; Zhou et al., 2008; Dai et al., 2012; Guo et al., 2015; Ruan et al., 2018), post-transcriptional gene silencing mediated by phosphate starvation–induced *miRNA399–OsIPS1/2* (Chiou et al.,

 © 2025 The Author(s). Published by Elsevier Inc. on behalf of CAS Center for Excellence in Molecular Plant Sciences, Chinese Academy of Sciences, Chinese Society for Plant Biology. This is an open access article under the CC BY license (http://creativecommons.org/licenses/by/4.0/).

2006; Franco-Zorrilla et al., 2007; Lin et al., 2008; Pant et al., 2008), protein phosphorylation (Chen et al., 2015b; Yang et al., 2020c), and ubiquitination (Pan et al., 2019). Investigation of additional layers of PSR regulation may provide target pathways for crop improvement, including enhanced low-Pi tolerance and improved Pi utilization.

RNA transcripts form complex secondary structures via base pairing. RNA secondary structure (RSS) in living cells is dynamic and dependent on cellular context (Xu et al., 2022). Several methods that couple small-molecule–mediated RNA modification with next- or third-generation sequencing have been developed to profile RSS *in vivo* at single-nucleotide resolution (Ding et al., 2014; Rouskin et al., 2014; Spitale et al., 2015; Weng et al., 2020; Yang et al., 2022a). Among these approaches, dimethyl sulfate (DMS) mutational profiling with sequencing (DMS-MaPseq) is widely used in studies of eukaryotic species due to its single-nucleotide resolution and high signal-to-noise ratio (Zubradt et al., 2017; Wang et al., 2019; Xu et al., 2022). In this method, DMS modifies the Watson–Crick face of unpaired adenosine (A) and cytosine (C) to N^1-methyladenosine (m^1A) and N^3-methylcytidine (m^3C) in RNA. These modifications are subsequently converted to cDNA mutations using thermostable group II intron reverse transcriptase (TGIRT) (Zubradt et al., 2017). A single RNA sequence can adopt various structures within the cell (Xu et al., 2022). Algorithms such as detection of RNA folding ensembles using expectation maximization (DREEM) (Tomezsko et al., 2020) have been developed to identify coexisting alternative RSS of the same transcripts based on DMS-MaPseq data. However, the diversity of transcriptome-wide *in vivo* RSS in plants has not yet been investigated. We recently optimized DMS-MaPseq for *in vivo* profiling of RSS in rice (*Oryza sativa*), facilitating the study of RNA structure–mediated regulation in crops (Jin et al., 2022).

RSS plays important regulatory roles in prokaryotic and eukaryotic cells (Wang et al., 2021; Zhu et al., 2021; Xu et al., 2022; Zhang and Ding, 2025). Studies of plant RNA structure have examined *in vivo* target-specific RNA structures or the transcriptome-wide RNA structurome, demonstrating that RSS regulates multiple molecular and biological processes, including miRNA biogenesis (Wang et al., 2018; Li et al., 2024; Yan et al., 2024), cleavage (Yang et al., 2020a), splicing (Ding et al., 2014; Deng et al., 2018; Liu et al., 2021), translation efficiency (Ding et al., 2014; Deng et al., 2018; Yang et al., 2020b, 2021; Xiang et al., 2023), alternative polyadenylation (Liu et al., 2021), stability (Yang et al., 2020b; Wu et al., 2024; Zhang et al., 2024), and phase separation (Zhang et al., 2019). RSS is reprogrammed and exerts regulatory effects through RNA decay or translational regulation in plants in response to environmental stresses, such as heat (Su et al., 2018) and salinity (Tack et al., 2020). RSS also participates in Pi homeostasis in rice. Rice *PHOSPHATE 1;2* (*OsPHO1;2*) encodes a Pi exporter and has an associated *cis*-natural antisense transcript known as *cis-NAT*$_{PHO1;2}$ (Jabnoune et al., 2013). Reis and colleagues investigated the *in vitro* RSS of the *OsPHO1;2*–*cis-NAT*$_{PHO1;2}$ interaction and proposed a model in which *cis*-NAT-mediated translational regulation of the cognate sense mRNA occurs via changes in RSS (Reis et al., 2021). Additionally, we previously identified the *in vivo* RSS of the 5′ untranslated region (UTR) of *OsPHO2* mRNA—encoding a key regulator of Pi homeostasis—using target-specific DMS-MaP-seq. We discovered a target-adjacent nucleotide motif–like unfolded RNA structure downstream of the OsmiR399 target site (Jin et al., 2022). However, transcriptome-wide *in vivo* RSS profiling and the functions of RSS in response to nutrient-deficient stress remain elusive.

The root is the major plant tissue for nutrient absorption and transfer, as well as the primary tissue for sensing and responding to nutritional fluctuations in the soil (Gutiérrez-Alanis et al., 2018; Bouain et al., 2019). Here, we explored the transcriptome-wide dynamics of *in vivo* RSS and the roles of these structures in regulating responses to nutrient deficiency stress in rice roots. We obtained high-quality, deep-coverage *in vivo* RNA structurome data from rice roots in response to Pi deficiency (–P) and nitrogen deficiency (–N) using optimized DMS-MaPseq methods. Through integrated analysis of *in vivo* RNA structurome data with genome-wide ribosome profiling (Ribo-seq) and RNA half-life assays, we determined that –P-specifically induced (PSI) RSS unfolding is associated with RNA decay. Analysis of RSS-mutated transgenic rice plants further demonstrated that PSI alterations in RSS modulate RNA stability and Pi homeostasis.

RESULTS

High-quality, deep-coverage *in vivo* RSS landscape of rice roots in response to Pi starvation

To investigate RNA structural dynamics and their potential regulatory roles in the PSR of rice, we profiled the transcriptome-wide RNA structurome in roots under normal growth and Pi starvation conditions using our optimized DMS-MaPseq method (Figure 1A) (Jin et al., 2022). We sampled the roots of rice seedlings after a 5-day –P treatment and after a 5-day –P treatment followed by 2 days of Pi refeeding (hereafter, ReP), along with control samples grown under normal conditions (hereafter, normal) (Figure 1A).

For each sample, we prepared three biological replicates for DMS-treated samples (+DMS) and one reference control without DMS treatment (–DMS) for DMS-MaPseq. We obtained approximately 454–769 million clean reads for each DMS-treated replicate. More than 85% of the clean reads were uniquely mapped to the rice Nipponbare (Nip) reference genome (Ensembl Plants *Oryza sativa Japonica Group*, IRGSP 1.0) (Supplemental Table 1). Clustering analysis and principal component analysis (PCA) demonstrated high reproducibility among the three biological replicates for each sample (Supplemental Figure 1A and 1B). Changes in Pi concentration and PSR marker gene expression levels validated the effects of the –P treatments (Supplemental Figure 1C and 1D). Next, we examined the enrichment of mismatched nucleotides to assess DMS-MaPseq data quality, given that DMS methylates A and C in RNA; DMS lesions are decoded as mismatches in cDNA by the reverse transcriptase TGIRT (Zubradt et al., 2017). Compared with control samples, the mismatch percentages of A and C, but not guanosine (G) or uridine (U), were dramatically increased in all DMS-treated samples, indicating high signal-to-noise ratios (Supplemental Figure 1E).

Consistent with published *in vivo* RSS of mRNAs (Ding et al., 2014; Deng et al., 2018), we detected three-nucleotide

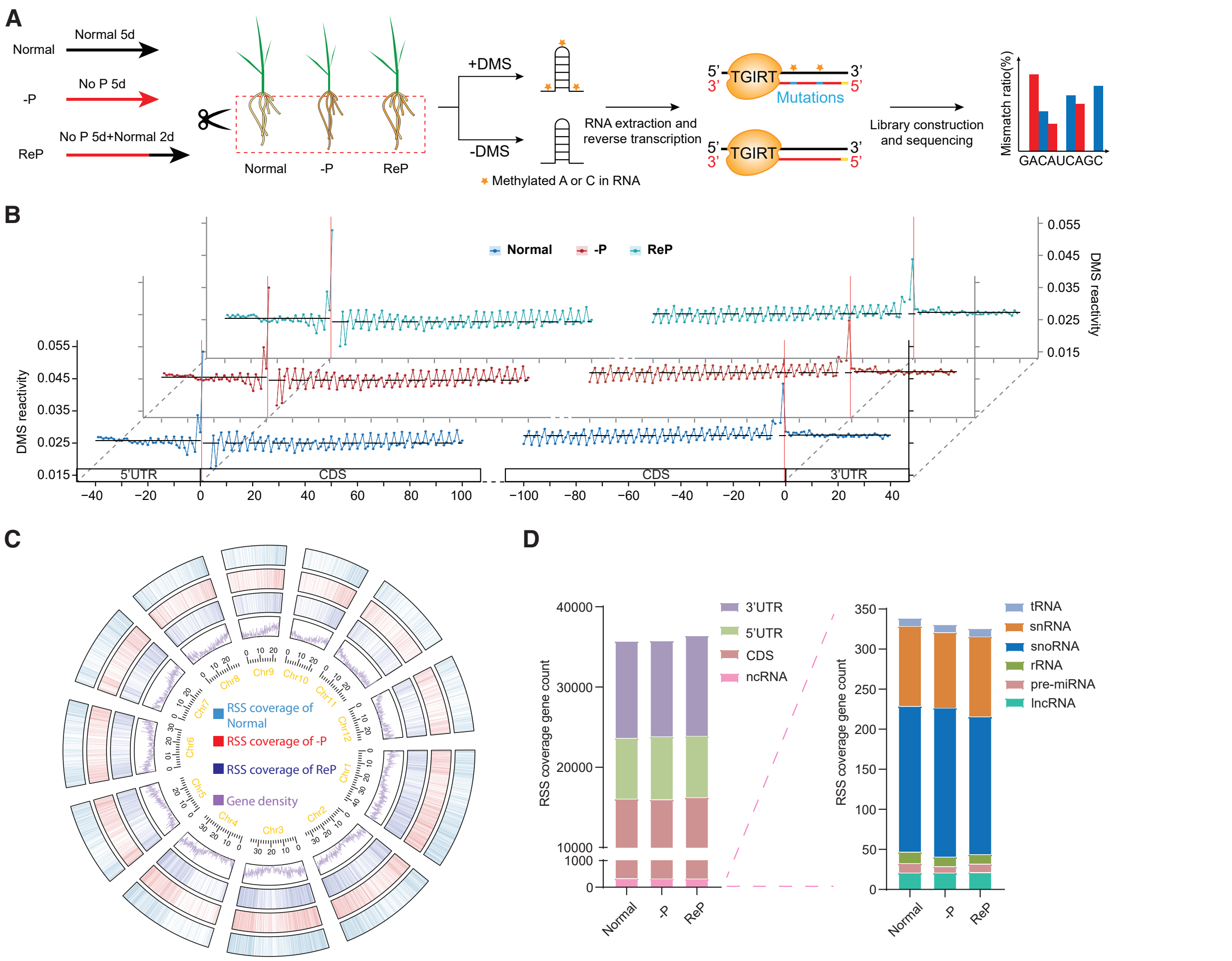

Figure 1. High-quality, deep-coverage *in vivo* RSS landscape in rice roots in response to Pi starvation

(A) Schematic overview of transcriptome-wide *in vivo* RSS profiling using the DMS-MaPseq method.

(B) Metaplots showing DMS reactivities across various mRNA regions under normal, –P, and ReP conditions. mRNAs were aligned according to start and stop codons (vertical red lines). Blue, orange, and cyan ribbons indicate standard errors from three biological replicates for normal, –P, and ReP samples, respectively.

(C) Circos plot showing gene density and high-confidence RSS information coverage under normal, –P, and ReP conditions, demonstrating extensive genome-wide coverage of high-confidence RSS information.

(D) Bar charts showing the distribution of high-confidence RSS regions across various RNA types, predominantly within CDS regions, 3′ UTRs, and 5′ UTRs of mRNAs. –P, Pi deficiency; ReP, Pi-refeeding following Pi deficiency; DMS, dimethyl sulfate.

periodicity across the coding sequence (CDS) and elevated DMS activity in the vicinity of the start codon within our RSS data, confirming high reliability (Figure 1B; Supplemental Figure 1F). Moreover, the RSS of *U1* small nuclear RNA (snRNA) predicted from DMS mutation signals was consistent with the published crystal structure (Krummel et al., 2009), further confirming the reliability of the DMS-MaPseq data (Supplemental Figure 1G). Next, we compared global RNA structural features and DMS activity among samples under normal and stress conditions. Intriguingly, –P did not alter global RNA structural features or average DMS activity across mRNAs at the genome-wide level (Figure 1B; Supplemental Figure 1H).

We then identified RNA regions with high-confidence (high-conf.) RSS information at the transcriptome-wide level. We divided each transcript into 100-nt windows (see methods). Windows harboring average mismatch counts of >20 for both A and C combined were regarded as high-conf. RSS information regions (see methods). Each DMS-treated biological replicate produced approximately 100,000 high-conf. RSS information windows associated with approximately 10,000 genes, representing ~44% of the expressed genes (Supplemental Figure 2A). Because RSS information was highly reproducible among biological replicates (Supplemental Figure 1A, 1B, and 2B), we merged the three biological replicates to increase coverage. Notably, each merged sample contained approximately 190,000 high-conf. RSS information windows associated with ~16,000 genes, constituting at least 60% of the expressed genes (Figure 1C; Supplemental Figure 2A). On average, each covered gene contained 11 high-conf. 100-nt RSS information windows (Supplemental Figure 2A). Regions with higher gene density encompassed more high-conf. RSS windows, confirming the reliability of the RSS data (Figure 1C). Additionally, high-conf. RSS information covered various RNA types, including mRNAs, non-coding RNAs, pre-miRNAs,

ribosomal RNAs, transfer RNAs, snRNAs, and small nucleolar RNAs (Figure 1D). The majority of high-conf. RSS windows (99%) corresponded to mRNAs: 44% covered CDS regions, 34% covered 3′ UTRs, and 21% covered 5′ UTRs (Figure 1D). Notably, low coverage of non-coding RNAs was observed, which may be attributed to low expression levels or incomplete genome annotation (Figure 1D). These results indicate that the rice root RNA structurome data are highly reliable and provide deep coverage.

–P increases RSS diversity in rice roots

Although –P did not alter ensemble DMS activity (Supplemental Figure 1H), we investigated whether –P stress induces changes in RSS. A single RNA sequence can adopt various structures within the cell (Xu et al., 2022). We first assessed whether –P stress affects *in vivo* RSS diversity using the RSS clustering algorithm DREEM (Tomezsko et al., 2020), which identifies alternative RNA conformations of the same RNA sequence based on DMS-MaPseq data (Tomezsko et al., 2020). Because DREEM analysis requires super-high sequencing depth (at least 50,000 reads per 100-nt window), and given the high quality and reproducibility of the DMS-MaPseq data among the three biological replicates (Supplemental Figures 1A, 1B, and 2B), we merged the three biological replicates for this analysis. Additionally, we divided each transcript into 100-nt windows and retained those with at least 50,000-read coverage. Approximately 5,575–7,354 100-nt windows (corresponding to 504–642 genes) met this threshold in each merged sample. We selected 4,110 windows common to all three growth conditions for DREEM analysis.

DREEM showed that more than 76% of the tested windows could be clustered into two to four alternative RNA structures, suggesting widespread RSS heterogeneity *in vivo* (Figure 2A; Supplemental Table 2). Remarkably, –P treatment increased the ratio of windows with four alternative RNA structures but decreased the ratio of windows with one or two alternative RNA structures compared with samples under normal and ReP conditions (Figure 2A). We obtained similar results when analyzing windows with at least 25,000-read coverage: 11,564 100-nt windows (937 genes) (Supplemental Figure 3A). These findings indicate that –P increases RSS heterogeneity in rice roots.

To quantify RSS diversity, we utilized the Shannon diversity index to simultaneously measure the number and ratio of alternative RSS (Finkel et al., 2019) (see methods); a higher Shannon diversity index indicated greater RSS diversity. Consistent with the RSS clustering results (Figure 2A; Supplemental Figure 3A), –P increased the global Shannon diversity index, whereas ReP partially restored this index toward levels observed under normal conditions (Figure 2B; Supplemental Table 2). This pattern was also observed when analyzing windows with at least 25,000 read coverage across all three growth conditions (Supplemental Figure 3B). Our results constitute further evidence that –P enhances *in vivo* RSS diversity in rice roots.

In summary, DREEM analysis demonstrated that –P stress induces increased RSS diversity in rice roots, suggesting the occurrence of global RSS reprogramming in response to –P stress.

–P stress triggers transcriptome-wide RSS unfolding in rice roots

To examine changes in RSS in response to Pi starvation in greater detail, we used the Gini index to indicate RNA conformation: a high Gini index reflects a highly folded RSS, whereas a low Gini index denotes unfolded RSS (Zubradt et al., 2017; Zhang et al., 2024). Gini indices of high-conf. RSS information windows were highly reproducible among the three biological replicates (Supplemental Figure 2B). Compared with normal growth conditions, the global Gini index was significantly decreased under –P but was restored to normal levels under ReP (Figures 2C and 2D). These results reveal transcriptome-wide RSS unfolding and increased RNA accessibility in response to –P.

We then identified RSS regions that were significantly altered by –P treatment. Consistent with the transcriptome-wide analysis, 5, 638 windows (corresponding to 3,123 genes) showed significant decreases in Gini index upon –P treatment compared with normal conditions ($p < 0.05$, ΔGini index [–P – normal] ≤ -0.05); only 17 windows (corresponding to 16 genes) showed significant increases ($p < 0.05$, ΔGini index [–P – normal] $\geq$ 0.05) (Figure 2E; Supplemental Table 3). In contrast, relative to normal conditions, the Gini indices of only 298 windows (corresponding to 255 genes) significantly decreased ($p < 0.05$, ΔGini index [ReP – normal] ≤ -0.05), whereas those of 741 windows (corresponding to 639 genes) significantly increased ($p < 0.05$, ΔGini index [ReP – normal] $\geq$ 0.05) under ReP conditions (Figure 2F; Supplemental Table 3). These results indicate that –P triggers global RSS unfolding in rice roots.

Our results demonstrate that –P induces global changes in RSS, predominantly unfolding, suggesting that RSS plays important roles in regulating the PSR in rice roots.

Identification of –P-specific RSS-unfolding transcripts

To further identify –P-specific RSS-unfolding regions, we profiled transcriptome-wide RSS in Nip plants subjected to 5 days of treatment with low levels of nitrogen (–N), another macronutrient essential for plant growth (Liu et al., 2022b). We used three DMS-treated (+DMS) biological replicates and one DMS-untreated (–DMS) control, yielding high-quality, deep-coverage RNA structurome data under –N conditions (Supplemental Figures 1 and 4A–4D; Supplemental note). Similar to –P stress, –N stress predominantly triggered RSS unfolding in a large number of transcripts (Supplemental Figure 4E and 4F). The Gini indices of 1,533 windows (corresponding to 1,136 genes) significantly decreased ($p < 0.05$, ΔGini index [–N – normal] ≤ -0.05), whereas those of only 158 windows (corresponding to 102 genes) significantly increased ($p < 0.05$, ΔGini index [–N – normal] $\geq$ 0.05) under –N relative to normal conditions (Supplemental Figure 4G; Supplemental Table 3). These –N-induced RSS-unfolding transcripts were enriched in Gene Ontology (GO) terms related to responses to nutrient levels, protein deubiquitination, protein transport, glyceraldehyde-3-phosphate metabolism, sphingolipid metabolism, and transcription (Supplemental Figure 4H). In contrast, –N-induced RSS-folding transcripts were associated with carbohydrate metabolism and trehalose metabolism during

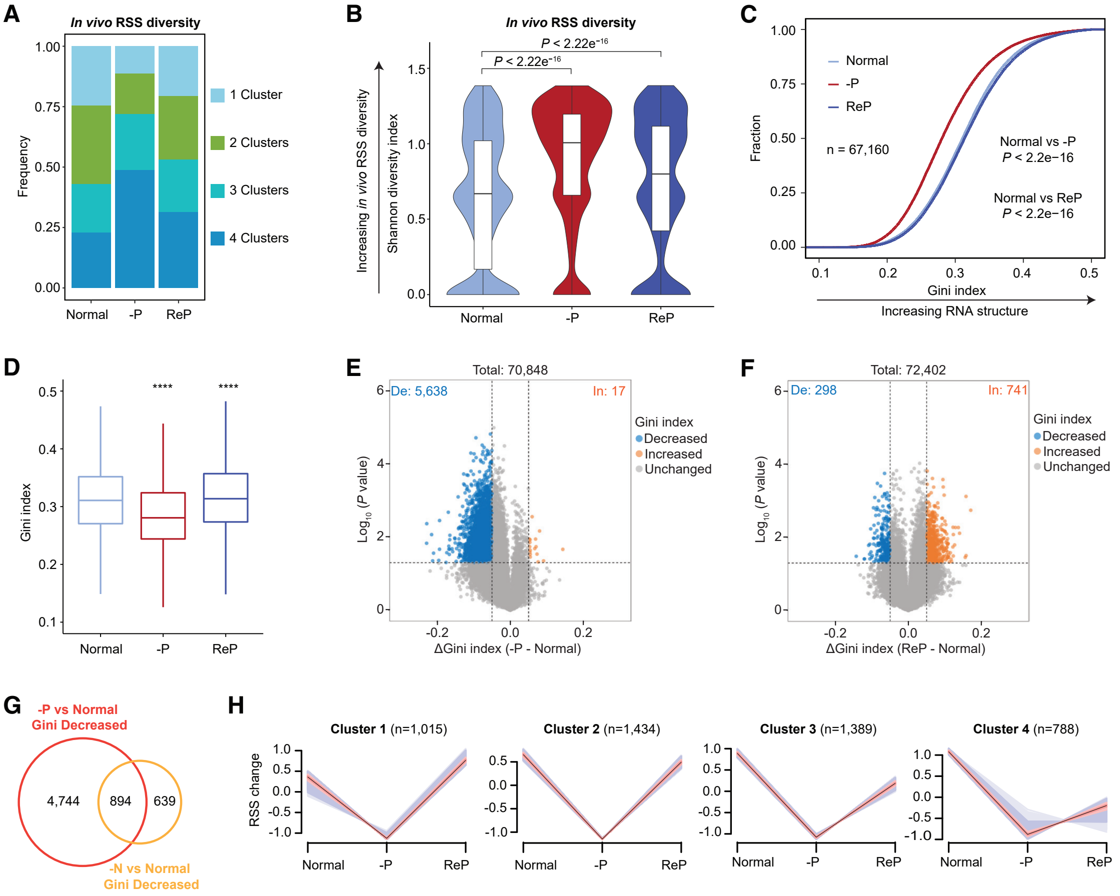

Figure 2. Pi starvation induces global RSS diversity and RSS unfolding

(A) DREEM analysis showing increased RNA structural heterogeneity *in vivo* and elevated RSS diversity under –P compared with normal and ReP conditions. The DREEM algorithm groups sequencing reads derived from each structure into distinct clusters, each representing a unique RNA secondary structure. The number of clusters reflects the total number of alternative RNA structures within a 100-nt RNA window *in vivo*.

(B) Violin plots showing increased Shannon diversity indices under –P compared with normal and ReP conditions. The *p* values were calculated using the Wilcoxon test.

(C and D) Cumulative distribution curves (C) and boxplots (D) showing global decreases in Gini index under –P compared with normal and ReP conditions. *p* values were calculated using the Kolmogorov–Smirnov test in (C) and the Wilcoxon test in (D). ****, $p < 0.0001$.

(E and F) Volcano plots showing altered Gini indices of high-conf. RSS windows under –P (E) and ReP (F) compared with normal conditions. De, RSS windows with significantly decreased Gini indices; In, RSS windows with significantly increased Gini indices. Numbers of altered RSS windows are shown.

(G) Venn diagram showing overlap between –P- and –N-induced RSS windows with decreased Gini indices.

(H) Identification of clusters of windows exhibiting –P-induced decreases in Gini index using the R package Mfuzz. In (B) and (D), midlines and box edges indicate medians and quartiles, respectively. Whiskers extend to the farthest data point within 1.5 times the interquartile range (IQR) from box edges. –P, Pi deficiency; ReP, Pi-refeeding following Pi deficiency; –N, nitrogen deficiency.

stress responses (Supplemental Figure 4I). Notably, transcripts of several genes involved in N sensing and signaling exhibited unfolding in response to –N stress, including the nitrate signaling gene NIN-like protein 3 (*OsNLP3*) (Liu et al., 2022a; Zhang et al., 2022) and the nitrate transporter gene NITRATE TRANSPORTER *1.1* (*OsNRT1.1*) (Fan et al., 2017), indicating a potential regulatory role for RSS in –N responses (Supplemental Figure 4J and 4K).

Next, we compared –P- and –N-induced RSS-unfolding windows. More than half of the –N-induced RSS-unfolding windows (58.3%) were also induced by –P (15.8%) (Figure 2G). We subtracted these overlapping windows from the –P-induced RSS-unfolding set to obtain candidate RSS-unfolding windows specifically induced by –P. We applied the Mfuzz algorithm (fuzzy c-means), based on Euclidean distance, to the Gini index of each window across normal, –P, and ReP conditions to cluster these candidate –P-specific RSS-unfolding windows into four groups (Figure 2H). RSS windows in clusters 1 to 3 were unfolded under –P but exhibited approximately normal folding after ReP (Figure 2H). We defined these RSS windows as putative PSI RSS-unfolding RNA regions, comprising 3,838 RSS windows that correspond to 2,437 genes (Supplemental Table 3).

Features of PSI RSS-unfolding RNA regions

We characterized the sequence and structural features of the PSI RSS-unfolding RNA regions. Most transcripts in these regions

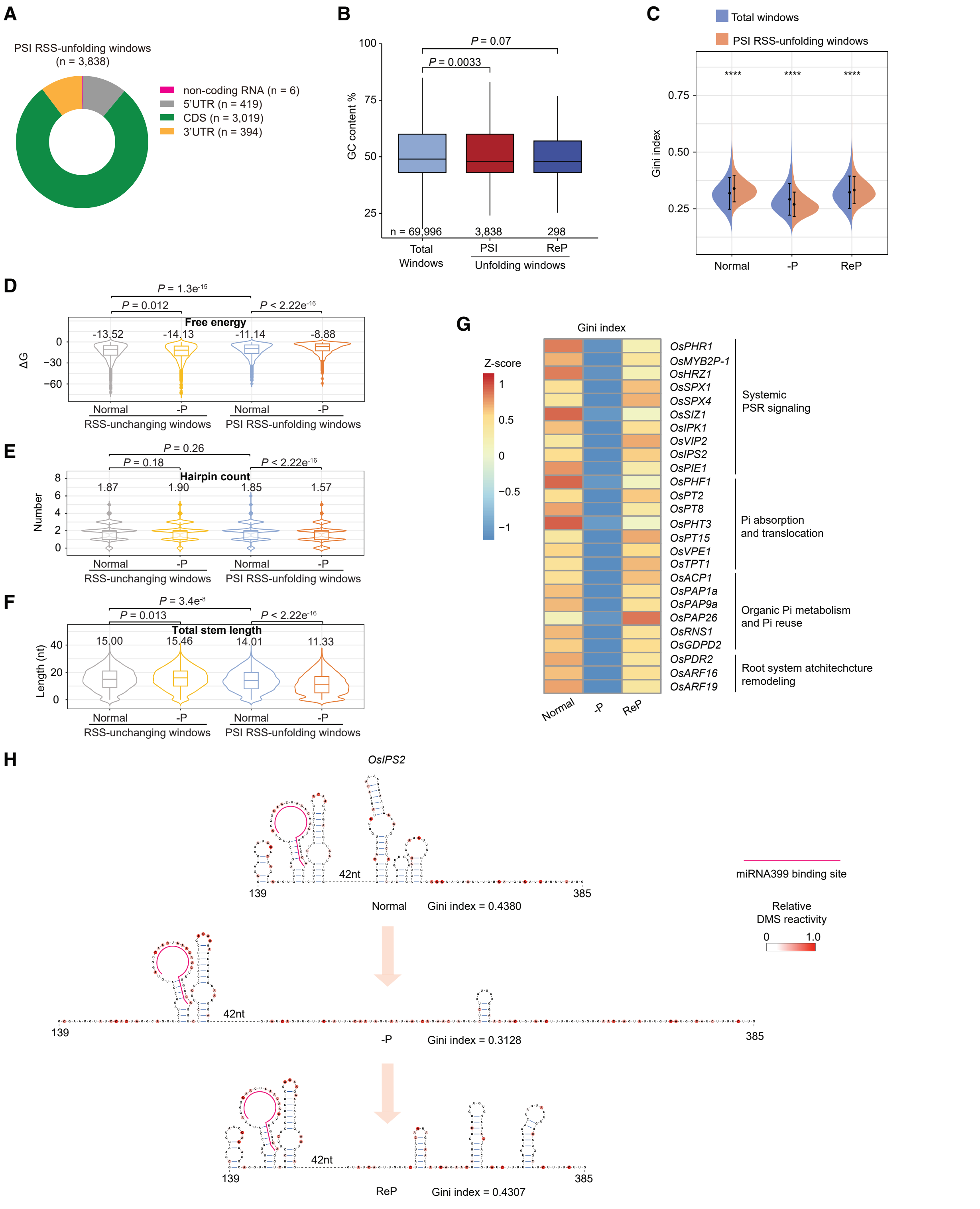

Figure 3. Characteristics of PSI RSS-unfolding regions

(A) Percentages of PSI RSS-unfolding windows at various transcript locations. PSI RSS-unfolding windows were predominantly located in CDS regions, followed by 5′ UTRs, 3′ UTRs, and non-coding RNAs. Numbers of the indicated transcripts are shown.

(B) Boxplots showing significantly lower GC content in PSI RSS-unfolding windows than in total RSS windows and unfolding RSS windows under ReP conditions.

(C) Violin plots showing that the global Gini index of PSI RSS-unfolding windows was higher than that of total RSS windows under normal and ReP conditions but lower under –P conditions. *p* values were calculated using the Wilcoxon test. ****, $p < 0.0001$.

(legend continued on next page)

originated from protein-coding genes, and PSI RSS-altered regions were predominantly located within CDSs (79% of the total) (Figure 3A). Only six RSS windows were located in non-coding RNAs, including *OsIPS2* (Hou et al., 2005) (Figure 3A). Because RNA regions with low GC content are generally more flexible due to the lower stability of A-U base pairs relative to G-C base pairs (Chan et al., 2009), we assessed GC content in PSI RSS-unfolding regions. Indeed, we found that PSI RSS-unfolding windows, but not ReP-mediated RSS-unfolding windows, exhibited significantly lower GC content compared with genome-wide RSS windows (Figure 3B). These results indicate that PSI RSS-unfolding regions with low GC content form flexible RNA structures.

We subsequently compared the RNA structures of total and PSI RSS-unfolding windows under normal, –P, and ReP conditions. As expected, PSI RSS-unfolding windows showed significantly decreased RSS folding, as indicated by a lower Gini index, than total RSS windows under –P stress (Figure 3C). In contrast, under normal and ReP conditions, PSI RSS-unfolding windows displayed significantly higher RSS folding than total RSS windows (Figure 3C). These findings confirm pronounced changes in RSS within PSI RSS-unfolding RNA regions between normal and –P conditions, revealing the structural flexibility of these regions. Intriguingly, this feature and lower GC content were observed in both the –P/–N common and –N-unique RSS-unfolding regions, highlighting shared characteristics among RSS-unfolding regions induced by –P and –N (Supplemental Figures 5A–5C; Supplemental note).

Next, we assessed putative RSS features responsive to –P. We selected the top 5% of RSS windows with the lowest ΔGini index (|–P –Normal|) values to serve as control RSS-unchanging windows (n = 3543). We integrated our DMS-MaPseq data into the RSS feature analysis (see methods). The results showed that, in PSI RSS-unfolding windows, free energy and unpaired ratio values increased; hairpin count, internal loop count, and total and average stem lengths decreased from normal to –P conditions (Figures 3D–3F; Supplemental Figures 6A–6C). In contrast, these RSS indices in RSS-unchanging windows were comparable between the two conditions (Figures 3D–3F; Supplemental Figures 6A–6C), validating the fidelity of the RSS analysis. To further evaluate RSS features of PSI RSS-unfolding regions, we compared RSS indices of PSI RSS-unfolding windows with those of RSS-unchanging windows under normal conditions. Relative to RSS-unchanging windows, PSI RSS-unfolding windows exhibited higher free energy and unpaired ratio values, whereas hairpin and internal loop counts were similar (Figures 3D and 3E; Supplemental Figure 6A and 6B). This pattern suggests that PSI RSS-unfolding windows contain shorter stems. Consistent with observations thus far, both the total and average stem lengths of PSI RSS-unfolding windows were shorter than those of control RSS-unchanging windows (Figure 3F; Supplemental Figure 6C). In summary, these results demonstrate that PSI RSS-unfolding predominantly occurs in regions with shorter stems and higher free energy.

To explore the roles of transcripts exhibiting PSI RSS unfolding, we performed GO enrichment analysis. PSI RSS-unfolding transcripts were enriched in multiple pathways, including responses to nutrient levels, P metabolism, transcription, RNA metabolism, translation, proteolysis, cell communication, and defense responses (Supplemental Figure 6D), suggesting that PSI RSS unfolding broadly affects cellular processes. Notably, transcripts of many known PSR genes were unfolded under –P conditions (Figure 3G). These included genes involved in systemic PSR signaling, such as *OsPHR1* -) (Zhou et al., 2008), *OsSPX1* (*Syg1/Pho81/XPR1*) (Wang et al., 2014), *OsVIP2* (Zhu et al., 2019), and *OsIPS2* (Hou et al., 2005); genes involved in Pi absorption and transfer, such as *OsPHF1* (Chen et al., 2011), *OsPT2* (Liu et al., 2010), and *OsPT8* (Jia et al., 2011); genes involved in organic Pi metabolism and Pi reuse, such as *OsACP1* (Deng et al., 2022) and *OsPAP26* (Gao et al., 2017); and genes involved in root system architecture remodeling, such as *OsPDR2* (Ticconi et al., 2009) and *OsARF16* (Shen et al., 2013) (Figure 3G). To validate –P-induced RSS unfolding in these key Pi homeostasis and PSR signaling genes, we modeled *in vivo* RSS based on DMS activity data. Indeed, all three examined RNA regions exhibited –P-mediated RSS unfolding, consistent with their DMS reactivity profiles (Figure 3H; Supplemental Figure 7). These results indicate an additional layer of PSR regulation at the RSS level.

Global PSI RSS unfolding is not related to translational regulation in roots

Ribosomes unwind mRNA secondary structures during translation, resulting in a global decline in RNA structure within highly translated transcripts, as observed in zebrafish (Beaudoin et al., 2018). This observation prompted us to investigate whether PSI RSS unfolding was coupled with increased translation. We performed translating ribosome affinity purification (TRAP)-based ribosome profiling (Ribo-seq) of rice roots under normal, –P, and ReP conditions (Figure 4A). We generated transgenic plants harboring constitutive 35S promoter–driven ribosomal protein L18 (RPL18) fused with 3×FLAG tag (*P_{35S}-3×FLAG-OsRPL18*) in the Nip background. These transgenic plants did not display obvious differences in morphology or PSR relative to wild-type (WT) plants (Supplemental Figures 8A–8C, 8H, and 8I). We sampled roots of *P_{35S}-3×FLAG-OsRPL18* plants and performed affinity purification of ribosome-RNA complexes using anti-FLAG antibodies. We then digested the samples with RNase I to obtain ribosome-protected fragments (RPFs) and performed high-throughput sequencing

(D–F) Plots showing free energy (D), hairpin count (E), and total stem length (F) of RSS-unchanging windows (n = 3543) and PSI RSS-unfolding windows (n = 3838) under normal and –P conditions. Corresponding average values are shown. ΔG, Gibbs free energy change.

(G) Heat map showing changes in Gini indices of PSI RSS-unfolding transcripts with known involvement in the PSR and Pi homeostasis. PSR, Pi starvation response.

(H) RSS modeling of *OsIPS2* based on DMS-MaPseq data, showing unfolded RNA structure under –P compared with normal and ReP conditions. In (B and D–F), p values were calculated using the Wilcoxon test. Midlines and box edges indicate medians and quartiles, respectively. Whiskers extend to the farthest data point within 1.5 times the IQR from box edges. –P, Pi deficiency; ReP, Pi-refeeding following Pi deficiency; PSI, Pi starvation induced; RSS, RNA secondary structure.

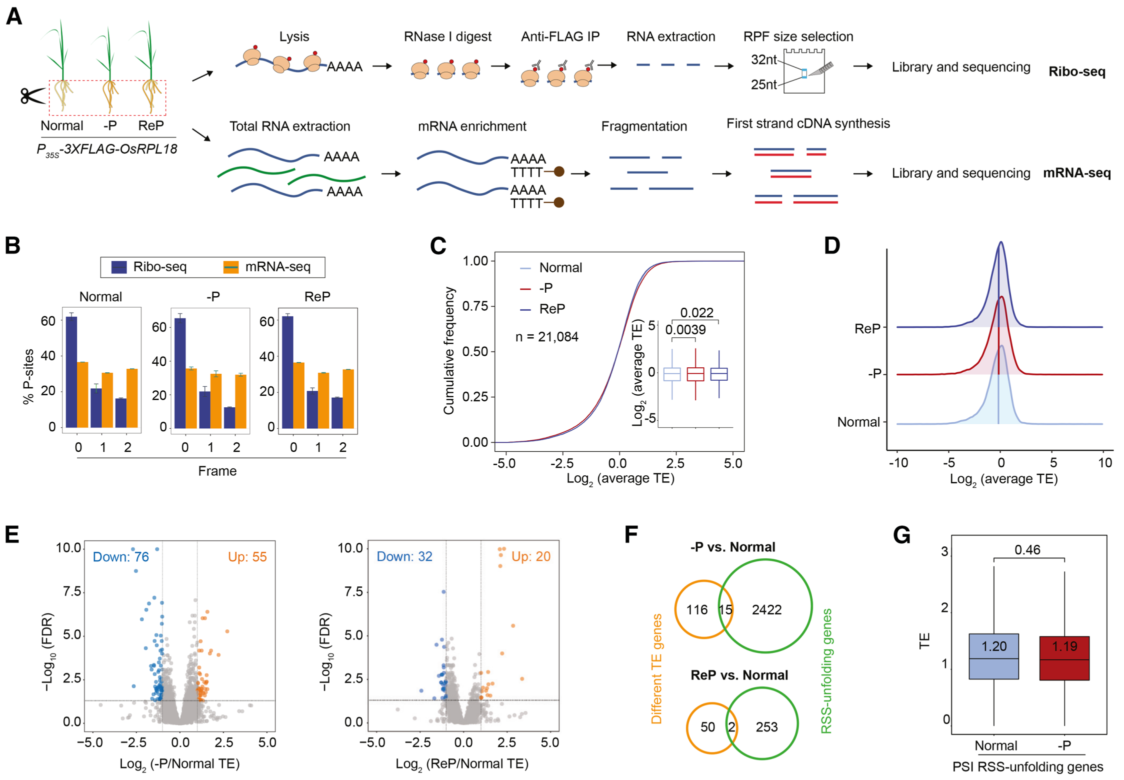

Figure 4. –P-specific RSS unfolding is not related to translational regulation

(A) Schematic overview of the workflow for TRAP-based Ribo-seq.

(B) Bar plots showing pronounced 3-nt periodicity at the 5′ ends of 28-nt RPFs (dark blue), but not at the 5′ ends of mRNA-seq reads (orange), across reading frames in normal, –P, and ReP samples. Error bars represent standard errors from biological replicates ($n = 2$). “0”, “1”, and “2” denote first, second, and third nucleotide positions, respectively, at the 5′ ends of the sequences.

(C) Cumulative curves and boxplots showing comparable global TE under normal, –P, and ReP conditions. p values for boxplots were calculated using the Wilcoxon test.

(D) Ridgeline plots showing comparable global TE distributions under normal, –P, and ReP conditions.

(E) Volcano plots showing individual transcripts with altered TE under –P and ReP relative to normal conditions. Down, transcripts with significantly decreased TE; Up, transcripts with significantly increased TE. Numbers of transcripts with altered TE are shown.

(F) Venn diagrams showing that few genes exhibited both altered TE and RSS unfolding under –P or ReP conditions.

(G) Boxplots showing comparable global TE of genes with PSI RSS unfolding under normal and –P conditions. p values were calculated using the Wilcoxon test. –P, Pi deficiency; ReP, Pi-refeeding following Pi deficiency; TE, translational efficiency.

(Figure 4A; Supplemental Figure 8D; Supplemental Table 1). Corresponding strand-specific mRNA-seq was conducted in parallel (Figure 4A; Supplemental Table 1).

Clustering analysis and PCA demonstrated high reproducibility among biological replicates of each sample (Supplemental Figure 8E and 8F). Consistent with published data obtained by conventional ultracentrifugation-based Ribo-seq (Hsu et al., 2016), more than 95% of RPFs mapped to protein-coding regions in the Ribo-seq data (Supplemental Figure 8G). Additionally, more than 61% of the 5′ termini of 28-nt RPFs mapped to the first nucleotide of individual codons, exhibiting strong 3-nt periodicity (Figure 4B). These results support the high quality of the Ribo-seq data.

We calculated the translational efficiency (TE) of each mRNA, defined as the ratio of Ribo-seq reads to mRNA-seq reads for each protein-coding open reading frame (Bazin et al., 2017). –P did not induce obvious global changes in TE (Figures 4C and 4D; Supplemental Table 4). Relative to normal conditions, the TEs of 55 genes were significantly increased, whereas those of 76 genes were significantly decreased under –P conditions ($|\log_2$ fold change $[FC]| \geq 1$, false discovery rate < 0.05) (Figure 4E; Supplemental Table 4). These findings are consistent with previous Ribo-seq results from *Arabidopsis thaliana* roots exposed to long-term (7-day) –P treatment (Bazin et al., 2017), in which fewer than 300 mRNAs displayed significant TE changes. We then evaluated the relationship between –P-mediated changes in RSS and TE. Fewer than 1% of –P-specific RSS-unfolding transcripts were associated with changes in TE under –P conditions (Figure 4F). Similar results were obtained for ReP relative to normal conditions (Figures 4E and 4F). Consistent with these observations, the global TEs of PSI RSS-unfolding transcripts did not differ between normal and –P conditions (Figure 4G). Overall, these results suggest that –P-specific RSS unfolding is not related to translational regulation.

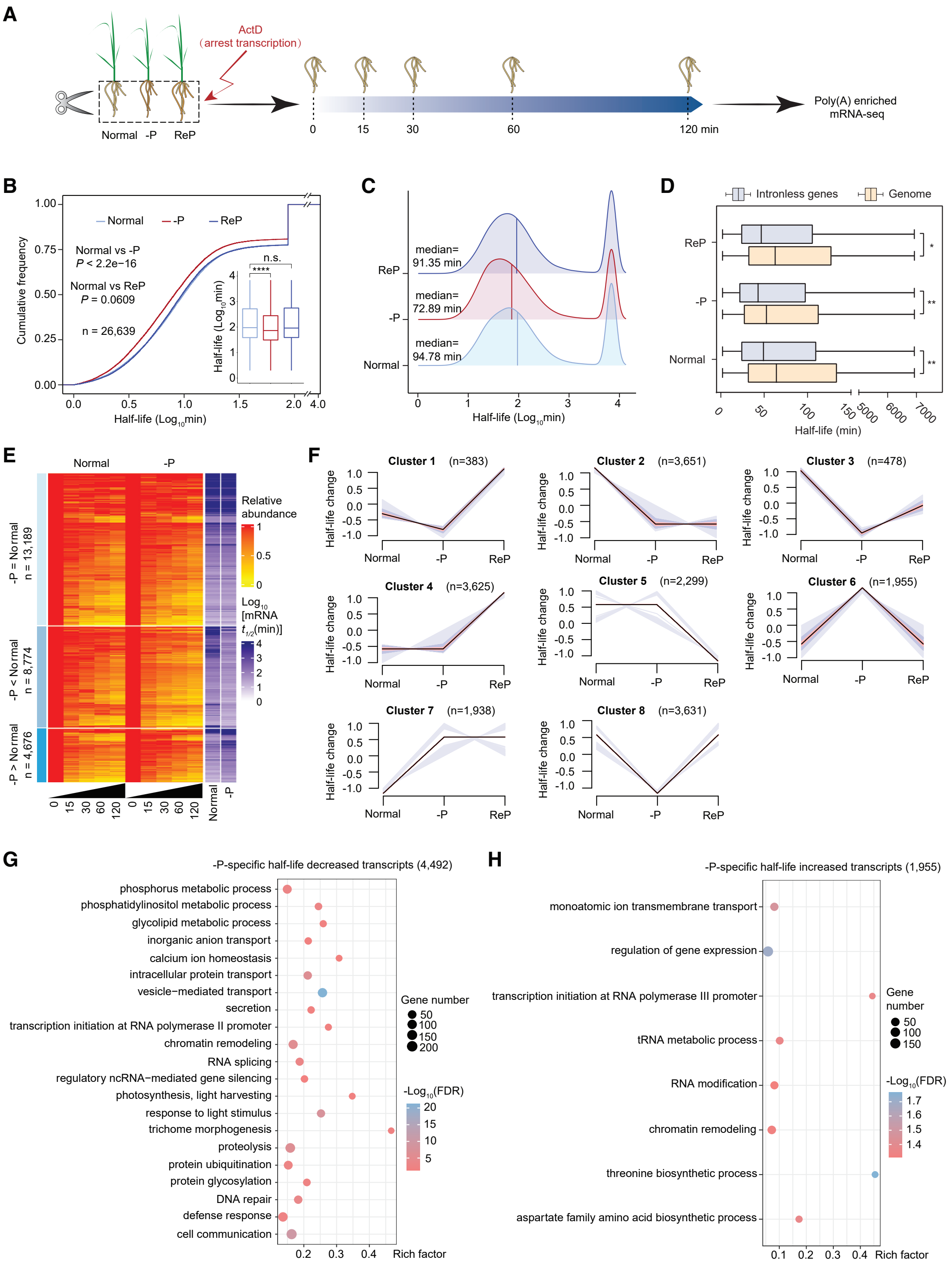

Figure 5. RNA stability–mediated PSR of rice roots

(A) Schematic overview of the workflow for RNA decay analysis.

(B) Cumulative curves and boxplots showing significantly reduced global RNA half-lives under –P compared with normal and ReP conditions. *p* values for cumulative curves were calculated using the Kolmogorov–Smirnov test; *p* values for boxplots were calculated using the Wilcoxon test.

(C) Ridgeline plots showing decreased global RNA half-lives under –P compared with normal and ReP conditions.

(D) Boxplots showing shorter half-lives of intronless genes ($t_{1/2}$ < 6931 min) compared with the transcriptome ($t_{1/2}$ < 6931 min) under normal, –P, and ReP conditions. *p* values for boxplots were calculated using the Wilcoxon test. *, $p < 0.05$; **, $p < 0.01$.

(legend continued on next page)

Regulation of global RNA stability in rice roots in response to –P

Several studies have revealed that RSS regulates RNA stability (Yang et al., 2022b; Wu et al., 2024), prompting us to investigate the relationship between –P-induced RSS unfolding and RNA stability. We performed a transcriptome-wide RNA decay assay in rice roots under normal, –P, and ReP conditions (Figure 5A). Roots were infiltrated with the transcriptional inhibitor actinomycin D (ActD), sampled at 0, 15, 30, 60, and 120 min, and subjected to poly(A)-enriched RNA-seq (Sorenson et al., 2018) (Figure 5A). We obtained three highly reproducible biological replicates for each condition, with an average of 38 million reads per replicate (Supplemental Figure 9A and 9B; Supplemental Table 1). After normalization of sequencing data and removal of low-abundance mRNAs, we used transcript abundances to model decay rates for each gene (see methods). Similar to previous genome-wide studies of RNA decay in plants (Sorenson et al., 2018; Wu et al., 2024), we observed a broad range of mRNA half-lives ($t_{1/2}$); the median mRNA half-life was 94.78 min under normal growth conditions (Figures 5B and 5C; Supplemental Table 5). Consistent with earlier findings that intronless mRNAs generally exhibit shorter half-lives than other mRNAs (Narsai et al., 2007; Wu et al., 2024), we observed the same pattern under all three growth conditions (Figure 5D). To further validate the fidelity of the RNA decay data, we randomly selected three transcripts with short, medium, and long half-lives under all three growth conditions; we confirmed their RNA stability via quantitative reverse-transcription PCR (RT-qPCR) (Supplemental Figure 9C and 9D). These results support the high fidelity and quality of the transcriptome-wide RNA decay dataset.

Global RNA stability was significantly reduced during –P treatment but was restored after ReP (Figures 5B and 5C). The median RNA half-life decreased to 72.89 min in response to –P treatment (Figure 5C), compared with 94.78 min under normal conditions, indicating that –P inhibits global RNA stability. The half-lives of 8, 774 transcripts were shortened, whereas those of 4,676 transcripts were prolonged, under –P compared with normal conditions (Figure 5E). As expected, transcripts with shortened or prolonged half-lives exhibited significant overlap between the –P vs. normal and –P vs. ReP comparisons (Supplemental Figure 10). We subsequently applied soft clustering—using the Mfuzz algorithm (fuzzy c-means) with Euclidean distance—to RNA half-life values across normal, –P, and ReP conditions to group transcripts whose half-lives were altered by –P and restored by Pi refeeding. These transcripts were classified as –P-specific half-life–decreased transcripts (4,492 genes in clusters 1, 3, and 8) or –P-specific half-life–increased transcripts (1,955 genes in cluster 6) (Figure 5F; Supplemental Table 5). GO analysis revealed that –P-specific RNA stability–decreased genes were enriched in pathways including phosphatidylinositol metabolism, glycolipid metabolism, anion transport, transcription by RNA polymerase (Pol) II, RNA splicing, RNA silencing, photosynthesis, proteolysis, protein transport, DNA repair, and cell communication (Figure 5G). In contrast, –P-specific RNA stability–increased genes were enriched in GO terms related to transcription by RNA Pol III, tRNA metabolism, RNA modification, and amino acid biosynthesis (Figure 5H). Taken together, these results indicate that RNA stability mediates the PSR in rice roots.

Downregulation of RNA half-life and RSS in roots under –P

We evaluated the relationship between –P-induced changes in RNA stability and –P-specific changes in RSS. Global RNA half-lives of PSI RSS-unfolding transcripts decreased under –P but were restored after ReP (Supplemental Figure 11A). Notably, the magnitude of the decrease in half-life was greater for these transcripts (median reduction of 26.33 min) than for the transcriptome overall (median reduction of 21.89 min), suggesting that RSS unfolding contributes to RNA decay (Figure 5C; Supplemental Figure 11A). Consistent with this interpretation, we observed substantial overlap between PSI RSS-unfolding transcripts and transcripts with decreased RNA half-lives under –P, but not with those exhibiting increased half-lives (Figure 6A). These 614 overlapping transcripts were designated –P-specific RSS-unfolding & $t_{1/2}$-down transcripts (Supplemental Table 5). Similar to the Gini index, the global half-lives of these transcripts were significantly reduced in response to –P and were fully restored after ReP (Figures 6B and 6C).

GO analysis showed that these –P-specific RSS-unfolding & $t_{1/2}$-down transcripts are involved in responses to abiotic stimuli, hormone responses, sphingolipid metabolism, mRNA metabolism, vesicle-mediated transport, and other processes (Figure 6D). Among these transcripts, we identified several known PSR genes that participate in Pi homeostasis and PSR signaling, such as *OsIPS2*, *OsSPX1*, *OsPAP9a*, and *OsPT15* (Figure 6E; Supplemental Figure 11B–11D; Supplemental Table 5). Several other genes, including the root hair formation- and tillering–related gene *OsRBOHE* (*Respiratory Burst Oxidase Homolog E*) (Zhao et al., 2025), the RNA decay–related gene *OsXRN3* (5′–3′ exoribonuclease) (Han et al., 2023), and the potassium transfer- and drought stress response–related gene *OsHAK1* (*High-affinity Potassium Transporter 1*) (Chen et al., 2015a, 2017), also exhibited concurrent reductions in RNA stability and Gini index under –P conditions (Supplemental Figure 11E−11G). These results indicate a broad role for –P-induced RSS unfolding in RNA stability regulation during Pi starvation.

PSI RSS unfolding represses RNA stability to fine-tune RNA expression and Pi homeostasis

Next, we examined the levels of –P-specific RSS-unfolding & $t_{1/2}$-down transcripts. Compared with normal conditions, global steady-state levels of these transcripts significantly increased

(E) Heatmap showing RNA decay over 120 min in Nip under normal and –P conditions. RNA decay dynamics were quantified by tracking changes in mean relative RNA abundance over time, with decay rates expressed as RNA half-life ($t_{1/2}$ in minutes).
(F) Identification of gene clusters with –P-induced changes in half-life using the R package Mfuzz.
(G and H) GO analysis of transcripts with –P-specific decreases (G) and increases (H) in half-life. In (B) and (D), midlines and box edges indicate medians and quartiles, respectively. Whiskers extend to the farthest data point within 1.5 times the IQR from the box edges. –P, Pi deficiency; ReP, Pi-refeeding following Pi deficiency.

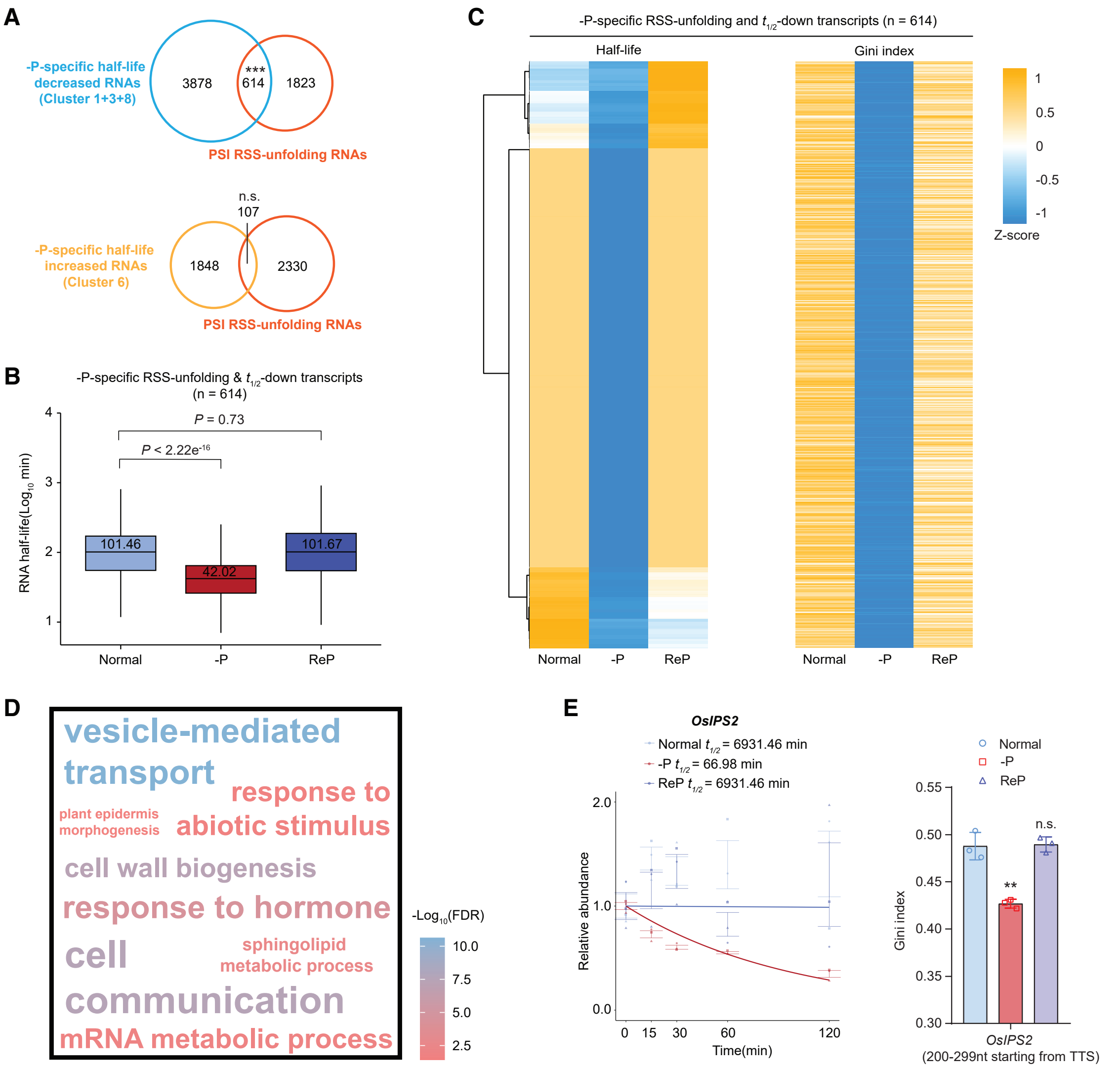

Figure 6. Synergistic downregulation of RNA stability and RSS under –P conditions

(A) Venn diagrams showing significant overlap of PSI RSS-unfolding RNAs with –P-specific RNAs exhibiting reduced half-lives, but not with –P-specific RNAs displaying increased half-lives. p values were calculated using a hypergeometric test.

(B) Boxplots showing significant global decreases in half-lives of –P-specific RSS-unfolding & $t_{1/2}$-down transcripts under –P, but not ReP, compared with normal conditions. p values were calculated using the Wilcoxon test. Midlines and box edges indicate medians and quartiles, respectively. Whiskers extend to the farthest data point within 1.5 times the IQR from the box edges.

(C) Heatmap showing coordinated downregulation of RNA stability and RSS for –P-specific RSS-unfolding & $t_{1/2}$-down transcripts under –P conditions.

(D) GO analysis of –P-specific RSS-unfolding & $t_{1/2}$-down transcripts. Font size indicates the number of associated transcripts.

(E) Both RNA half-life profiles and Gini indices of *OsIPS2* decreased in response to –P and were restored after ReP. In half-life profiles, relative RNA abundances following transcriptional inhibition are shown, with bars indicating means ± standard error of the mean (n = 3); thick lines indicate modeled values. Half-lives ($t_{1/2}$) are indicated for each treatment. In Gini index bar plots: TTS, transcription termination site; p values were calculated using an unpaired two-tailed Student's t test. **, $p < 0.01$; n.s., not significant. –P, Pi deficiency; ReP, Pi-refeeding following Pi deficiency.

under –P conditions and returned to basal levels after ReP (Figure 7A). This apparent discrepancy between decreased RNA stability and increased steady-state abundance indicates that feedback regulation mediated by RSS unfolding affects gene expression. Although expression changes for approximately 92% of these transcripts were less than 1.5-fold (–P vs. normal, $\log_2$[FC] < 0.585, $p < 0.05$) (Figure 7B), RNA levels of the majority of –P-specific RSS-unfolding & $t_{1/2}$-down transcripts (~68%) tended to increase under –P compared with normal conditions (–P vs. normal, $\log_2$[FC] > 0) (Figure 7B). This pattern was not evident at the transcriptome-wide level (Figure 7C). Instead, a slight overall decrease in expression was detected; 1,831 and 2,547 transcripts were significantly upregulated and downregulated, respectively, by more than 1.5 fold (–P vs. normal, $\log_2|[FC]| \geq 0.585$) under –P conditions (Figure 7C). The inverse relationship between expression level and RNA stability of –P-specific RSS-unfolding transcripts suggests that PSI RSS unfolding represses RNA stability, providing feedback to fine-tune steady-state transcript levels.

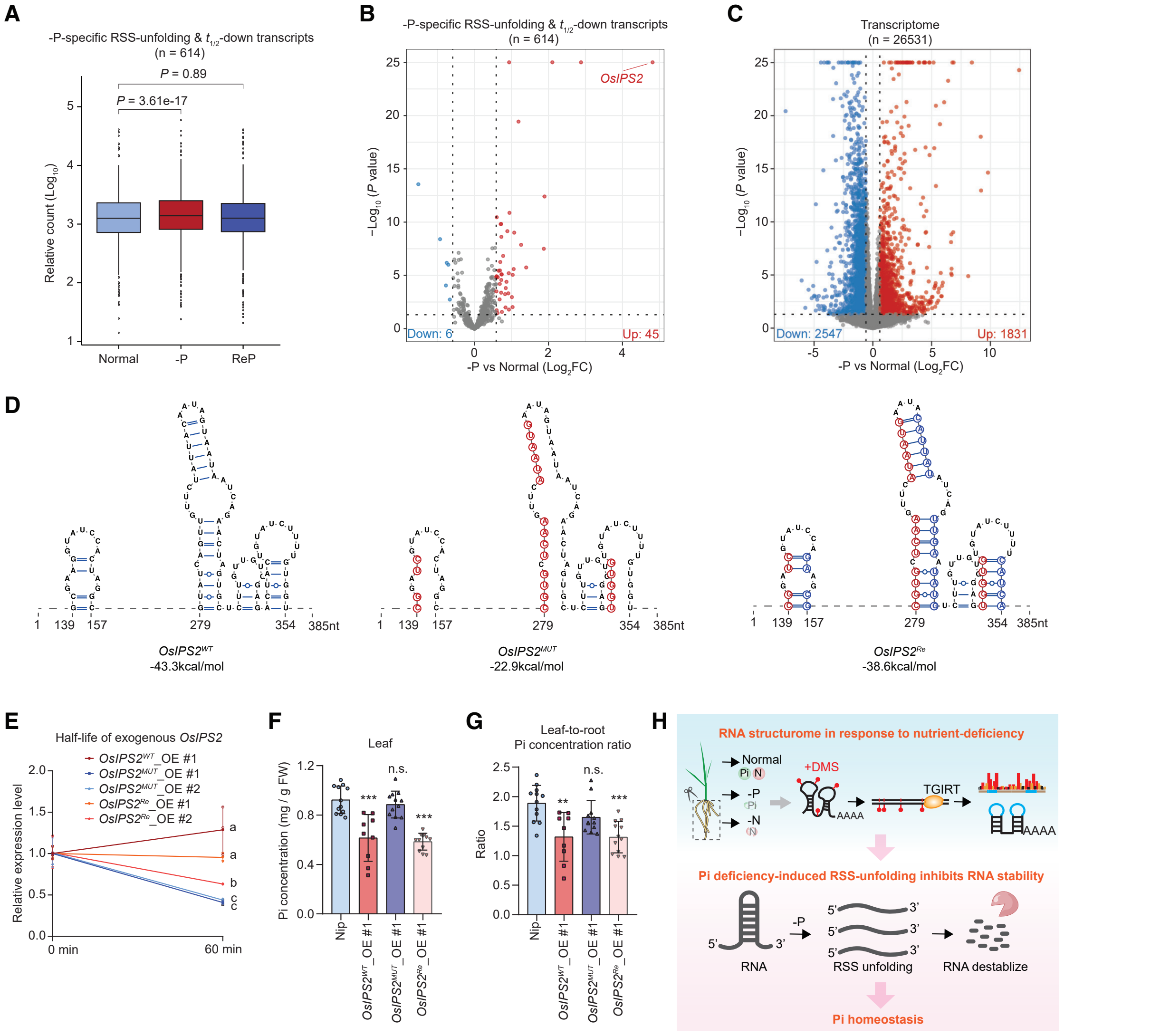

Figure 7. –P-specific RSS unfolding represses RNA stability to fine-tune steady-state PSR transcript levels and Pi homeostasis

(A) Boxplots showing a moderate global increase in steady-state levels of –P-specific RSS-unfolding & $t_{1/2}$-down transcripts under –P, but not ReP, compared with normal conditions. Midlines and box edges indicate medians and quartiles, respectively. Whiskers extend to the farthest data point within 1.5 times the IQR from box edges.

(B and C) Volcano plots showing expression changes in –P-specific RSS-unfolding & $t_{1/2}$-down transcripts (B) and across the transcriptome (C). Up, upregulated transcripts (–P vs. normal, $\log_2FC > 0.585$, $p < 0.05$); down, downregulated transcripts (–P vs. normal, $-\log_2FC > 0.585$, $p < 0.05$). Differential expression analysis was performed using DESeq2, with p values calculated via the Wald test.

(D) Modeled RSS of PSI RSS-unfolding regions (139–157 and 279–354 nt from the TSS) of *OsIPS2* (*OsIPS2*^{WT}) and corresponding RSS-mutated (*OsIPS2*^{MUT}) and RSS compensatory-mutated (*OsIPS2*^{Re}) variants. Predicted ΔG values are shown. RSS-mutated nucleotides are highlighted by red circles, and compensatory-mutated nucleotides are highlighted by blue circles.

(E) Target-specific RNA decay showing half-lives of exogenous *OsIPS2* in *OsIPS2*^{WT}_OE, *OsIPS2*^{MUT}_OE, and *OsIPS2*^{Re}_OE transgenic lines. Relative expression levels of transcripts were normalized to the 0-min time point (ratio arbitrarily set to 1), with standard deviations calculated from three biological replicates. Different lowercase letters indicate significant differences based on an unpaired two-tailed Student's t test ($p < 0.05$).

(F and G) Leaf Pi concentration (F) and leaf-to-root Pi concentration ratio (G) in Nip, *OsIPS2*^{WT}_OE #1, *OsIPS2*^{MUT}_OE #1, and *OsIPS2*^{Re}_OE #1 plants. **, $p < 0.01$; ***, $p < 0.001$; unpaired two-tailed Student's t test. n.s., not significant.

(H) Schematic summarizing the high-quality RNA structurome under nutrient-deficient conditions, indicating that Pi-starvation–induced RSS-unfolding reduces RNA stability and contributes to fine-tuning Pi homeostasis in rice roots. –P, Pi deficiency; ReP, Pi-refeeding following Pi deficiency; –N, nitrogen deficiency; DMS, dimethyl sulfate.

To validate the role of PSI RSS unfolding in RNA stability and Pi homeostasis, we performed RSS mutagenesis and generated transgenic plants to examine PSI RSS-unfolding–mediated changes in RNA stability *in vivo*. Considering potential side effects of RSS mutations on amino acid sequences and the feasibility of functional assessment, we selected the well-characterized –P-induced lncRNA *OsIPS2*. This transcript represents a –P-specific RSS-unfolding & $t_{1/2}$-down RNA (Figures 3H and 6E) whose expression is induced by OsPHR2 under –P conditions (Figure 7B) (Zhou et al., 2008). The –P-induced RSS-unfolding

regions of *OsIPS2*, located at nucleotides 139–157 and 279–354 from the transcription start site (TSS) and excluding the miR399-binding region (Franco-Zorrilla et al., 2007), form four stem-loop structures under normal conditions but adopt a single-stranded conformation under –P stress (Figure 3H). We introduced point mutations into this region to mimic the –P-induced single-stranded conformation ($IPS2^{MUT}$), along with compensatory mutations to restore the RSS of this region to the stem-loop structure ($IPS2^{Re}$) (Figure 7D). The minimum thermodynamic free energy (ΔG) of these PSI RSS-unfolding regions increased from −43.3 kcal/mol ($OsIPS2^{WT}$) to −22.9 kcal/mol ($OsIPS2^{MUT}$) and was restored to −38.6 kcal/mol ($OsIPS2^{Re}$) by compensatory mutation (Figure 7D), indicating successful modulation of RSS.

We overexpressed full-length WT, mutated (MUT), and compensatory mutated (Re) *OsIPS2* transcripts driven by the 35S promoter to generate stable transgenic plants. We randomly selected one $OsIPS2^{WT}$_OE line and two individual $OsIPS2^{MUT}$_OE and $OsIPS2^{Re}$_OE lines to measure RNA half-lives of exogenously expressed *OsIPS2* under normal conditions. Compared with the stable RNA half-life of $OsIPS2^{WT}$, the half-life of $OsIPS2^{MUT}$ (harboring an unfolded RSS) was significantly reduced (Figure 7E), whereas the half-life of $OsIPS2^{Re}$ (harboring a restored stem-loop RSS) was comparable to that of $OsIPS2^{WT}$ (Figure 7E). Half-lives of the control unstable transcript (Os01g0389200) were similar across all examined transgenic lines, indicating effective transcriptional arrest via chemical treatment (Supplemental Figure 12A). In parallel, compared with $OsIPS2^{WT}$, the steady-state expression of $OsIPS2^{MUT}$ was significantly reduced in $OsIPS2^{MUT}$_OE plants, whereas expression of the control gene *HPT* was significantly elevated (Supplemental Figure 12B and 12C). The opposite trend was observed in $OsIPS2^{Re}$_OE transgenic plants (Supplemental Figure 12B and 12C). Collectively, these results confirm that –P-induced RSS unfolding represses RNA stability.

OsIPS1/2 serve as key repressors of Pi translocation from roots to shoots (Franco-Zorrilla et al., 2007). Consistent with earlier findings, we observed significant decreases in leaf Pi concentration and the leaf-to-root Pi concentration ratio in both $OsIPS2^{WT}$_OE and $OsIPS2^{Re}$_OE plants relative to Nip (Figures 7F and 7G). In contrast, $OsIPS2^{MUT}$_OE plants exhibited leaf Pi concentrations and leaf-to-root Pi ratios comparable to those of Nip (Figures 7F and 7G). These results indicate that –P-induced RSS unfolding regulates Pi homeostasis (Figure 7H).

DISCUSSION

Rice is a staple cereal crop that sustains more than half of the world's population (FAO, 2018; Sen et al., 2020). Nutrient deficiency remains a major constraint on global rice production and quality (Fahad et al., 2019). Elucidation of the mechanisms underlying crop responses to fluctuations in nutrient availability is essential for developing varieties with high nutrient-use efficiency. In this study, we obtained high-quality, deep-coverage *in vivo* RNA structuromes from rice roots under normal and nutrient-deficient conditions (–P and –N) using optimized DMS-MaPseq methods (Figure 1). High-conf. RSS information covered more than 60% of expressed transcripts under each growth condition. –P induced global increases in RSS diversity and unfolding, predominantly within CDS regions of mRNAs (Figures 2 and 3). To investigate the regulatory functions of PSI RSS unfolding, we performed translatome profiling and transcriptome-wide RNA decay analyses under –P stress (Figures 4 and 5). Integrated multi-omics analyses and studies of RSS-mutated transgenic plants revealed that repression of PSI RSS-unfolding–mediated RNA stability fine-tunes the expression of PSR-related transcripts and Pi homeostasis under –P conditions (Figures 6 and 7). Collectively, these findings highlight a regulatory role for RSS-unfolding–mediated RNA decay in the PSR of rice roots.

In vivo RNA structure is dynamic and influenced by the cellular physicochemical environment (Bevilacqua et al., 2016; Vandivier et al., 2016; Wang et al., 2021). Using *in vivo* RSS probing technologies, several recent studies have investigated RNA structure reprogramming in response to abiotic and biotic stresses in plants (Zhu et al., 2021; Zhang and Ding, 2025). Our high-quality, in-depth RNA structurome data revealed dynamic alterations of *in vivo* RSS in rice roots under –P and –N conditions. In particular, –P triggered widespread RSS unfolding (Figure 2). Transcripts harboring PSI RNA-unfolding regions participate in multiple PSR processes (Figure 3G), as well as RNA metabolism, translation, and vesicle-mediated transport (Supplemental Figure 6D), indicating broad functions of RSS in plant adaptation to –P stress. Feature analysis showed that PSI RNA-unfolded regions exhibit low GC content and short stem length, consistent with flexible RSS (Figure 3). Similarly, –N-induced RSS-unfolding regions also displayed low GC content and flexible RSS features (Supplemental Figures 5A–5C). These observations are consistent with previous genome-wide RSS profiling studies using Structure-seq in *Arabidopsis* (Ding et al., 2014; Tack et al., 2020) and rice (Deng et al., 2018), which detected that mRNAs with highly flexible RSS tend to be associated with plant responses to environmental stimuli. Although RSS features share common characteristics in –P- and –N-induced RSS remodeling, PSI- and –N-unique RSS-unfolding transcripts were enriched in distinct stress-specific biological processes (Supplemental Figures 5E and 6D), suggesting divergent regulatory roles of RSS unfolding under different stress conditions.

Notably, the –P-induced transcriptome-wide increase in RSS diversity suggests that PSI RNA unfolding is an active, rather than passive, process (Figure 2). Ribo-seq further indicated that PSI RNA unfolding is not a secondary consequence of enhanced translation (Figure 4). It is possible that currently uncharacterized PSR-related RNA-binding proteins (RBPs) and/or RNA helicases contribute to this process. Indeed, we found that several RNA helicase- and RBP-encoding genes were significantly upregulated under –P conditions (Supplemental Figure 13), making them promising candidates for future functional studies. Additionally, RNA conformation is influenced by cations, heavy metals, and diverse metabolites (Bevilacqua et al., 2016). Given that P is a key element for the biosynthesis of essential metabolites, –P causes substantial changes in primary and secondary metabolism in plants (Pant et al., 2015; Dissanayaka et al., 2021). These metabolic alterations might also contribute to global changes in RSS. Taken together, these mechanisms may underlie the specific RSS responses observed under –P and –N nutritional stresses.

RNA structure is crucial for governing nearly all aspects of RNA metabolism (Xu et al., 2022). Genome-wide RNA decay assays and analyses of transgenic plants exhibiting RSS mutagenesis revealed RNA structure–mediated regulation of PSR in rice roots (Figures 5, 6, and 7). Accelerated Pi recycling from organic forms is an important PSR in plants (Paz-Ares et al., 2022), but the underlying mechanisms remain underexplored. RNA constitutes a major intracellular P pool. Endonucleases, such as S-like RNases (RNSs), are upregulated upon Pi starvation to degrade cytosolic or extracellular RNAs (Supplemental Figure 1D) (Bariola et al., 1994; Gho et al., 2020). Indeed, our transcriptome-wide RNA decay assay revealed a decrease in global RNA half-life under –P conditions (Figure 5). Notably, PSI RSS-unfolding predominantly occurred within CDS regions, rather than UTRs. This pattern differs from the RNA decay driven by temperature-induced RSS unfolding at UTR regions in *Arabidopsis* (Yang et al., 2022b) and rice (Su et al., 2018), suggesting that CDS regions exert regulatory functions through RSS beyond their canonical role in protein encoding. Furthermore, –P-induced RNSs belong to the RNase T2 family, which comprises nonspecific single-stranded RNA endonucleases (Megel et al., 2019). PSI RSS-unfolding within CDS regions could enhance transcript accessibility to RNSs, thereby facilitating RNA degradation. In addition to endonuclease induction, RSS unfolding within CDS regions might represent an additional regulatory mechanism that accelerates RNA decay and Pi recycling under –P conditions.

Furthermore, global steady-state levels of transcripts with reduced RNA stability due to PSI RSS-unfolding increased under –P conditions (Figure 7A). These transcripts included many important PSR- and stress response–related genes (Figure 6; Supplemental Figure 11). Our analysis of transgenic plants carrying *OsIPS2* RSS mutations confirmed that PSI RSS-unfolding decreases RNA stability and attenuates its function in Pi translocation from roots to shoots (Figure 7D–7G). These findings suggest that feedback mediated by PSI RSS-unfolding fine-tunes the expression of key PSR transcripts, thereby maintaining appropriate root responses to Pi deficiency. Such RSS-mediated feedback regulation may also enable rapid downregulation of pivotal PSI transcripts after Pi refeeding. Future validation of additional PSI RSS-mediated functions in other PSR-related transcripts, including noncoding RNAs, could help identify candidate RSS elements for enhancing low-Pi tolerance. Researchers could use CRISPR-Cas9–based precise mutagenesis to modify PSR-related RSS regions, modulating transcript stability and thereby enhancing nutrient uptake in rice.

In conclusion, we generated a high-quality, high-coverage RNA structurome of rice roots under normal and nutrient-deficient conditions. Integrated analyses of RSS features, the translatome, and transcriptome-wide RNA decay assays, along with analyses of transgenic plants exhibiting RSS mutagenesis, uncovered repressed RSS-unfolding–mediated RNA stability as an adaptive response of rice to Pi deficiency stress. These findings provide novel insights into the regulatory roles of *in vivo* RSS in mediating plant responses to nutrient deficiency. Additionally, the transcriptome-wide *in vivo* RSS landscape under nutrient-deficient conditions represents a valuable resource for related crop studies and offers potential targets for crop improvement.

METHODS

Plant materials and growth conditions

The *japonica* rice (*Oryza sativa* subsp. *japonica*) cultivar Nip was used in this study. Seeds were soaked in 1% (v/v) HNO_3 solution for 16 h to break dormancy, germinated in pure water at 37°C for 3 days, and then cultured hydroponically. Germinated seedlings were grown in a hydroponic nutrient solution (0.8225 mM NH_4NO_3, 0.2 mM $NaH_2PO_4{\cdot}2H_2O$, 0.1915 mM K_2SO_4, 0.547 mM $MgSO_4{\cdot}7H_2O$, 0.366 mM $CaCl_2{\cdot}2H_2O$, 0.0005 mM $MnCl_2{\cdot}4H_2O$, 0.003 mM H_3BO_3, 0.0001 mM $(NH_4)_6Mo_7O_{24}{\cdot}4H_2O$, 0.0004 mM $ZnSO_4{\cdot}7H_2O$, 0.0002 mM $CuSO_4{\cdot}5H_2O$, 0.04 mM EDTA-Fe, and 0.2 mM MES; pH adjusted to 5.5 with 5 M NaOH) (Yoshida et al., 1976).

For the RSS mutagenesis assay, Nip plants were transformed with the binary vectors P_{35S}-OsIPS2^{WT}, P_{35S}-OsIPS2^{MUT}, and P_{35S}-OsIPS2^{Re} by *Agrobacterium tumefaciens* (strain EHA105)–mediated transformation (Toki et al., 2006) to generate the corresponding *OsIPS2^{WT}*_OE, *OsIPS2^{MUT}*_OE, and *OsIPS2^{Re}*_OE transgenic lines. Positive transgenic plants were confirmed by genomic PCR in the T0 generation, followed by RT-qPCR in the T1 generation to verify exogenous expression of *OsIPS2*. The transgenic line *P_{35S}-3×FLAG-OsRPL18* in the Nip background was generated in a similar manner for TRAP-based Ribo-seq (described in the following subsection: Construction of P_{35S}-3×FLAG-OsRPL18). Transgenic lines in the T1 generation were identified by immunoblotting using an anti-FLAG antibody (ABclonal, cat# AE005).

For hydroponic culture, rice seedlings were grown in a greenhouse under a 14-h light (30°C)/10-h dark (24°C) photoperiod, with approximately 300 μmol m^{-2} s^{-1}photon flux density and ~60% relative humidity. Ten-day-old seedlings were subjected to normal (200 μM Pi, 822.5 μM N), Pi-deficient (–P, 0 μM Pi, 822.5 μM N), or N-deficient (–N, 200 μM Pi, 41.125 μM N) conditions for 5 days. For Pi refeeding (ReP), –P-treated seedlings were transferred to normal conditions and cultured for an additional 2 days.

Vector construction

Construction of P_{35S}-OsIPS2^{WT}, P_{35S}-OsIPS2^{MUT}, and P_{35S}-OsIPS2^{Re}

Full-length *OsIPS2* carrying an RSS mutation (*OsIPS2^{MUT}*) or a compensatory RSS mutation (*OsIPS2^{Re}*) was synthesized (GeneScript); the corresponding sequences are provided in Supplemental Table 6. Full-length *OsIPS2^{MUT}* and *OsIPS2^{Re}* fragments were amplified from the synthesized plasmids. The full-length *OsIPS2^{WT}* sequence was amplified from Nip cDNA using KOD-Plus-Neo Hot Start polymerase (TOYOBO). PCR products of *OsIPS2^{WT}*, *OsIPS2^{MUT}*, and *OsIPS2^{Re}* were inserted into the BamHI/SalI-digested pCAMBIA1300-P_{35S} vector to generate the P_{35S}-OsIPS2^{WT}, P_{35S}-OsIPS2^{MUT}, and P_{35S}-OsIPS2^{Re} constructs, respectively.

Construction of P_{35S}-3×FLAG-OsRPL18

The CDS of *OsRPL18* (Os05g0155100) was amplified from Nip cDNA using KOD-Plus-Neo Hot Start polymerase (TOYOBO) and ligated into pCAMBIA-P_{35S}-3×FLAG to obtain P_{35S}-3×FLAG-OsRPL18.

All constructs were confirmed by sequencing. Primers used to generate all constructs are listed in Supplemental Table 6.

Measurement of Pi and total P contents

Cellular Pi and total P concentrations in rice roots and leaves were measured as previously described (He et al., 2021). Briefly, cellular Pi concentrations were determined using a continuous flow analyzer (SAN++, SKALAR, Breda, the Netherlands). Total P concentrations were measured by Inductively Coupled Plasma–Optical Emission Spectrometry (Optima 7300DV; PerkinElmer, Waltham, MA, USA).

Measurement of nitrogen content

Root samples were harvested and dried at 65°C for 1 week. Dried samples were ground to a fine powder using a tissue grinder (JINXIN). Total N

content was determined by Elemental Analysis–Isotope Ratio Mass Spectrometry (EA-IRMS; Thermo Scientific Flash 2000/Delta V Advantage).

In vivo DMS modification

In vivo DMS modification was performed as previously described (Wang et al., 2019; Jin et al., 2022). Briefly, after nutrient-deficient treatments, the roots of rice seedlings were collected and cut into ~1.5-cm segments. Samples were immersed in 20 mL of 1× DMS reaction solution (40 mM HEPES pH 7.5; 100 mM KCl; and 0.5 mM $MgCl_2$ in DEPC-treated H_2O) in a 50-mL Corning tube. After 200 μL DMS (MACKIN) had been added to a final concentration of 1%, the reaction solution was vigorously vortexed until the DMS was fully dissolved. Mock treatments were performed by adding an equal volume of deionized water instead of DMS. Samples were treated in DMS reaction buffer or mock solution at room temperature (RT) for 5 min under vacuum (~12 psi), then incubated at 30°C with shaking at 250 rpm for 10 min. After addition of 5 mL β-mercaptoethanol (Sigma) to quench the DMS reaction, samples were incubated for 5 min under vacuum at RT. Samples were washed three times with 50 mL DEPC-treated H_2O, blotted dry with paper towels, and immediately frozen in liquid nitrogen. Three biological replicates were prepared for each DMS-treated sample, and one biological replicate was prepared for each mock-treated sample.

All manipulations involving DMS were conducted using appropriate safety equipment, including lab coats and double gloves. All disposable materials were discarded as hazardous waste. DMS treatments were performed in a chemical fume hood with strong airflow (>200 feet per minute).

Genome-wide DMS-MaPseq library generation and sequencing

Genome-wide DMS-MaPseq library generation was performed as previously described, with minor modifications (Wang et al., 2018, 2019; Jin et al., 2022). DMS-treated and untreated root samples were ground into powder; total RNA was isolated using TRIzol reagent (Thermo Fisher) in accordance with the manufacturer's instructions. Total RNA was treated with TURBO DNase (Thermo Fisher) and purified using an RNeasy Mini kit (Qiagen). Each 1 μg DNase-treated RNA sample was processed using the TruSeq Stranded Total RNA Sample Prep Kit (Illumina). rRNA was removed using the Ribo-Zero Plant Kit (Illumina). For reverse transcription, TGIRT-III enzyme (InGex) was used instead of SuperScript II. Illumina sequencing adaptors and indices were introduced by performing no more than 15 cycles of PCR amplification. Genome-wide DMS-MaPseq libraries were sequenced in 2 × 150-nt paired-end mode on the NovaSeq platform at Novogene.

DMS-MaPseq data analysis

An average of 600 million paired-end 150-nt raw reads were obtained for each DMS-MaPseq library. TrimGalore (v0.6.6) (Martin, 2011) was used to remove the Illumina universal adaptor sequence (AGATCGGAAGAGC ACACGTCTGAACTCCAGTCA) and to filter low-quality reads (quality score <25). For TGIRT-generated libraries, 2-nt sequences were trimmed from the 5′ end (Zubradt et al., 2017). The "Quality Filter" function of the FASTX-Toolkit (v0.0.14) (Gordon and Hannon, 2010) was used for additional filtering; at least 80% of bases were required to display a quality score >25.

TopHat2 (v2.1.1) (Kim et al., 2013) was used to align reads to the IRGSP-1.0 reference genome (Kawahara et al., 2013). The following parameters were applied: "tophat2 -p 20 –library-type fr-firststrand –no-novel-juncs -N 15 –read-gap-length 10 –read-edit-dist 15 –max-insertion-length 5 –max-deletion-length 5 -g 3," allowing up to 10% mismatches. Uniquely mapped reads were selected using "grep -E " ˆ@|NH:i:1."

To prevent overlap of mismatch signals derived from forward- and reverse-strand transcripts at the same genome positions, the "view" function of SAMtools (v1.9) (Li et al., 2009) was used to separate uniquely mapped BAM files into forward- and reverse-strand files according to SAM flags.

Mismatch and coverage counts for each nucleotide were calculated using the in-house Python script "CountMismatch2Bed.py" (https://github.com/changhaoli/TAMU_02RSS) and the BEDTools (v2.29.2) (Quinlan and Hall, 2010) "genomecov" function with the parameters "-d -split." Raw DMS reactivity was calculated by dividing mismatch counts by coverage at each nucleotide position. Genome-wide DMS reactivity was assumed to be comparable across nutrient conditions. Therefore, DMS reactivity values for each sample were normalized by the ratio of the average DMS reactivity in that sample to the average DMS reactivity in samples under normal conditions.

Analysis of changes in RSS

To identify transcript regions with significant RNA structural differences across conditions, a sliding window approach was used to calculate the Gini index across the transcriptome, as previously described (Zubradt et al., 2017) with minor modifications. Specifically, each gene was divided into non-overlapping 100-nt windows according to genomic coordinates (from smallest to largest positions) based on the IRGSP-1.0 reference genome. For genes located on the positive strand, window segmentation began at the annotated TSS and proceeded sequentially in the 5′ to 3′ direction of the transcript. For genes on the negative strand, window partitioning began at the annotated transcription termination site (TTS) and extended along increasing genomic coordinates, corresponding to the 3′ to 5' orientation of the transcript. Only windows with an average minimum of 20 mismatch counts per nucleotide, restricted to A and C mismatches, were used to calculate the Gini index.

For each window, two metrics were calculated to identify RSS alterations across conditions. First, the average Gini index for each window was calculated across three biological replicates under each condition. The change in Gini index (ΔGini) was then computed as the Gini index of the nutrient-deficient sample minus the Gini index of the normal sample. Second, the statistical significance of Gini index differences between conditions was determined by Student's *t* test. Thresholds used to define significant differential windows were a p value < 0.05 and $|\Delta Gini| \geq 0.05$. The 100-nt windows in regions with structural changes spanning both CDS and UTRs were classified as UTR regions.

Secondary structure modeling

Raw DMS-induced mismatch ratios were normalized prior to RNA structure modeling (Lan et al., 2022). All raw DMS reactivities were divided by the median of the highest 5% of mutation rates to obtain normalized DMS reactivities. Normalized reactivities exceeding 1.0 were winsorized by setting values to 1.0.

RSS was predicted from normalized DMS reactivity values using the fold function of RNAstructure (v6.1) (Reuter and Mathews, 2010). To minimize noise signals not derived from A and C, DMS reactivities for guanine and thymine bases were set to −999 (unavailable constraints).

RNA structures were visualized using VARNA (v3-93) (Darty et al., 2009). Bases were color-coded according to normalized DMS reactivities.

Analysis of *in vivo* RSS diversity

Alternative RSS conformations of transcripts were detected across the transcriptome using DREEM (Tomezsko et al., 2020). As in the Gini index analysis, the transcriptome (TIGR reference) was divided into 100-nt windows. Only windows covered by more than 50,000 or 25,000 reads were analyzed by DREEM with default parameters. For each window, iteration results yielding the highest number of clusters were used for subsequent analyses.

To evaluate RSS diversity, the Shannon diversity index was calculated as follows:

$$\text{Shannon index} = -\sum_{i=1}^{c} p_i \ln p_i$$

Here, c is the cluster number (1, 2, 3, or 4), and $p_i$is the percentage of i clusters.

RNA secondary structure feature analysis

To examine RNA secondary structure features of PSI RSS-unfolding windows, structures for each 100-nt window were modeled using RNAstructure (v6.1) (Reuter and Mathews, 2010), with corresponding FASTA sequences and normalized DMS reactivity data as inputs. Predicted structures were output in dot-bracket notation, then analyzed using ViennaRNA/forgi (v2.1.1) (Thiel et al., 2019) to quantify RSS features.

Ribo-seq library construction and sequencing

TRAP-based Ribo-seq was performed as previously described (Juntawong et al., 2015). Library construction comprised three major steps: ribosome-associated RNA isolation, small-RNA library preparation, and PCR amplification.

Normal, –P-, and ReP-treated P_{35S}-3×*FLAG-OsRPL18* rice roots were harvested and ground to powder in liquid nitrogen. To isolate ribosome-associated RNA, 0.4 g of powdered sample was lysed in 2 mL polysome extraction buffer (20 mM Tris-HCl pH 7.4, 150 mM NaCl, 5 mM $MgCl_2$, 1% Triton X-100, 1 mM DTT, 100 μg/mL cycloheximide [Sigma], and 1 pellet/50 mL complete EDTA-free protease inhibitor). After incubation on ice for 10 min and centrifugation at 16,000 × g for 15 min at 4°C, the supernatant was treated with RNase I (Epicentre) to digest exposed RNA regions and to dissociate polysomes into monosomes. After addition of 18 μL SUPERase-In RNase inhibitor (Thermo Fisher) to terminate the reaction, anti-FLAG antibody–conjugated magnetic beads were added to the supernatant; the mixture was then incubated for 2 h with gentle rocking at 4°C to enrich monosomes. Enriched beads were washed three times with wash buffer (20 mM Tris-HCl pH 8.0, 140 mM KCl, 35 mM $MgCl_2$, 50 μg/mL cycloheximide, 50 μg/mL chloramphenicol, and 1 pellet/50 mL complete EDTA-free protease inhibitor). Subsequently, TRIzol (Thermo Fisher) was used for RPF extraction. RPFs were resolved by electrophoresis on a 10% urea-PAGE gel. Cy3-labeled 25- and 35-nt RNA markers were used to guide size selection, and RPFs were recovered via gel extraction.

The RPFs were treated with T4 polynucleotide kinase (New England Biolabs) to remove 3′ phosphoryl groups. Following dephosphorylation, RNA fragments were ligated to 3′ DNA adaptors using T4 RNA ligase 2 (truncated KQ). After gel excision to remove free 3′ DNA adaptors, cDNA was synthesized using SuperScript II reverse transcriptase with a Ribo-seq RT primer. rDNA was depleted from cDNA using a streptavidin-biotin approach with biotinylated in-house anti-rRNA probes and Dynabeads MyONE magnetic beads C1 (Thermo Fisher). rDNA-depleted cDNA was circularized using CircLigase (Lucigen) and amplified with KOD-Plus-Neo Hot Start polymerase (TOYOBO). PCR products were digested with PmeI (New England Biolabs) to remove 25- and 35-nt RNA markers derived from the original library.

Finally, PCR products were purified by 3% agarose gel purification and sequenced in 2 × 150-nt paired-end mode on the Illumina NovaSeq platform at Annoroad. Sequences of the 3′ adaptor, Ribo-seq RT primer, 5′ PCR primer, and 3′ PCR primer used in this study are listed in Supplemental Table 6.

Ribo-seq data analysis

Adaptor trimming and removal of low-quality reads (quality score <20) from raw Ribo-seq and input mRNA-seq reads were performed using TrimGalore (v0.6.6) (Zhang et al., 2021). For paired-end Ribo-seq libraries, only read 1 was used in downstream analysis.

PCR duplicates were removed based on unique molecular identifiers (UMIs) using the "clumpify.sh" script from the BBMap (v38.18) (Bushnell, 2014) package. The Fastx_trimmer command from FASTX-Toolkit (v0.0.14) (Gordon and Hannon, 2010) was used to trim UMIs at the beginning and end of each read. Deduplicated reads were aligned to the IRGSP-1.0 reference genome (Kawahara et al., 2013) via STAR (v2.7.10b) (Dobin et al., 2013). Multi-mapped reads were excluded, and only uniquely mapped reads were retained for subsequent analyses. The R package DESeq2 (v1.46.0) (Love et al., 2014) was employed for sample clustering and PCA to examine reproducibility among biological replicates. The R package riboWaltz (v2.0) (Lauria et al., 2018) was used to evaluate Ribo-seq data quality, including assessment of RPF length distribution and the percentage of P-sites located within annotated transcript regions (5′ UTR, CDS, and 3′ UTR). Only reads mapping to CDSs were used to calculate TE, defined as the ratio of normalized Ribo-seq read counts to normalized RNA-seq read counts (Ingolia et al., 2009).

Ribo-seq–associated mRNA-seq library construction and analysis

To construct Ribo-seq–associated mRNA-seq libraries, total RNA was extracted from samples using TRIzol (Thermo Fisher). Genomic DNA was removed by treatment with TURBO DNase (Thermo Fisher). DNase-treated RNA was processed via library construction steps, including mRNA isolation and fragmentation, first- and second-strand cDNA synthesis, end repair, adaptor ligation, and PCR amplification. Libraries were prepared using a Stranded mRNA-seq Lib Prep kit (ABclonal) and sequenced in 2 × 150-nt paired-end mode on the Illumina NovaSeq platform at Annoroad.

TrimGalore (v0.6.6) (Martin, 2011) was used for adaptor trimming and quality filtering of raw mRNA-seq reads. Clean reads were aligned to the IRGSP-1.0 reference genome (Kawahara et al., 2013) via STAR (v2.7.10b) (Dobin et al., 2013). Uniquely mapped BAM files were analyzed using featureCounts (v2.0.1) (Liao et al., 2014) to quantify relative mRNA expression levels.

Immunoblot analysis

Immunoblot analysis was performed as previously described (Wang et al., 2018). Input, immunoprecipitate, and flow-through fractions from Ribo-seq were mixed with SDS loading buffer (80 mM Tris-HCl pH 6.8, 2% SDS, 10% glycerol, 0.1 M DTT, and 0.005% bromophenol blue) and heated at 95°C for 5 min. Proteins in the supernatant were subjected to immunoblotting. Membranes were probed with a primary antibody against FLAG (ABclonal, cat# AE005, IB 1:5000), followed by an HRP (Horseradish Peroxidase)-conjugated goat anti-mouse IgG secondary antibody (Thermo Fisher, cat# 31431).

Transcriptional arrest assay for transcriptome-wide RNA decay

A transcriptional arrest assay was performed as previously described, with minor modifications (Sorenson et al., 2018; Chantarachot et al., 2020). Rice roots were harvested, cut into small disks (~1 cm), and immersed in 10 mL ActD incubation buffer (15 mM sucrose, 1 mM PIPES pH 6.5, 1 mM KCl, 1 mM sodium citrate, and 50 μM ActD [MCE]). Initial (T_0) samples were frozen immediately after 5 min of vacuum infiltration (~12 psi). Remaining samples were subjected to two additional rounds of 5-min of vacuum infiltration. After three infiltration rounds, samples were incubated at 30°C with shaking at 250 rpm. Samples were collected at 15, 30, 60, and 120 min, then frozen in liquid nitrogen and ground into powder.

Transcriptome-wide RNA decay library construction and analysis

Total RNA was extracted from powdered samples (for the RNA decay assay) using TRIzol (Thermo Fisher) and treated with TURBO DNase

(Thermo Fisher). DNase-treated RNA was subjected to library construction using a Stranded mRNA-seq Lib Prep kit (ABclonal). Libraries were sequenced in 2 × 150-nt paired-end mode on the NovaSeq X Plus platform at Annoroad.

Raw RNA decay sequencing reads were processed by adaptor trimming and quality filtering using TrimGalore (v0.6.6) (Martin, 2011). Clean reads were aligned to the IRGSP-1.0 reference genome (Kawahara et al., 2013) via HISAT2 (v2.1.0) (Kim et al., 2015) with default parameters. Uniquely mapped reads were selected using "grep -E " ^@|NH:i:1" for downstream analyses. The featureCounts (v2.0.1) (Liao et al., 2014) package was used to obtain raw read counts for all annotated genes. The DESeq2 (v1.46.0) (Love et al., 2014) package was used for sample clustering and PCA.

Data normalization and modeling of mRNA decay were performed using the Bioconductor RNAdecay package (v1.26.0) (Reed et al., 2019). Seven stably expressed genes were used for data normalization: Os09g0535000, Os01g0658400, Os12g0507200, Os04g0667800, Os07g0597000, Os10g0320400, and Os01g0652600. To determine transcript decay rates, half-lives were calculated using the formula: $t_{1/2} = \ln(2)/\alpha$. An RNA decay heatmap was generated using ComplexHeatmap (v2.24.0) (Gu et al., 2016).

Identification of intronless genes

The number of introns in rice genes was determined based on genome annotation (GFF3 file). Genes containing zero introns were considered intronless and used for downstream analyses.

ΔG *prediction*

Watson–Crick base pairs corresponding to RSS mutations were computationally removed using RNAstructure (v6.4) to simulate single-stranded RSS caused by point mutations. Perturbed structures were converted to connectivity table (CT) format using the dot2ct utility in the RNAstructure software. Subsequently, ensemble free energy (ΔG) values were calculated using efn2 in RNAstructure with CT files as input.

Target-specific RNA half-life measurement

Roots of 15-day-old *OsIPS2*^{WT}_OE, *OsIPS2*^{MUT}_OE, and *OsIPS2*^{Re}_OE transgenic rice plants grown under normal conditions were harvested. Root tissues were cut into small disks (~1 cm) and immersed in 10 mL incubation buffer (15 mM sucrose, 1 mM PIPES pH 6.5, 1 mM KCl, 1 mM sodium citrate, and 1 mM cordycepin [Solarbio]). After 5 min of vacuum infiltration (~12 psi), initial (0 min) samples were collected and immediately frozen. Remaining samples were subjected to two additional 5-min rounds of vacuum infiltration. After three infiltration rounds, samples were incubated with shaking at RT. Tissues were collected 60 min after the first vacuum release.

Total RNA was extracted using TRIzol (Thermo Fisher) and treated with TURBO DNase (Thermo Fisher). DNase-treated RNA was subjected to reverse transcription; expression levels of exogenous *OsIPS2* were quantified by RT-qPCR. The stably expressed gene Os01g0658400 served as an internal control. Primer sequences are listed in Supplemental Table 6.

GO analysis

GO analysis was performed using AmiGO2 (Ashburner et al., 2000; Carbon et al., 2009; Aleksander et al., 2023).

Quantification and statistical analysis

Details of statistical analyses, including tests performed, sample sizes (*n*) for each measurement, and *p* values, are provided in figure legends and figures.

RESOURCE AVAILABILITY

Lead contact

Zhiye Wang (wangzhiye1@zju.edu.cn, College of Life Sciences, Zhejiang University).

Materials availability

The results of DMS-MaPseq, Ribo-seq, and transcriptome-wide RNA decay assays generated in this study are provided in Supplemental Tables 1–5. The raw sequencing data from the DMS-MaPseq, Ribo-seq, and transcriptome-wide RNA decay assays have been deposited in the Genome Sequence Archive (GSA) under accession number GSA: CRA025390. Plasmids and transgenic plants generated in this study are available from the corresponding author upon reasonable request.

FUNDING

This work was supported by grants from the National Key Research and Development Program of China (2021YFF1000402), the Natural Science Foundation of Zhejiang Province, China (LR24C150001), the National Natural Science Foundation of China (32170262), and the Fundamental Research Funds for the Central University (226-2024-00102) to Z.W.

ACKNOWLEDGMENTS

The authors declare no competing interests.

AUTHOR CONTRIBUTIONS

Z.W. conceived the project and designed the experiments; Q.J., R.G., J. Y., Z.H., and G.W. performed the experiments; Q.J. conducted the data analyses, with assistance from K.L. and W.D.; and Z.W. and Q.J. wrote the manuscript with input from all authors.

SUPPLEMENTAL INFORMATION

Supplemental information is available at *Plant Communications Online*.

Received: June 28, 2025
Revised: August 6, 2025
Accepted: December 15, 2025
Published: December 17, 2025

REFERENCES

Abel, S. (2017). Phosphate scouting by root tips. Curr. Opin. Plant Biol. **39**:168–177.

Ashburner, M., Ball, C.A., Blake, J.A., Botstein, D., Butler, H., Cherry, J.M., Davis, A.P., Dolinski, K., Dwight, S.S., Eppig, J.T., et al. (2000). Gene Ontology: tool for the unification of biology. Nat. Genet. **25**:25–29.

Bailey-Serres, J., Parker, J.E., Ainsworth, E.A., Oldroyd, G.E.D., and Schroeder, J.I. (2019). Genetic strategies for improving crop yields. Nature **575**:109–118.

Bariola, P.A., Howard, C.J., Taylor, C.B., Verburg, M.T., Jaglan, V.D., and Green, P.J. (1994). The Arabidopsis ribonuclease gene RNS1 is tightly controlled in response to phosphate limitation. Plant J. **6**:673–685.

Bazin, J., Baerenfaller, K., Gosai, S.J., Gregory, B.D., Crespi, M., and Bailey-Serres, J. (2017). Global analysis of ribosome-associated noncoding RNAs unveils new modes of translational regulation. Proc. Natl. Acad. Sci. USA **114**:E10018–E10027.

Beaudoin, J.D., Novoa, E.M., Vejnar, C.E., Yartseva, V., Takacs, C.M., Kellis, M., and Giraldez, A.J. (2018). Analyses of mRNA structure dynamics identify embryonic gene regulatory programs. Nat. Struct. Mol. Biol. **25**:677–686.

Bevilacqua, P.C., Ritchey, L.E., Su, Z., and Assmann, S.M. (2016). Genome-Wide Analysis of RNA Secondary Structure. Annu. Rev. Genet. **50**:235–266.

Bouain, N., Krouk, G., Lacombe, B., and Rouached, H. (2019). Getting to the Root of Plant Mineral Nutrition: Combinatorial Nutrient Stresses Reveal Emergent Properties. Trends Plant Sci. **24**:542–552.

Bushnell, B. (2014). BBMap: A Fast, Accurate, Splice-Aware Aligner. https://jgi.doe.gov/data-and-tools/bbtools/bb-tools-user-guide/bbmap-guide.

Carbon, S., Ireland, A., Mungall, C.J., Shu, S., Marshall, B., and Lewis, S.AmiGO Hub; Web Presence Working Group (2009). AmiGO: online access to ontology and annotation data. Bioinformatics **25**:288–289.

Chan, C.Y., Carmack, C.S., Long, D.D., Maliyekkel, A., Shao, Y., Roninson, I.B., and Ding, Y. (2009). A structural interpretation of the effect of GC-content on efficiency of RNA interference. BMC Bioinf. **10**:S33.

Chantarachot, T., Sorenson, R.S., Hummel, M., Ke, H., Kettenburg, A. T., Chen, D., Aiyetiwa, K., Dehesh, K., Eulgem, T., Sieburth, L.E., and Bailey-Serres, J. (2020). DHH1/DDX6-like RNA helicases maintain ephemeral half-lives of stress-response mRNAs. Nat. Plants **6**:675–685.

Chen, G., Hu, Q., Luo, L., Yang, T., Zhang, S., Hu, Y., Yu, L., and Xu, G. (2015a). Rice potassium transporter OsHAK1 is essential for maintaining potassium-mediated growth and functions in salt tolerance over low and high potassium concentration ranges. Plant Cell Environ. **38**:2747–2765.

Chen, G., Liu, C., Gao, Z., Zhang, Y., Jiang, H., Zhu, L., Ren, D., Yu, L., Xu, G., and Qian, Q. (2017). OsHAK1, a High-Affinity Potassium Transporter, Positively Regulates Responses to Drought Stress in Rice. Front. Plant Sci. **8**:1885.

Chen, J., Liu, Y., Ni, J., Wang, Y., Bai, Y., Shi, J., Gan, J., Wu, Z., and Wu, P. (2011). OsPHF1 Regulates the Plasma Membrane Localization of Low- and High-Affinity Inorganic Phosphate Transporters and Determines Inorganic Phosphate Uptake and Translocation in Rice. Plant Physiol. **157**:269–278.

Chen, J., Wang, Y., Wang, F., Yang, J., Gao, M., Li, C., Liu, Y., Liu, Y., Yamaji, N., Ma, J.F., et al. (2015b). The Rice CK2 Kinase Regulates Trafficking of Phosphate Transporters in Response to Phosphate Levels. Plant Cell **27**:711–723.

Chiou, T.J., Aung, K., Lin, S.I., Wu, C.C., Chiang, S.F., and Su, C.L. (2006). Regulation of phosphate homeostasis by microRNA in. Plant Cell **18**:412–421.

Cong, W.F., Suriyagoda, L.D.B., and Lambers, H. (2020). Tightening the Phosphorus Cycle through Phosphorus-Efficient Crop Genotypes. Trends Plant Sci. **25**:967–975.

Dai, X., Wang, Y., Yang, A., and Zhang, W.H. (2012). OsMYB2P-1, an R2R3 MYB Transcription Factor, Is Involved in the Regulation of Phosphate-Starvation Responses and Root Architecture in Rice. Plant Physiol. **159**:169–183.

Darty, K., Denise, A., and Ponty, Y. (2009). VARNA: Interactive drawing and editing of the RNA secondary structure. Bioinformatics **25**:1974–1975.

Deng, H., Cheema, J., Zhang, H., Woolfenden, H., Norris, M., Liu, Z., Liu, Q., Yang, X., Yang, M., Deng, X., et al. (2018). Rice In Vivo RNA Structurome Reveals RNA Secondary Structure Conservation and Divergence in Plants. Mol. Plant **11**:607–622.

Deng, S., Li, J., Du, Z., Wu, Z., Yang, J., Cai, H., Wu, G., Xu, F., Huang, Y., Wang, S., and Wang, C. (2022). Rice ACID PHOSPHATASE 1 regulates Pi stress adaptation by maintaining intracellular Pi homeostasis. Plant Cell Environ. **45**:191–205.

Ding, Y., Tang, Y., Kwok, C.K., Zhang, Y., Bevilacqua, P.C., and Assmann, S.M. (2014). genome-wide profiling of RNA secondary structure reveals novel regulatory features. Nature **505**:696–700.

Dissanayaka, D.M.S.B., Ghahremani, M., Siebers, M., Wasaki, J., and Plaxton, W.C. (2021). Recent insights into the metabolic adaptations of phosphorus-deprived plants. J. Exp. Bot. **72**:199–223.

Dobin, A., Davis, C.A., Schlesinger, F., Drenkow, J., Zaleski, C., Jha, S., Batut, P., Chaisson, M., and Gingeras, T.R. (2013). STAR: ultrafast universal RNA-seq aligner. Bioinformatics **29**:15–21.

Fahad, S., Adnan, M., Noor, M., Arif, M., Alam, M., Khan, I.A., Ullah, H., Wahid, F., Mian, I.A., Jamal, Y., et al. (2019). Chapter 1 - Major Constraints for Global Rice Production. In Advances in Rice Research for Abiotic Stress Tolerance, M. Hasanuzzaman, M. Fujita, K. Nahar, and J.K. Biswas, eds. (Woodhead Publishing), pp. 1–22.

Fan, X., Naz, M., Fan, X., Xuan, W., Miller, A.J., and Xu, G. (2017). Plant nitrate transporters: from gene function to application. J. Exp. Bot. **68**:2463–2475.

FAO (2018).RICE MARKET MONITOR. http://www.fao.org/3/I9243EN/i9243en.pdf.

Finkel, O.M., Salas-González, I., Castrillo, G., Spaepen, S., Law, T.F., Teixeira, P.J.P.L., Jones, C.D., and Dangl, J.L. (2019). The effects of soil phosphorus content on plant microbiota are driven by the plant phosphate starvation response. PLoS Biol. **17**:e3000534.

Franco-Zorrilla, J.M., Valli, A., Todesco, M., Mateos, I., Puga, M.I., Rubio-Somoza, I., Leyva, A., Weigel, D., García, J.A., and Paz-Ares, J. (2007). Target mimicry provides a new mechanism for regulation of microRNA activity. Nat. Genet. **39**:1033–1037.

Gao, W., Lu, L., Qiu, W., Wang, C., and Shou, H. (2017). Encodes a Major Purple Acid Phosphatase and Regulates Phosphate Remobilization in Rice. Plant Cell Physiol. **58**:885–892.

Gene Ontology Consortium, Aleksander, S.A., Balhoff, J., Carbon, S., Cherry, J.M., Drabkin, H.J., Ebert, D., Feuermann, M., Gaudet, P., Harris, N.L., et al. (2023). The Gene Ontology knowledgebase in 2023. Genetics **224**:iyad031.

Gho, Y.S., Choi, H., Moon, S., Song, M.Y., Park, H.E., Kim, D.H., Ha, S. H., and Jung, K.H. (2020). Phosphate-Starvation-Inducible S-Like RNase Genes in Rice Are Involved in Phosphate Source Recycling by RNA Decay. Front. Plant Sci. **11**:585561.

Gordon, A., and Hannon, G. (2010). Fastx-toolkit. http://hannonlab.cshl.edu/fastx_toolkit.

Gu, Z., Eils, R., and Schlesner, M. (2016). Complex heatmaps reveal patterns and correlations in multidimensional genomic data. Bioinformatics **32**:2847–2849. https://doi.org/10.1093/bioinformatics/btw313.

Guo, M., Ruan, W., Li, C., Huang, F., Zeng, M., Liu, Y., Yu, Y., Ding, X., Wu, Y., Wu, Z., et al. (2015). Integrative Comparison of the Role of the PHOSPHATE RESPONSE1 Subfamily in Phosphate Signaling and Homeostasis in Rice. Plant Physiol. **168**:1762–1776.

Gutiérrez-Alanis, D., Ojeda-Rivera, J.O., Yong-Villalobos, L., Cárdenas-Torres, L., and Herrera-Estrella, L. (2018). Adaptation to Phosphate Scarcity: Tips from Arabidopsis Roots. Trends Plant Sci. **23**:721–730.

Han, W.Y., Hou, B.H., Lee, W.C., Chan, T.C., Lin, T.H., and Chen, H.M. (2023). Arabidopsis mRNA decay landscape shaped by XRN 5′-3′ exoribonucleases. Plant J. **114**:895–913.

He, Q., Lu, H., Guo, H., Wang, Y., Zhao, P., Li, Y., Wang, F., Xu, J., Mo, X., and Mao, C. (2021). OsbHLH6 interacts with OsSPX4 and regulates the phosphate starvation response in rice. Plant J. **105**:649–667.

Hou, X.L., Wu, P., Jiao, F.C., Jia, Q.J., Chen, H.M., Yu, J., Song, X.W., and Yi, K.K. (2005). Regulation of the expression of OsIPS1 and OsIPS2 in rice via systemic and local Pi signalling and hormones. Plant Cell Environ. **28**:353–364.

Hsu, P.Y., Calviello, L., Wu, H.Y.L., Li, F.W., Rothfels, C.J., Ohler, U., and Benfey, P.N. (2016). Super-resolution ribosome profiling reveals unannotated translation events in. Proc. Natl. Acad. Sci. USA **113**:E7126–E7135.

Ingolia, N.T., Ghaemmaghami, S., Newman, J.R.S., and Weissman, J. S. (2009). Genome-wide analysis in vivo of translation with nucleotide resolution using ribosome profiling. Science **324**:218–223.

Jabnoune, M., Secco, D., Lecampion, C., Robaglia, C., Shu, Q., and Poirier, Y. (2013). A Rice cis-Natural Antisense RNA Acts as a Translational Enhancer for Its Cognate mRNA and Contributes to Phosphate Homeostasis and Plant Fitness. Plant Cell **25**:4166–4182.

Jia, H., Ren, H., Gu, M., Zhao, J., Sun, S., Zhang, X., Chen, J., Wu, P., and Xu, G. (2011). The Phosphate Transporter Gene OsPht1;4 Is Involved in Phosphate Homeostasis in Rice. Plant Physiol. **156**:1164–1175.

Jin, Q., Zhang, L., Hu, S., Wei, G., and Wang, Z. (2022). Probing in vivo RNA Structure With Optimized DMS-MaPseq in Rice. Front. Plant Sci. **13**:869267.

Jung, J.Y., Ried, M.K., Hothorn, M., and Poirier, Y. (2018). Control of plant phosphate homeostasis by inositol pyrophosphates and the SPX domain. Curr. Opin. Biotechnol. **49**:156–162.

Juntawong, P., Hummel, M., Bazin, J., and Bailey-Serres, J. (2015). Ribosome profiling: a tool for quantitative evaluation of dynamics in mRNA translation. Methods Mol. Biol. **1284**:139–173.

Kawahara, Y., de la Bastide, M., Hamilton, J.P., Kanamori, H., McCombie, W.R., Ouyang, S., Schwartz, D.C., Tanaka, T., Wu, J., Zhou, S., et al. (2013). Improvement of the Oryza sativa Nipponbare reference genome using next generation sequence and optical map data. Rice **6**:4.

Kim, D., Langmead, B., and Salzberg, S.L. (2015). HISAT: a fast spliced aligner with low memory requirements. Nat. Methods **12**:357–360. https://doi.org/10.1038/nmeth.3317.

Kim, D., Pertea, G., Trapnell, C., Pimentel, H., Kelley, R., and Salzberg, S.L. (2013). TopHat2: accurate alignment of transcriptomes in the presence of insertions, deletions and gene fusions. Genome Biol. **14**:R36.

Krummel, D.A.P., Oubridge, C., Leung, A., Li, J., and Nagai, K. (2009). Crystal Structure of Human Spliceosomal U1 snRNP at 5.5 Å Resolution. Acta Crystallogr. A **65**:S14.

Lambers, H. (2022). Phosphorus Acquisition and Utilization in Plants. Annu. Rev. Plant Biol. **73**:17–42.

Lan, T.C.T., Allan, M.F., Malsick, L.E., Woo, J.Z., Zhu, C., Zhang, F., Khandwala, S., Nyeo, S.S.Y., Sun, Y., Guo, J.U., et al. (2022). Secondary structural ensembles of the SARS-CoV-2 RNA genome in infected cells. Nat. Commun. **13**:1128.

Lauria, F., Tebaldi, T., Bernabò, P., Groen, E.J.N., Gillingwater, T.H., and Viero, G. (2018). riboWaltz: Optimization of ribosome P-site positioning in ribosome profiling data. PLoS Comput. Biol. **14**: e1006169.

Li, H., Handsaker, B., Wysoker, A., Fennell, T., Ruan, J., Homer, N., Marth, G., Abecasis, G., and Durbin, R.; 1000 Genome Project Data Processing Subgroup (2009). The Sequence Alignment/Map format and SAMtools. Bioinformatics **25**:2078–2079.

Li, X., Zhong, S., Li, C., Yan, X., Zhu, J., Li, Y., Wang, Z., Peng, X., and Zhang, X. (2024). RNA helicase Brr2a promotes miRNA biogenesis by properly remodelling secondary structure of pri-miRNAs. Nat. Plants **10**:1532–1547.

Liao, Y., Smyth, G.K., and Shi, W. (2014). featureCounts: an efficient general purpose program for assigning sequence reads to genomic features. Bioinformatics **30**:923–930.

Lin, S.I., Chiang, S.F., Lin, W.Y., Chen, J.W., Tseng, C.Y., Wu, P.C., and Chiou, T.J. (2008). Regulatory network of microRNA399 and PHO2 by systemic signaling. Plant Physiol. **147**:732–746.

Liu, F., Wang, Z., Ren, H., Shen, C., Li, Y., Ling, H.Q., Wu, C., Lian, X., and Wu, P. (2010). OsSPX1 suppresses the function of OsPHR2 in the regulation of expression of OsPT2 and phosphate homeostasis in shoots of rice. Plant J. **62**:508–517.

Liu, K.H., Liu, M., Lin, Z., et al. (2022a). NIN-like protein 7 transcription factor is a plant nitrate sensor. Science **377**:1419–1425.

Liu, X., Hu, B., and Chu, C. (2022b). Nitrogen assimilation in plants: current status and future prospects. J Genet. Genom. **49**:394–404.

Liu, Z., Liu, Q., Yang, X., Zhang, Y., Norris, M., Chen, X., Cheema, J., Zhang, H., and Ding, Y. (2021). In vivo nuclear RNA structurome reveals RNA-structure regulation of mRNA processing in plants. Genome Biol. **22**:11.

López-Arredondo, D.L., Leyva-González, M.A., González-Morales, S. I., López-Bucio, J., and Herrera-Estrella, L. (2014). Phosphate Nutrition: Improving Low-Phosphate Tolerance in Crops. Annu. Rev. Plant Biol. **65**:95–123.

Love, M.I., Huber, W., and Anders, S. (2014). Moderated estimation of fold change and dispersion for RNA-seq data with DESeq2. Genome Biol. **15**:550.

Martin, M. (2011). Cutadapt removes adapter sequences from high-throughput sequencing reads. EMBnet. J. **17**:10.

Megel, C., Hummel, G., Lalande, S., Ubrig, E., Cognat, V., Morelle, G., Salinas-Giegé, T., Duchêne, A.M., and Maréchal-Drouard, L. (2019). Plant RNases T2, but not Dicer-like proteins, are major players of tRNA-derived fragments biogenesis. Nucl. Acids Res. **47**:941–952.

Narsai, R., Howell, K.A., Millar, A.H., O'Toole, N., Small, I., and Whelan, J. (2007). Genome-wide analysis of mRNA decay rates and their determinants in. Plant Cell **19**:3418–3436.

Pan, W., Wu, Y., and Xie, Q. (2019). Regulation of Ubiquitination Is Central to the Phosphate Starvation Response. Trends Plant Sci. **24**:755–769.

Pant, B.D., Buhtz, A., Kehr, J., and Scheible, W.R. (2008). MicroRNA399 is a long-distance signal for the regulation of plant phosphate homeostasis. Plant J. **53**:731–738.

Pant, B.D., Pant, P., Erban, A., Huhman, D., Kopka, J., and Scheible, W.R. (2015). Identification of primary and secondary metabolites with phosphorus status-dependent abundance in Arabidopsis, and of the transcription factor PHR1 as a major regulator of metabolic changes during phosphorus limitation. Plant Cell Environ. **38**:172–187.

Paz-Ares, J., Puga, M.I., Rojas-Triana, M., Martinez-Hevia, I., Diaz, S., Poza-Carrión, C., Miñambres, M., and Leyva, A. (2022). Plant adaptation to low phosphorus availability: Core signaling, crosstalks, and applied implications. Mol. Plant **15**:104–124.

Péret, B., Desnos, T., Jost, R., Kanno, S., Berkowitz, O., and Nussaume, L. (2014). Root Architecture Responses: In Search of Phosphate. Plant Physiol. **166**:1713–1723.

Puga, M.I., Rojas-Triana, M., de Lorenzo, L., Leyva, A., Rubio, V., and Paz-Ares, J. (2017). Novel signals in the regulation of Pi starvation responses in plants: facts and promises. Curr. Opin. Plant Biol. **39**:40–49.

Puga, M.I., Mateos, I., Charukesi, R., Wang, Z., Franco-Zorrilla, J.M., de Lorenzo, L., Irigoyen, M.L., Masiero, S., Bustos, R., Rodríguez, J., et al. (2014). SPX1 is a phosphate-dependent inhibitor of PHOSPHATE STARVATION RESPONSE 1. Proc. Natl. Acad. Sci. USA **111**:14947–14952.

Quinlan, A.R., and Hall, I.M. (2010). BEDTools: a flexible suite of utilities for comparing genomic features. Bioinformatics **26**:841–842.

Reed, S., Katrina, J., Frederick, A., and Leslie, S. (2019). RNAdecay: Maximum Likelihood Decay Modeling of RNA Degradation Data. R package version 1.26.0.

Reis, R.S., Deforges, J., Schmidt, R.R., Schippers, J.H.M., and Poirier, Y. (2021). An antisense noncoding RNA enhances translation via

localized structural rearrangements of its cognate mRNA. Plant Cell **33**:1381–1397.

Reuter, J.S., and Mathews, D.H. (2010). RNAstructure: software for RNA secondary structure prediction and analysis. BMC Bioinf. **11**:129.

Rouskin, S., Zubradt, M., Washietl, S., Kellis, M., and Weissman, J.S. (2014). Genome-wide probing of RNA structure reveals active unfolding of mRNA structures. Nature **505**:701–705.

Ruan, W., Guo, M., Xu, L., Wang, X., Zhao, H., Wang, J., and Yi, K. (2018). An SPX-RLI1 Module Regulates Leaf Inclination in Response to Phosphate Availability in Rice. Plant Cell **30**:853–870.

Rubio, V., Linhares, F., Solano, R., Martín, A.C., Iglesias, J., Leyva, A., and Paz-Ares, J. (2001). A conserved MYB transcription factor involved in phosphate starvation signaling both in vascular plants and in unicellular algae. Gene Dev. **15**:2122–2133.

Schachtman, D.P., and Shin, R. (2007). Nutrient sensing and signaling: NPKS. Annu. Rev. Plant Biol. **58**:47–69.

Sen, S., Chakraborty, R., and Kalita, P. (2020). Rice - not just a staple food: A comprehensive review on its phytochemicals and therapeutic potential. Trends Food Sci. Tech. **97**:265–285.

Shen, C., Wang, S., Zhang, S., Xu, Y., Qian, Q., Qi, Y., and Jiang, D.A. (2013). OsARF16, a transcription factor, is required for auxin and phosphate starvation response in rice (Oryza sativa L.). Plant Cell Environ. **36**:607–620.

Sorenson, R.S., Deshotel, M.J., Johnson, K., Adler, F.R., and Sieburth, L.E. (2018). mRNA decay landscape arises from specialized RNA decay substrates, decapping- mediated feedback, and redundancy. Proc. Natl. Acad. Sci. USA **115**:E1485–E1494.

Spitale, R.C., Flynn, R.A., Zhang, Q.C., Crisalli, P., Lee, B., Jung, J.W., Kuchelmeister, H.Y., Batista, P.J., Torre, E.A., Kool, E.T., and Chang, H.Y. (2015). Structural imprints in vivo decode RNA regulatory mechanisms. Nature **527**:264.

Su, Z., Tang, Y., Ritchey, L.E., Tack, D.C., Zhu, M., Bevilacqua, P.C., and Assmann, S.M. (2018). Genome-wide RNA structurome reprogramming by acute heat shock globally regulates mRNA abundance. Proc. Natl. Acad. Sci. USA **115**:12170–12175.

Tack, D.C., Su, Z., Yu, Y., Bevilacqua, P.C., and Assmann, S.M. (2020). Tissue-specific changes in the RNA structurome mediate salinity response in Arabidopsis. RNA **26**:492–511.

Thiel, B.C., Beckmann, I.K., Kerpedjiev, P., and Hofacker, I.L. (2019). 3D based on 2D: Calculating helix angles and stacking patterns using forgi 2.0, an RNA Python library centered on secondary structure elements. F1000Res. **8**. ISCB Comm.

Ticconi, C.A., Lucero, R.D., Sakhonwasee, S., Adamson, A.W., Creff, A., Nussaume, L., Desnos, T., and Abel, S. (2009). ER-resident proteins PDR2 and LPR1 mediate the developmental response of root meristems to phosphate availability. Proc. Natl Acad Sci USA **106**:14174–14179.

Toki, S., Hara, N., Ono, K., Onodera, H., Tagiri, A., Oka, S., and Tanaka, H. (2006). Early infection of scutellum tissue with Agrobacterium allows high-speed transformation of rice. Plant J. **47**:969–976.

Tomezsko, P.J., Corbin, V.D.A., Gupta, P., Swaminathan, H., Glasgow, M., Persad, S., Edwards, M.D., Mcintosh, L., Papenfuss, A.T., Emery, A., et al. (2020). Determination of RNA structural diversity and its role in HIV-1 RNA splicing. Nature **582**:438–442.

Vance, C.P. (2001). Symbiotic nitrogen fixation and phosphorus acquisition. Plant nutrition in a world of declining renewable resources. Plant Physiol. **127**:390–397.

Vandivier, L.E., Anderson, S.J., Foley, S.W., and Gregory, B.D. (2016). The Conservation and Function of RNA Secondary Structure in Plants. Annu. Rev. Plant Biol. **67**:463–488.

Versaw, W.K., and Garcia, L.R. (2017). Intracellular transport and compartmentation of phosphate in plants. Curr. Opin. Plant Biol. **39**:25–30.

Wang, X.W., Liu, C.X., Chen, L.L., and Zhang, Q.C. (2021). RNA structure probing uncovers RNA structure-dependent biological functions. Nat. Chem. Biol. **17**:755–766.

Wang, Z., Wang, M., Wang, T., Zhang, Y., and Zhang, X. (2019). Genome-wide probing RNA structure with the modified DMS-MaPseq in Arabidopsis. Methods **155**:30–40.

Wang, Z., Ma, Z., Castillo-González, C., Sun, D., Li, Y., Yu, B., Zhao, B., Li, P., and Zhang, X. (2018). SWI2/SNF2 ATPase CHR2 remodels pri-miRNAs via Serrate to impede miRNA production. Nature **557**:516–521.

Wang, Z., Ruan, W., Shi, J., Zhang, L., Xiang, D., Yang, C., Li, C., Wu, Z., Liu, Y., Yu, Y., et al. (2014). Rice SPX1 and SPX2 inhibit phosphate starvation responses through interacting with PHR2 in a phosphate-dependent manner. Proc. Natl. Acad. Sci. USA **111**:14953–14958.

Weng, X., Gong, J., Chen, Y., Wu, T., Wang, F., Yang, S., Yuan, Y., Luo, G., Chen, K., Hu, L., et al. (2020). Keth-seq for transcriptome-wide RNA structure mapping. Nat. Chem. Biol. **16**:489–492.

Wu, H., Yu, H., Zhang, Y., Yang, B., Sun, W., Ren, L., Li, Y., Li, Q., Liu, B., Ding, Y., and Zhang, H. (2024). Unveiling RNA structure-mediated regulations of RNA stability in wheat. Nat. Commun. **15**:10042.

Xiang, Y., Huang, W., Tan, L., Chen, T., He, Y., Irving, P.S., Weeks, K. M., Zhang, Q.C., and Dong, X. (2023). Pervasive downstream RNA hairpins dynamically dictate start-codon selection. Nature **621**:423–430.

Xu, B., Zhu, Y., Cao, C., Chen, H., Jin, Q., Li, G., Ma, J., Yang, S.L., Zhao, J., Zhu, J., et al. (2022). Recent advances in RNA structurome. Sci. China Life Sci. **65**:1285–1324.

Yamaji, N., Takemoto, Y., Miyaji, T., Mitani-Ueno, N., Yoshida, K.T., and Ma, J.F. (2017). Reducing phosphorus accumulation in rice grains with an impaired transporter in the node. Nature **541**:136.

Yan, X., Li, C., Liu, K., Zhang, T., Xu, Q., Li, X., Zhu, J., Wang, Z., Yusuf, A., Cao, S., et al. (2024). Parallel degradome-seq and DMS-MaPseq substantially revise the miRNA biogenesis atlas in. Nat. Plants **10**:1126–1143.

Yang, M., Zhu, P., Cheema, J., Bloomer, R., Mikulski, P., Liu, Q., Zhang, Y., Dean, C., and Ding, Y. (2022a). In vivo single-molecule analysis reveals COOLAIR RNA structural diversity. Nature **609**:394–399.

Yang, M., Woolfenden, H.C., Zhang, Y., Fang, X., Liu, Q., Vigh, M.L., Cheema, J., Yang, X., Norris, M., Yu, S., et al. (2020a). Intact RNA structurome reveals mRNA structure-mediated regulation of miRNA cleavage. Nucl. Acids Res. **48**:8767–8781.

Yang, S.Y., Lin, W.Y., Hsiao, Y.M., and Chiou, T.J. (2024). Milestones in understanding transport, sensing, and signaling of the plant nutrient phosphorus. Plant Cell **36**:1504–1523.

Yang, X., Cheema, J., Zhang, Y., Deng, H., Duncan, S., Umar, M.I., Zhao, J., Liu, Q., Cao, X., Kwok, C.K., and Ding, Y. (2020b). RNA G-quadruplex structures exist and function in vivo in plants. Genome Biol. **21**:226.

Yang, X., Yu, H., Duncan, S., Zhang, Y., Cheema, J., Liu, H., Benjamin Miller, J., Zhang, J., Kwok, C.K., Zhang, H., and Ding, Y. (2022b). RNA G-quadruplex structure contributes to cold adaptation in plants. Nat. Commun. **13**:6224.

Yang, X., Yu, H., Sun, W., Ding, L., Li, J., Cheema, J., Ramirez-Gonzalez, R., Zhao, X., Martín, A.C., Lu, F., et al. (2021). Wheat in vivo RNA structure landscape reveals a prevalent role of RNA structure in modulating translational subgenome expression asymmetry. Genome Biol. **22**:326.

Yang, Z., Yang, J., Wang, Y., Wang, F., Mao, W., He, Q., Xu, J., Wu, Z., and Mao, C. (2020c). PROTEIN PHOSPHATASE95 Regulates Phosphate Homeostasis by Affecting Phosphate Transporter Trafficking in Rice. Plant Cell **32**:740–757.

Yoshida, S., Forno, D.A., Cock, J.H., and Gomez, K.A. (1976). Laboratory Manual for Physiological Studies of Rice, 3rd Edition (International Rice Research Institute).

Zhang, H., and Ding, Y. (2025). RNA Structure: Function and Application in Plant Biology. Annu. Rev. Plant Biol. **10**:1146.

Zhang, T., Li, C., Zhu, J., Li, Y., Wang, Z., Tong, C.Y., Xi, Y., Han, Y., Koiwa, H., Peng, X., and Zhang, X. (2024). Structured 3' UTRs destabilize mRNAs in plants. Genome Biol. **25**:54.

Zhang, X., Spiegel, J., Martínez Cuesta, S., Adhikari, S., and Balasubramanian, S. (2021). Chemical profiling of DNA G-quadruplex-interacting proteins in live cells. Nat. Chem. **13**:626–633.

Zhang, Y., Yang, M., Duncan, S., Yang, X., Abdelhamid, M.A.S., Huang, L., Zhang, H., Benfey, P.N., Waller, Z.A.E., and Ding, Y. (2019). G-quadruplex structures trigger RNA phase separation. Nucl. Acids Res. **47**:11746–11754.

Zhang, Z.S., Xia, J.Q., Alfatih, A., Song, Y., Huang, Y.J., Sun, L.Q., Wan, G.Y., Wang, S.M., Wang, Y.P., Hu, B.H., et al. (2022). Rice MIN-LIKE PROTEIN 3 modulates nitrogen use efficiency and grain yield under nitrate-sufficient conditions. Plant Cell Environ. **45**:1520–1536.

Zhao, X.Y., Wang, H.Q., Shi, W., Zhang, W.W., and Zhao, F.J. (2025). The Respiratory Burst Oxidase Homologue OsRBOHE is crucial for root hair formation, drought resistance and tillering in rice. Plant Cell Environ. **48**:65–80.

Zhou, J., Jiao, F., Wu, Z., Li, Y., Wang, X., He, X., Zhong, W., and Wu, P. (2008). OsPHR2 is involved in phosphate-starvation signaling and excessive phosphate accumulation in shoots of plants. Plant Physiol. **146**:1673–1686.

Zhu, J., Lau, K., Puschmann, R., Harmel, R.K., Zhang, Y., Pries, V., Gaugler, P., Broger, L., Dutta, A.K., Jessen, H.J., et al. (2019). Two bifunctional inositol pyrophosphate kinases/phosphatases control plant phosphate homeostasis. eLife **8**:e43582.

Zhu, J., Li, C., Peng, X., and Zhang, X. (2021). RNA architecture influences plant biology. J. Exp. Bot. **72**:4144–4160.

Zubradt, M., Gupta, P., Persad, S., Lambowitz, A.M., Weissman, J.S., and Rouskin, S. (2017). DMS-MaPseq for genome-wide or targeted RNA structure probing. Nat. Methods **14**:75–82.

Plant Communications, Volume 7

Supplemental information

Unfolding of RNA secondary structure impairs RNA stability to fine-tune phosphate starvation responses in rice roots

Qiongli Jin, Ruiren Gao, Jiakai Yao, Zhengwei Huang, Kai Liu, Guangbo Wei, Weiguo Dong, and Zhiye Wang

Supplemental Information

Unfolding of RNA secondary structure impairs RNA stability to fine-tune phosphate starvation responses in rice roots

Qiongli Jin[1], Ruiren Gao[1,2], Jiakai Yao[1,2], Zhengwei Huang[1], Kai Liu[1], Guangbo Wei[1], Weiguo Dong[1], Zhiye Wang[1,*]

Affiliations:

[1]State Key Laboratory of Plant Environmental Resilience, College of Life Sciences, Zhejiang University, Hangzhou, Zhejiang, 310058, China.

[2]These authors contributed equally: Ruiren Gao, Jiakai Yao.

* Correspondence: wangzhiye1@zju.edu.cn (Z.W.)

Supplemental note

Generating a high-quality, deep-coverage RNA structurome from rice roots in response to N deficiency stress

Rice roots were sampled after a 5-day N deficiency treatment (hereafter, –N), accompanied by control (normal) samples that were the same controls used for the –P samples. Three biological replicates were performed for DMS-treated samples and one biological replicate for the non-DMS-treated sample. Approximately 491–595 million clean reads were generated for each DMS-treated sample. More than 87.6% of the clean reads were uniquely mapped to the rice cv. Nipponbare reference genome IRGSP 1.0 (Supplemental Table 1).

Both clustering and PCA results exhibited high reproducibility among DMS-treated biological replicates of –N samples (Supplemental Figure 1A and 1B). The significantly reduced total N levels and dramatically increased expression of the –N response gene *OsNRT2.3* validated the success of –N treatment (Supplemental Figure 1C and 1D). Moreover, the mismatch percentages of A and C, but not G and U, were much higher in the three DMS-treated samples than in the non-DMS-treated control, showing the high signal-to-noise ratio of our data (Supplemental Figure 1E). Similar to published RNA structure data across the mRNAs (Ding *et al.*, 2014; Deng *et al.*, 2018), a 3-nt periodicity across the CDS and high DMS activity in the vicinity of the start codon were observed in our –N RNA structurome data, confirming the high reliability of the data (Supplemental Figure 4A).

Next, the RNA regions with high-conf. RNA structural information in the –N RNA structurome data were identified (see Methods). Each DMS-treated –N biological replicate obtained an average of 94,581 high-conf. RSS information windows associated with ~10,000 genes, accounting for ~42% of expressed genes (Supplemental Figure 2A). Notably, the Gini indexes of high-conf. RSS information windows exhibited high reproducibility among the three DMS-treated biological replicates (Supplemental Figure 4B). Therefore, these three biological samples were merged, yielding 187,406 high-conf. RSS information windows (corresponding to 16,148 genes) accounting for 60.80% of

expressed genes under –N conditions (Supplemental Figure 2A). Each covered gene harbored an average of 11.61 high-conf. RSS information 100-nt windows (Supplemental Figure 2A). These high-conf. RSS information windows were distributed across the whole rice genome and were associated with gene density (Supplemental Figure 4C). These windows mainly covered mRNAs, followed by small nucleolar RNAs, snRNAs, lncRNAs, rRNAs, tRNAs, and primary miRNAs (Supplemental Figure 4D).

These results confirm the high quality and deep coverage of our –N RNA structurome data.

The features of –N induced RSS-unfolding RNA regions

To characterize the sequence and structural features of the –N induced RSS-unfolding RNA regions, we divided –N induced RSS-unfolding windows into two subsets: –P/–N common RSS-unfolding windows (894 of total 1533, defined as –P&–N RSS-unfolding windows) and –N-unique RSS-unfolding windows (639 of total 1533) (Figure 2G). We then conduced an in-depth analysis of these two sets of RSS windows, including assessments of GC content, Gini index alterations under different nutrient conditions and GO enrichment analysis.

The results revealed that both –P&–N and –N-unique unfolding windows had a significantly lower GC content than the total windows (Supplemental Figure 5A). Furthermore, under –P and –N conditions, the Gini index of –P&–N RSS-unfolding windows was lower than that of the total RSS windows; under normal conditions, however, it was higher than that of total RSS windows, and comparable under ReP conditions (Supplemental Figure 5B). A similar trend in Gini index alteration was observed in –N-unique RSS-unfolding windows: their Gini index was lower than that of total RSS windows under –N conditions, but the opposite was true under other conditions (Supplemental Figure 5C). These results reflected dramatic RSS unfolding in both –P&–N and –N-unique RSS-unfolding RSS regions when shifting from normal to –P and/or –N conditions, demonstrating the structural flexibility of RSS in these regions.

We further performed GO enrichment analysis to investigate the functions of transcripts with –P&–N or –N-unique RSS-unfolding transcripts. Several enriched pathways were shared between –P&–N and –N-unique RSS-unfolding transcripts, such as lipid metabolism, vesicle-mediated transport, and protein transport (Supplemental Figure 5D and 5E). These shared pathways were also enriched in PSI RSS-unfolding transcripts (Supplemental Figure 6D). Of note, the amide biosynthetic process was specifically enriched in –N-unique RSS-unfolding transcripts, but not in either PSI or –P&–N RSS-unfolding transcripts (Supplemental Figure 5E). As amide biosynthesis is a critical pathway in plant N assimilation and metabolism (Liu et al., 2022), this GO result highlights the specificity of –N-unique RSS-unfolding.

Comparing the above results of –N induced RSS-unfolding analysis (Supplemental Figure 5) with the PSI RSS-unfolding analysis results (Figure 3; Supplemental Figure 6), RSS-unfolding induced by –P and –N stresses exhibited both commonalities and distinctions. On one hand, RNA regions with RSS-unfolding induced by –P and –N stresses showed low GC content, indicating that RNA structures flexibly respond to these stresses. In addition, transcripts with –P and –N-induced RSS-unfolding shared some pathways, confirming cross-talk between phosphate and nitrogen metabolism and stress responses (Paz-Ares et al., 2022). On the other hand, GO analysis also identified specific enriched pathways in PSI and –N-unique RSS-unfolding transcripts, suggesting that RSS-unfolding plays certain regulatory roles in response to different stresses.

Supplemental Figures

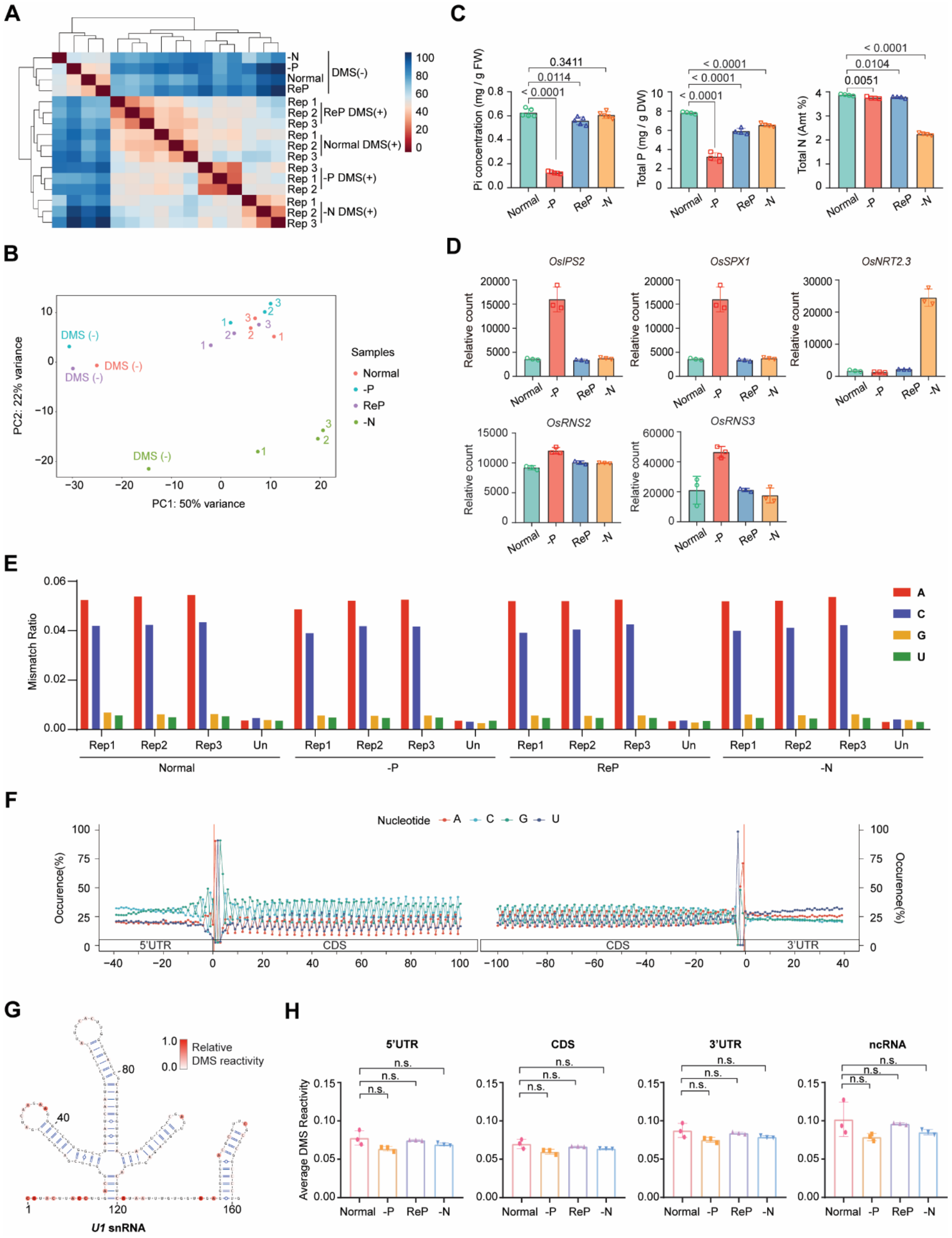

Supplemental Figure 1. High quality and reproducibility of the *in vivo* RSS data.

(A and B) Clustering **(A)** and PCA **(B)** showing the high reproducibility across three biological repeats of DMS-MaPseq from each sample.

(C) The Pi, total P, and total N concentrations in normal, –P, ReP, and –N samples. The *P* values were calculated using an unpaired two-tailed Student's *t*-test.

(D) The expression of the PSI genes *OsIPS2*, *OsSPX1*, *OsRNS2*, *OsRNS3*, and the –N-induced gene *OsNRT2.3* in normal, –P, ReP, and –N samples.

(E) As and Cs exhibit a higher mismatch ratio than Gs and Us in DMS-treated but not untreated (Un) samples, indicating a high signal-to-noise ratio in DMS-MaPseq.

(F) Average of AUGC content of all mRNAs.

(G) Predicted secondary structure of the *U1-5* snRNA modeled according to DMS-MaPseq data; this structure is consistent with the published crystal structure (Krummel et al., 2009). Color-coded A and C residues display different radiometric DMS signals.

(H) Bar diagrams revealing a lack of significant difference in the average DMS reactivity of 5′ UTRs, CDS regions, 3′ UTRs, or lncRNAs among normal, –P, ReP, and –N samples. The *P* values were calculated using an unpaired two-tailed Student's *t*-test. n.s., no significant.

–P, Pi deficiency; ReP, Pi-refeeding following Pi deficiency; –N, nitrogen deficiency.

A

DMS(+) Sample name	Expressed genes	High Conf. 100nt RSS Windows	Covered Genes	Covered genes/ expressed genes	No. of RSS windows/ Covered genes
Normal Rep1	23,584	94,217	10,173	43.14%	9.26
Normal Rep2	24,977	115,275	11,917	47.71%	9.67
Normal Rep3	23,280	77,907	8,982	38.58%	8.67
**Normal merge**	**26,854**	**190,350**	**16,412**	**61.12%**	**11.60**
-P Rep1	23,685	95,108	9,869	41.67%	9.64
-P Rep2	23,714	93,976	9,980	42.08%	9.42
-P Rep3	24,398	105,611	10,995	45.07%	9.61
**-P merge**	**26,779**	**193,774**	**16,288**	**60.82%**	**11.90**
ReP Rep1	24,518	107,824	11,235	45.82%	9.60
ReP Rep2	24,128	101,692	10,584	43.87%	9.61
ReP Rep3	24,190	103,895	10,899	45.06%	9.53
**ReP merge**	**27,026**	**194,316**	**16,617**	**61.49%**	**11.69**
-N Rep1	23,944	100,981	10,518	43.93%	9.60
-N Rep2	23,687	92,849	10,014	42.28%	9.27
-N Rep3	23,612	89,914	9,992	42.32%	9.00
**-N merge**	**26,559**	**187,406**	**16,148**	**60.80%**	**11.61**

B

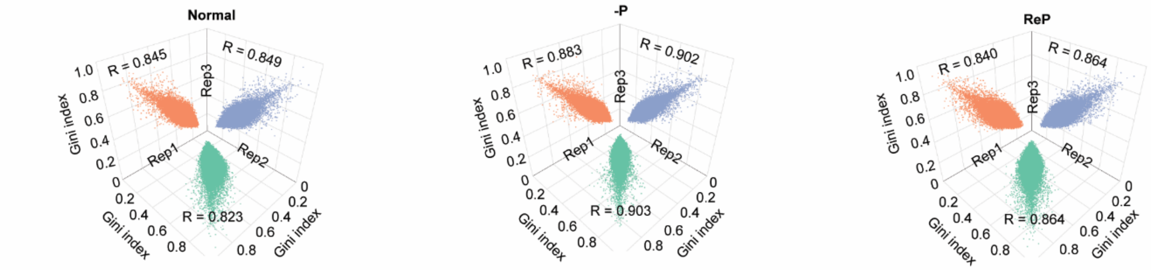

Supplemental Figure 2. Deep-coverage of the *in vivo* RSS data.

(A) Summary of the number of windows and genes with high-conf. 100-nt RSS windows in each sample, indicating that the RNA structurome data provide reliable RSS information for ~60% of all expressed genes. RSS, RNA secondary structure.

(B) 3D scatterplots showing the high reproducibility of Gini index values among three biological repeats in normal, –P, and ReP samples. *R* is the Pearson's correlation coefficient.

–P, Pi deficiency; ReP, Pi-refeeding following Pi deficiency; –N, nitrogen deficiency.

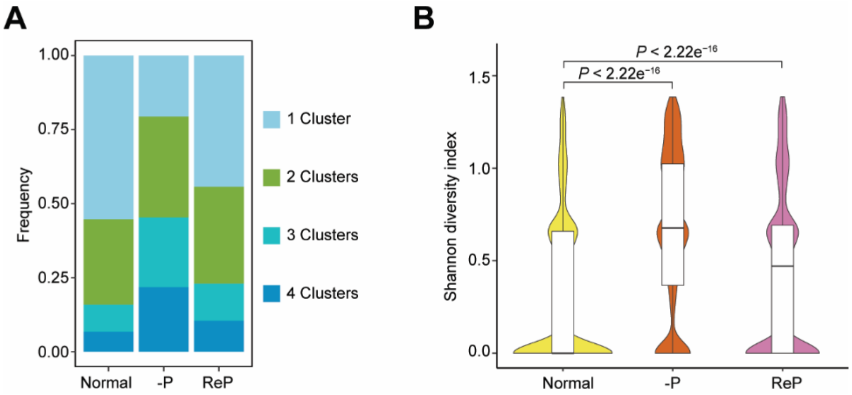

Supplemental Figure 3. DREEM analysis showing Pi starvation–induced global RSS heterogeneity in 100-nt RNA windows with more than 25,000-read coverage.

(A) DREEM analysis showing the increased prevalence of RNA structural heterogeneity in the transcriptome *in vivo*; –P induces RSS diversity compared with normal and ReP conditions. The number of clusters represents the total number of alternative RNA structures of a 100-nt RNA window *in vivo*.

(B) Violin plots showing the increased Shannon diversity index in –P compared with normal and ReP conditions. The *P* values were calculated using a Wilcoxon test. The midlines and box edges indicate the medians and quartiles, respectively. The whiskers extend to the farthest data point within 1.5 times the IQR from the box edges.

–P, Pi deficiency; ReP, Pi-refeeding following Pi deficiency.

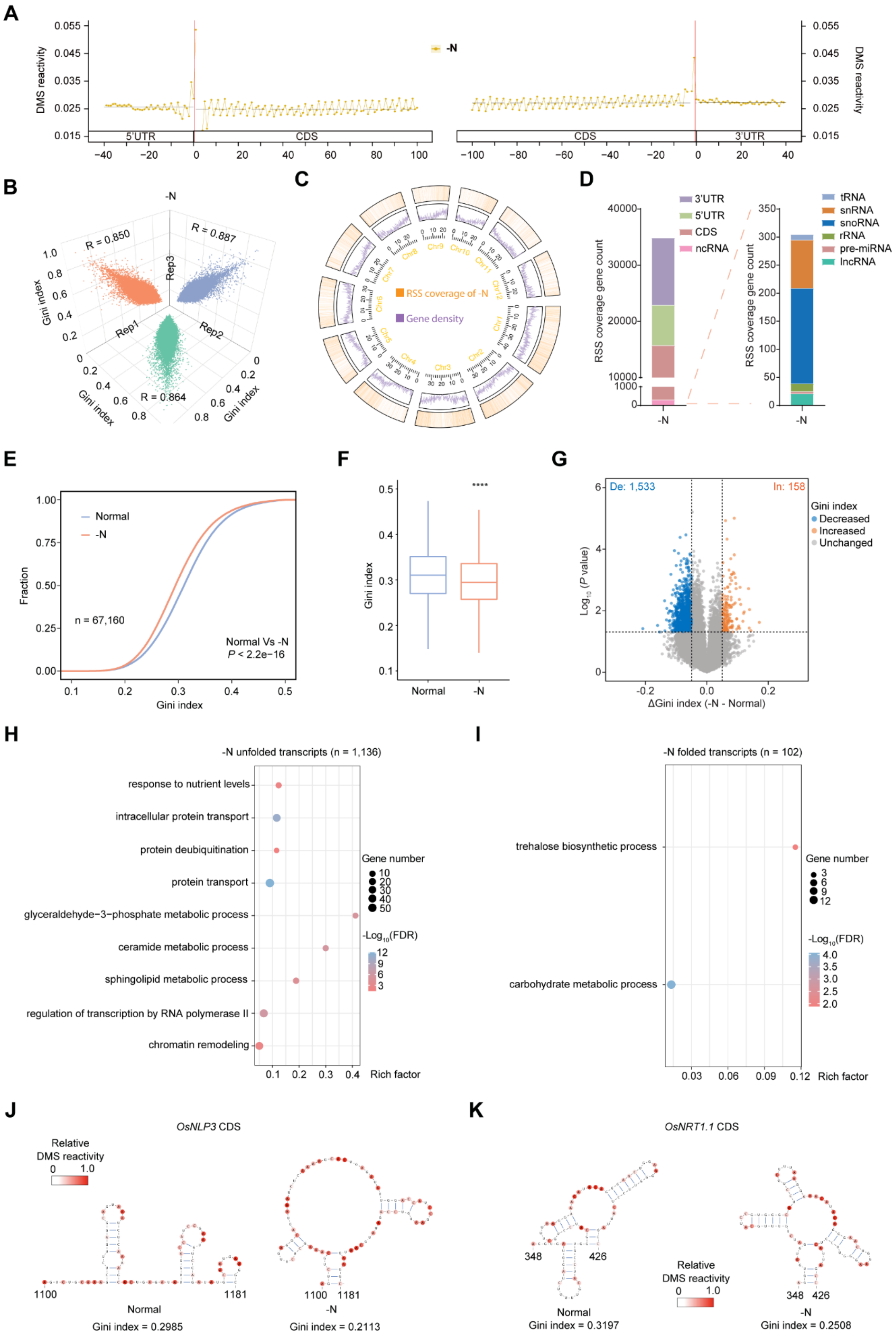

A
DMS reactivity
-N
5'UTR
CDS
CDS
3'UTR
B
-N
R = 0.850
R = 0.887
R = 0.864
Rep1
Rep2
Rep3
Gini index
C
RSS coverage of -N
Gene density
D
3'UTR
5'UTR
CDS
ncRNA
tRNA
snRNA
snoRNA
rRNA
pre-miRNA
lncRNA
RSS coverage gene count
E
Normal
-N
Fraction
n = 67,160
Normal Vs -N
P < 2.2e−16
Gini index
F
Gini index
****
Normal
-N
G
De: 1,533
In: 158
Gini index
Decreased
Increased
Unchanged
Log10 (P value)
ΔGini index (-N - Normal)
H
-N unfolded transcripts (n = 1,136)
response to nutrient levels
intracellular protein transport
protein deubiquitination
protein transport
glyceraldehyde−3−phosphate metabolic process
ceramide metabolic process
sphingolipid metabolic process
regulation of transcription by RNA polymerase II
chromatin remodeling
Gene number
-Log10(FDR)
Rich factor
I
-N folded transcripts (n = 102)
trehalose biosynthetic process
carbohydrate metabolic process
Gene number
-Log10(FDR)
Rich factor
J
OsNLP3 CDS
Relative DMS reactivity
1100
1181
Normal
Gini index = 0.2985
1100 1181
-N
Gini index = 0.2113
K
OsNRT1.1 CDS
348
426
Normal
Gini index = 0.3197
Relative DMS reactivity
348 426
-N
Gini index = 0.2508

Supplemental Figure 4. –N induces global changes in RSS.

(A) Metaplots showing DMS reactivities along different segments of mRNAs under –N conditions. mRNAs were aligned by their start/stop codons (vertical red lines). Yellow ribbon indicates the standard error from three biological repeats for –N samples.

(B) 3D scatterplots showing the high reproducibility of Gini index values among three biological repeats in –N samples. *R* is the Pearson correlation coefficient.

(C) Circos plot of the gene density and high-confidence RSS information coverage under N conditions, showing the high-coverage of high-confidence RSS information genome-wide.

(D) Bar diagram showing high-confidence RSS regions located at various types of RNA in –N samples, mainly at CDS regions, 3′ UTRs, and 5′ UTRs of mRNAs.

(E and F) Cumulative curves **(E)** and box plots **(F)** showing the moderate global decrease in Gini index in –N compared to normal conditions. The *P* values were calculated using a Kolmogorov-Smirnov test in **E** and a Wilcoxon test in **F**, respectively. The midlines and box edges indicate the medians and quartiles, respectively. The whiskers extend to the farthest data point within 1.5 times the IQR from the box edges. ****, $P < 0.0001$.

(G) Volcano plot of altered Gini indexes of high-conf. RSS windows under –N compared with normal conditions. De, RSS windows with significantly decreased Gini indexes; In, RSS windows with significantly increased Gini indexes. The numbers of corresponding altered RSS windows are shown.

(H and I) GO analysis of –N unfolded **(H)** and folded **(I)** transcripts.

(J and K) Modeling RSS of *OsNLP3* **(J)** and *OsNRT1.1* **(K)** based on DMS-MaPseq data showing the unfolded RNA structure under –N compared with normal conditions.

–N, nitrogen deficiency.

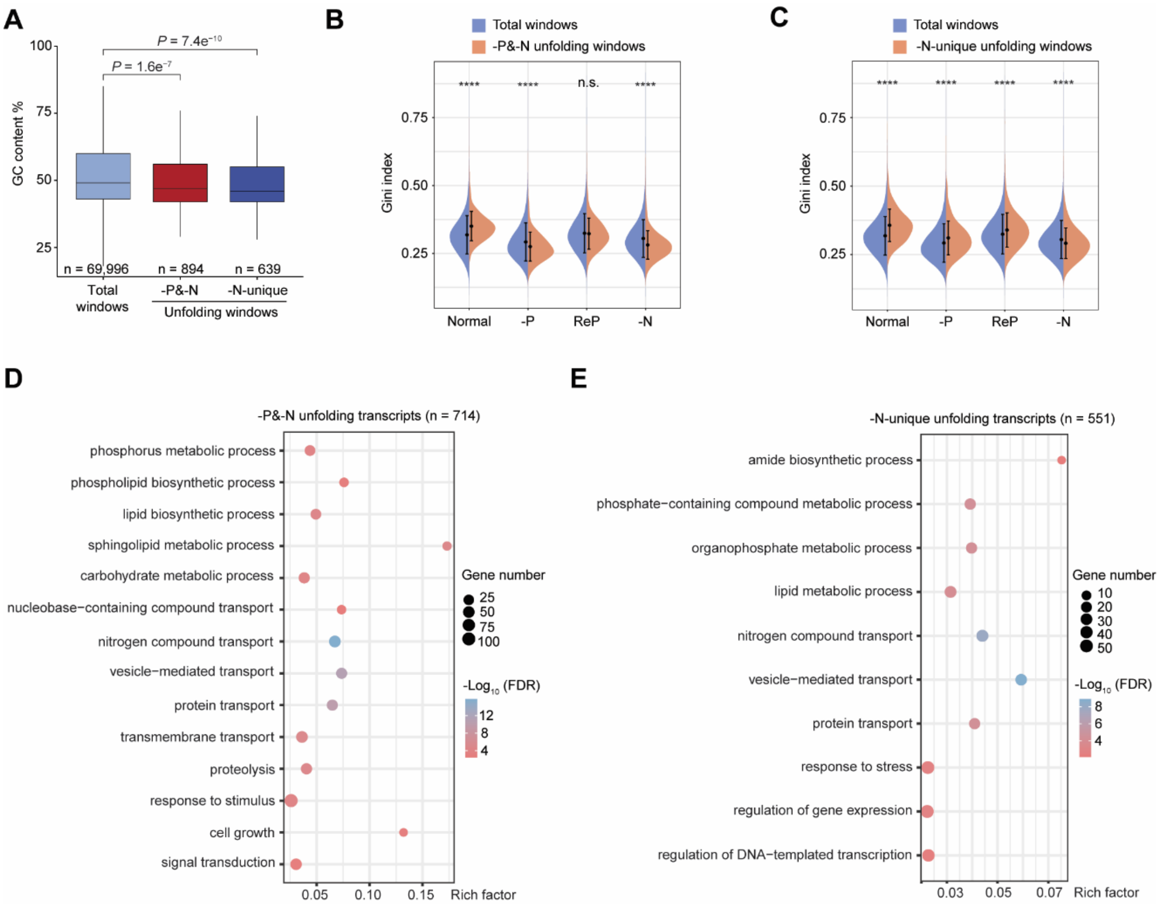

Supplemental Figure 5. The features of –N induced RSS-unfolding regions.

(A) Box plots showing significantly lower GC contents in both –P&–N and –N-unique RSS-unfolding windows than in total RSS windows. The midlines and box edges indicate the medians and quartiles, respectively. The whiskers extend to the farthest data point within 1.5 times the IQR from the box edges.

(B and C) Violin plots showing that the global Gini index of –P&–N (**B**) and –N-unique (**C**) RSS-unfolding windows under different nutrient conditions.

(D and E) GO analysis of –P&–N (**D**) and –N-unique (**E**) RSS-unfolding transcripts.

In (**A-C**), the *P* values were calculated using a Wilcoxon test. ****, $P < 0.0001$; n.s., no significant.

–P, Pi deficiency; ReP, Pi-refeeding following Pi deficiency; –N, nitrogen deficiency.

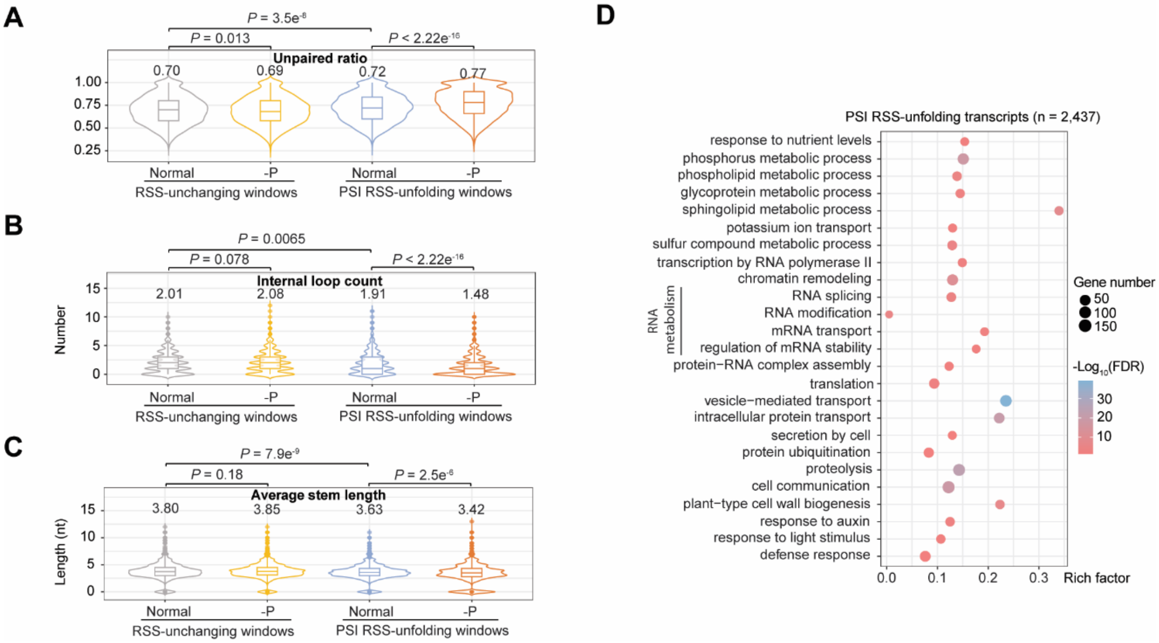

Supplemental Figure 6. RSS features and GO analysis of PSI RSS-unfolding regions.

(A-C) Diagrams showing the unpaired ratio (**A**), internal loop number (**B**), and average stem length (**C**) of RSS-unchanging windows (n = 3,543) and PSI RSS-unfolding windows (n = 3,838) under normal and –P conditions. The corresponding average values are shown. The midlines and box edges indicate the medians and quartiles, respectively. The whiskers extend to the farthest data point within 1.5 times the interquartile range (IQR) from the box edges. The *P* values were calculated using a Wilcoxon test. The average values are shown.

(D) GO analysis of PSI RSS-unfolding transcripts.

–P, Pi deficiency; PSI, Pi starvation induced; RSS, RNA secondary structure.

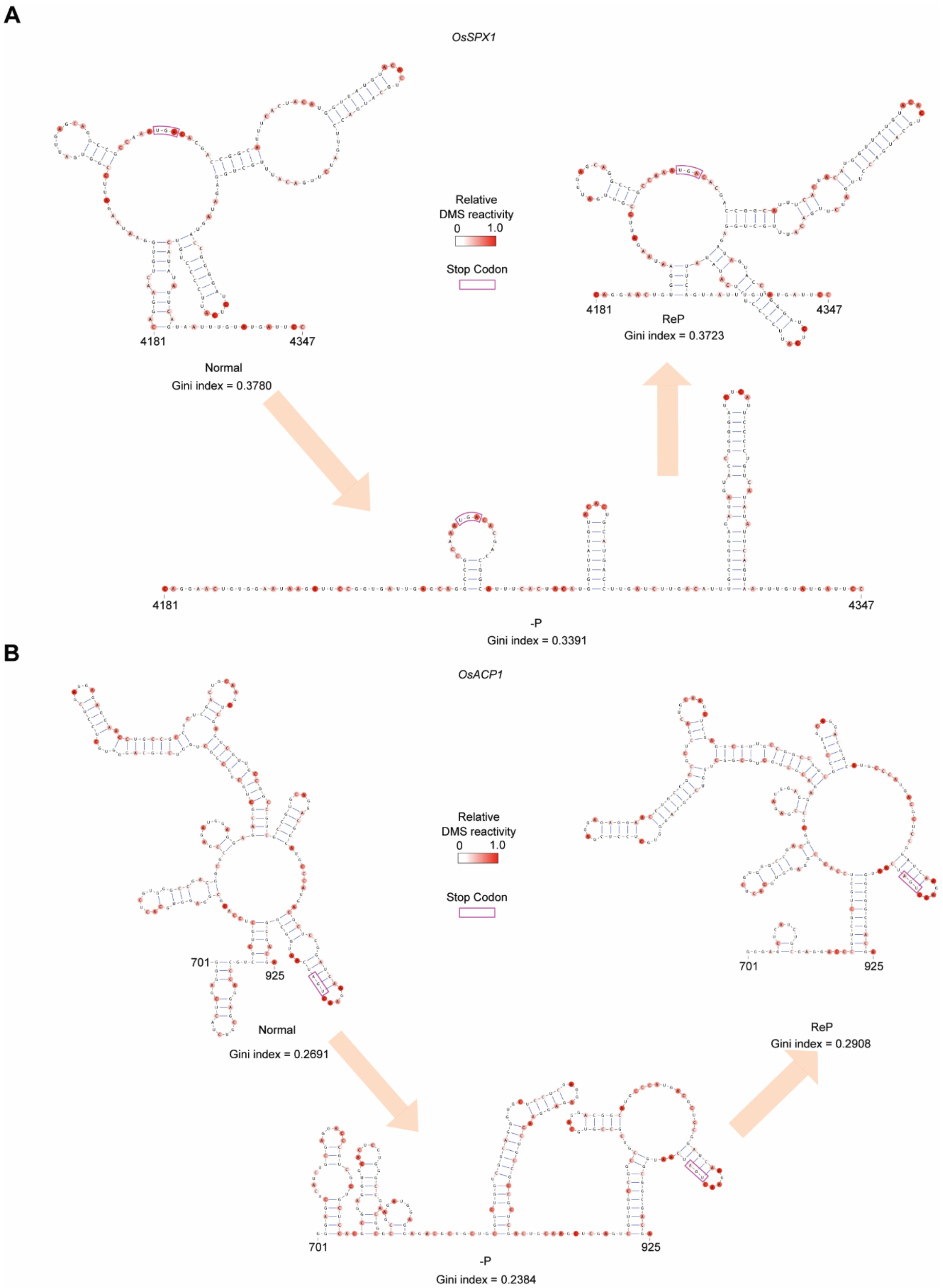

Supplemental Figure 7. The RSS of PSR transcripts is unfolded in response to –P.

(A and B) Modeling RSS of *OsSPX1* **(A)** and *OsACP1* **(B)** transcripts based on DMS-MaPseq data showing the unfolded RNA structures under –P compared with normal and ReP conditions. –P, Pi deficiency; ReP, Pi-refeeding following Pi deficiency.

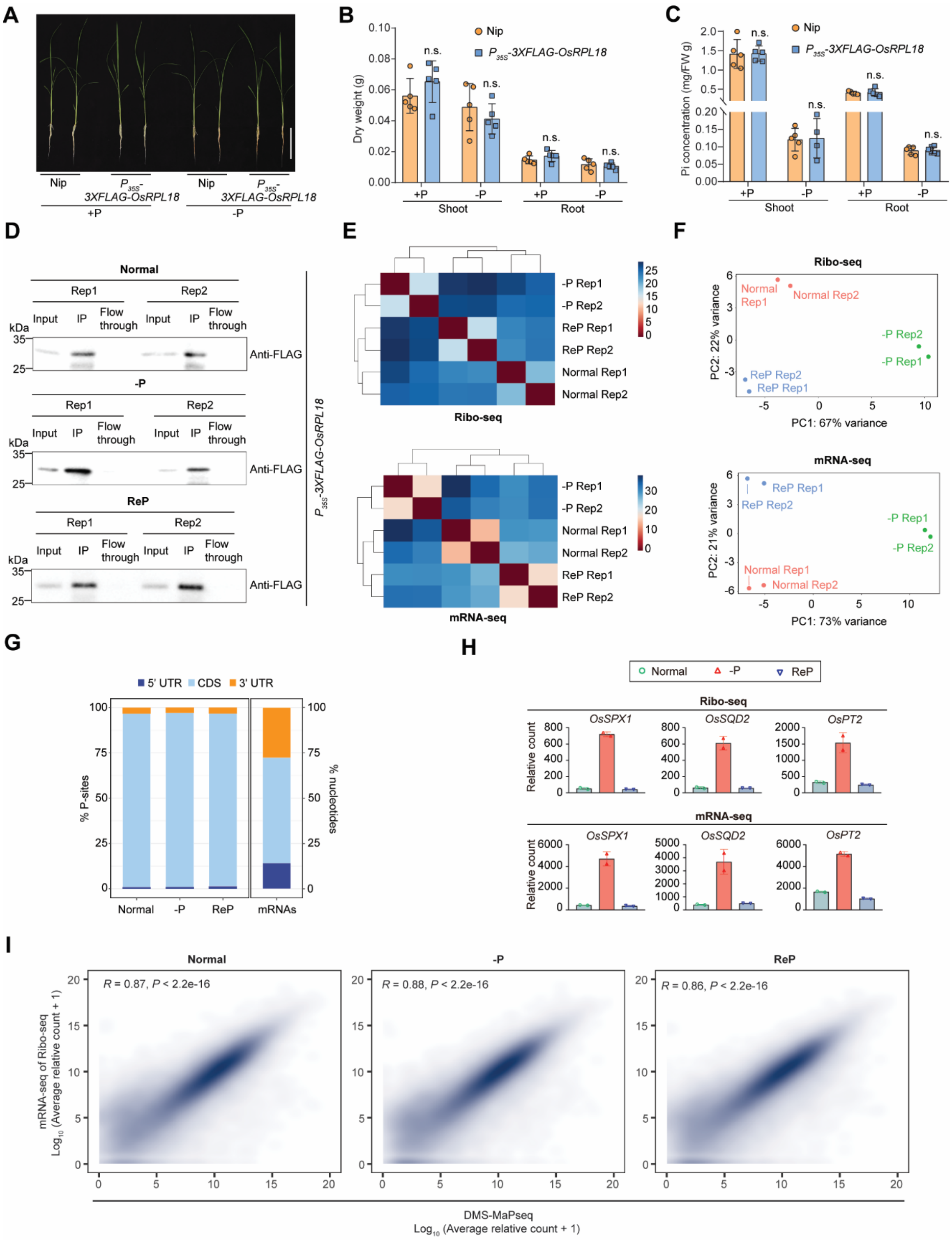

Supplemental Figure 8. High quality of the Ribo-seq data.

(A) Image showing no obvious difference in growth between Nip and *P*$_{35S}$*-3XFLAG-OsRPL18* under both normal (+P) and –P conditions. +P, normal condition. Bar = 10 cm.

(B and C) Bar plots showing no significant differences in either dry weight **(B)** or Pi concentration **(C)** in shoots and roots between Nip and P_{35S}*-3XFLAG-OsRPL18* under both normal (+P) and –P conditions. The *P* values were calculated using an unpaired two-tailed Student's *t*-test. n.s., no significant. Error bars with standard deviation were calculated from three biological replicates. +P, normal condition.

(D) Immunoblots showing highly efficient affinity purification of polysome–RNA complexes. IP, immunoprecipitate.

(E and F) Clustering **(E)** and PCA **(F)** showing high reproducibility among two biological replicates of TRAP-seq samples.

(G) Percentages of 5′ ends of 28-nt RPFs mapped at the 5′ UTR, CDS, and 3′ UTR in Ribo-seq data under normal, –P, and ReP conditions. The percentages of 5′ UTR, CDS, and 3′ UTR length in total mRNAs served as a control.

(H) Expression of the PSI genes *OsSPX1*, *OsSQD2*, and *OsPT2* based on Ribo-seq and mRNA-seq data for P_{35S}*-3XFLAG-OsRPL18* under normal, –P, and ReP conditions.

(I) Dot diagrams showing a high correlation of transcriptome data between mRNA-seq of Ribo-seq and DMS-MaPseq under normal, –P, and ReP conditions. *R* is the Pearson correlation coefficient. *P* values were calculated using the Pearson correlation test.

–P, Pi deficiency; ReP, Pi-refeeding following Pi deficiency.

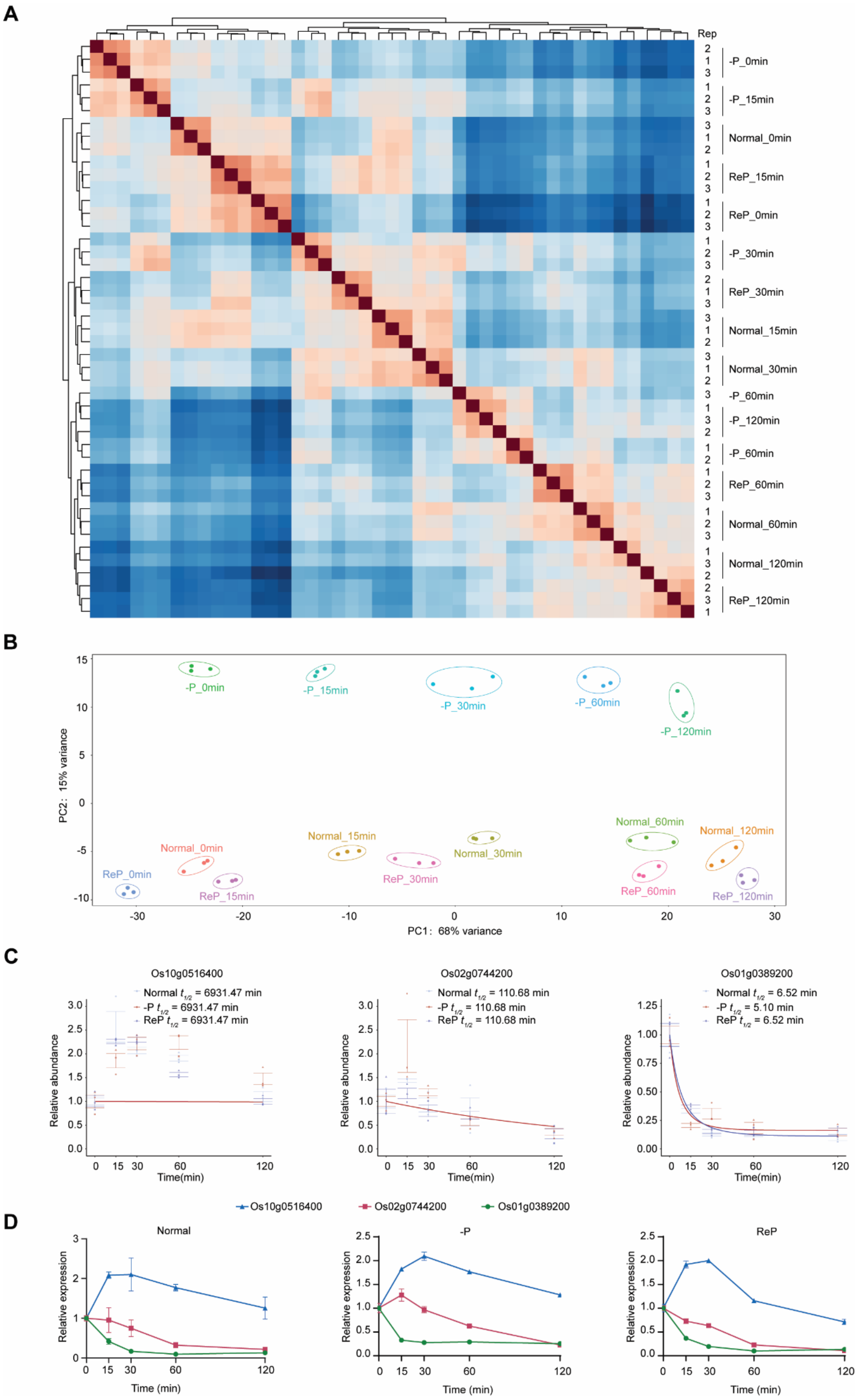
A
Rep
-P_0min
-P_15min
Normal_0min
ReP_15min
ReP_0min
-P_30min
ReP_30min
Normal_15min
Normal_30min
-P_60min
-P_120min
-P_60min
ReP_60min
Normal_60min
Normal_120min
ReP_120min
B
PC2: 15% variance
PC1: 68% variance
-P_0min
-P_15min
-P_30min
-P_60min
-P_120min
Normal_0min
Normal_15min
Normal_30min
Normal_60min
Normal_120min
ReP_0min
ReP_15min
ReP_30min
ReP_60min
ReP_120min
C
Os10g0516400
Normal $t_{1/2}$ = 6931.47 min
-P $t_{1/2}$ = 6931.47 min
ReP $t_{1/2}$ = 6931.47 min
Os02g0744200
Normal $t_{1/2}$ = 110.68 min
-P $t_{1/2}$ = 110.68 min
ReP $t_{1/2}$ = 110.68 min
Os01g0389200
Normal $t_{1/2}$ = 6.52 min
-P $t_{1/2}$ = 5.10 min
ReP $t_{1/2}$ = 6.52 min
Relative abundance
Time(min)
D
Os10g0516400
Os02g0744200
Os01g0389200
Normal
-P
ReP
Relative expression
Time (min)

Supplemental Figure 9. High quality and fidelity of the RNA decay data.

(A and B) Clustering (**A**) and PCA (**B**) showing high reproducibility among three biological replicates of the RNA decay samples.

(C) Profile of the decay of selected transcripts with various half-lives under normal, –P, and ReP conditions. Relative RNA abundances following the inhibition of transcription are shown, with bars indicating the means ± standard error of the mean, n = 3; thick lines indicate modeled values. The half-life ($t_{1/2}$) is indicated for each treatment.

(D) Target-specific RNA decay assay confirming the half-lives of the selected transcripts, reflecting the high fidelity of our transcriptome-wide RNA decay assay.

–P, Pi deficiency; ReP, Pi-refeeding following Pi deficiency.

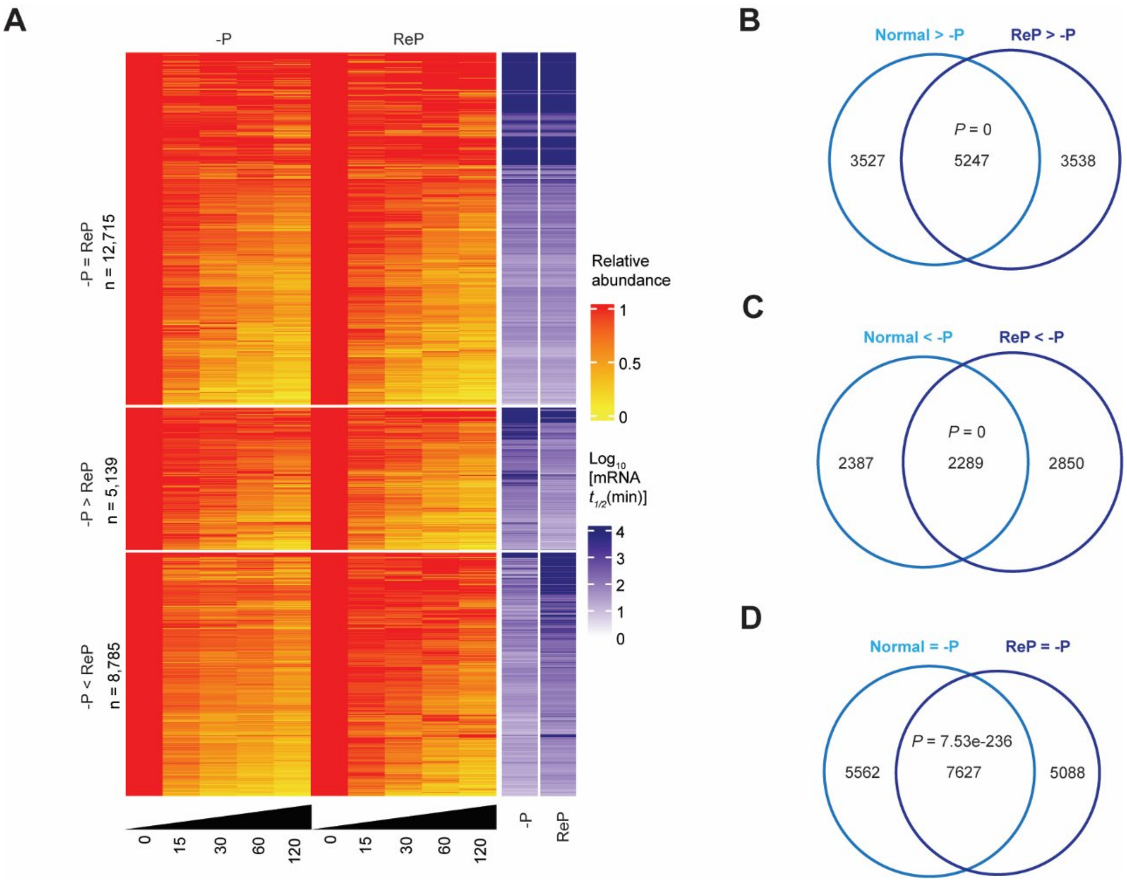

Supplemental Figure 10. Pi starvation induces a global decrease in RNA stability.

(A) Heatmap of RNA decay over 120 min in Nip under –P and ReP conditions. RNA decay dynamics were quantified by tracking the change in mean relative RNA abundance over time, with decay rates expressed as RNA half-life ($t_{1/2}$ in minutes).

(B-D) Venn diagrams showing the significantly overlapped transcripts with shortened (**B**), prolonged (**C**), and unchanged (**D**) half-lives between the –P vs Normal and –P vs ReP comparisons. *P* values were calculated with hypergeometric test.

–P, Pi deficiency; ReP, Pi-refeeding following Pi deficiency.

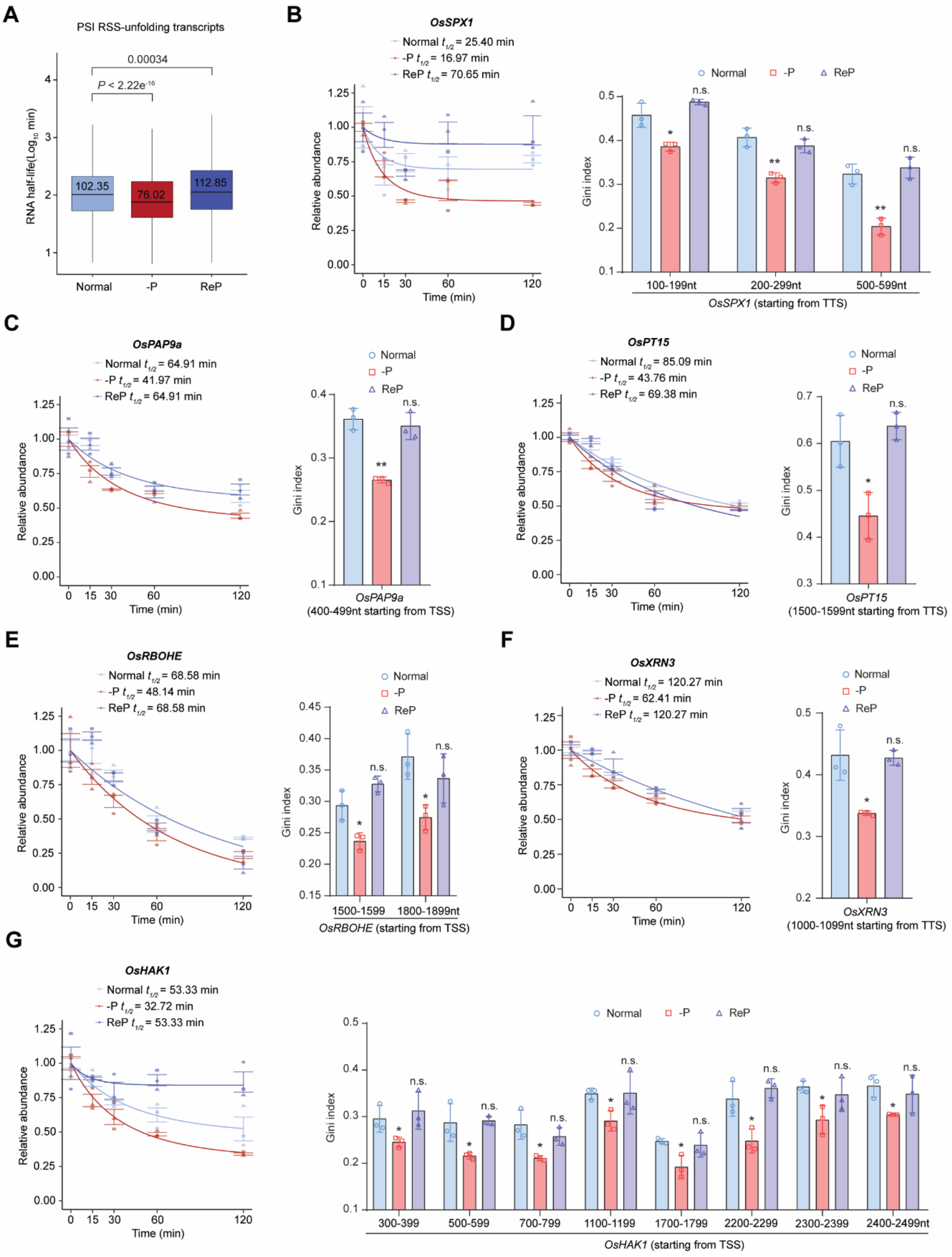

Supplemental Figure 11. Examples of transcripts with lower RNA stability and RSS Gini indexes under –P in roots.

(A) Box plots showing a global decrease in the half-lives of –P RSS-unfolded transcripts under –P, but not ReP, compared to normal conditions. The *P* values were calculated using a Wilcoxon test. The midlines and box edges indicate the medians and quartiles, respectively. The whiskers extend to the farthest data point within 1.5 times the IQR from the box edges.

(B-G) Both the half-lives and Gini indexes of *OsSPX1* **(B)**, *OsPAP9a* **(C)**, *OsPT15* **(D)**, *OsRBOHE* **(E)**, *OsXRN3* **(F)**, and *OsHAK1* **(G)** decreased in response to –P and were rescued after ReP. In the half-life profile, relative RNA abundances following the inhibition of transcription are shown, with bars indicating the means ± standard error of the mean, $n = 3$; thick lines indicate modeled values. The half-life ($t_{1/2}$) is indicated for each treatment. In the Gini index bar plots, the *P* values were calculated using an unpaired two-tailed Student's *t*-test. **, $P < 0.01$; *, $P < 0.05$; n.s., no significant.

–P, Pi deficiency; ReP, Pi-refeeding following Pi deficiency.

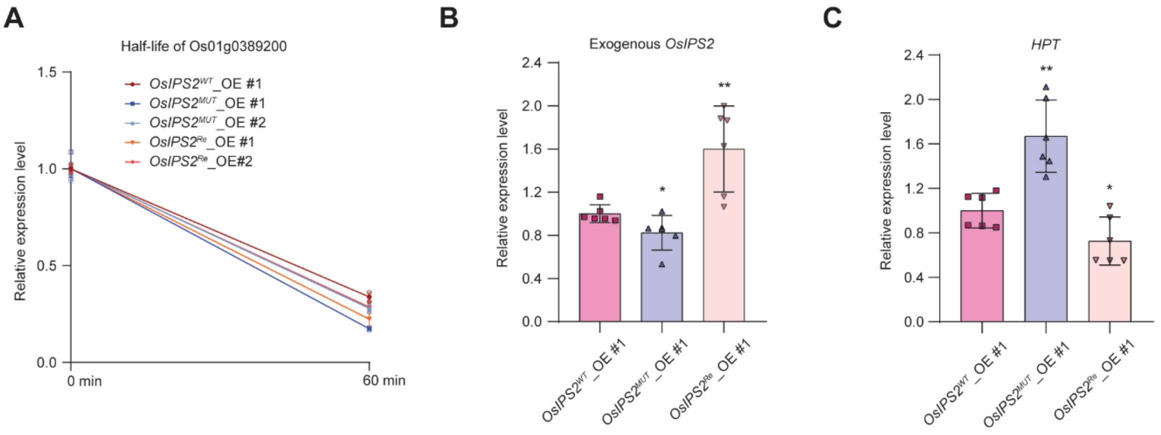

Supplemental Figure 12. The transcription was successfully stopped by chemical treatment and the expression levels of exogenous *OsIPS2* and *HPT* transcripts in Nip and transgenic plants.

(A) Target-specific RNA decay of the half-lives of control unstable transcripts of Os01g0389200 in the *OsIPS2^{WT}*_OE, *OsIPS2^{MUT}*_OE, and *OsIPS2^{Re}*_OE transgenic lines. The relative expression levels of the transcripts were normalized to those at the 0-min time point, where the ratio was arbitrarily set to 1, with standard deviation calculated from three biological repeats.

(B and C) Bar plots showing the expression levels of exogenous *OsIPS2* (**B**) and *HPT* transcripts (**C**) in roots of Nip, *OsIPS2^{WT}*_OE #1, *OsIPS2^{MUT}*_OE #1 and *OsIPS2^{Re}*_OE #1 plants. The relative expression levels of genes were normalized to those of *OsIPS2^{WT}*_OE #1, where the ratio was arbitrarily set to 1 with SD calculated from two biological repeats with three technical repeats. *$P < 0.05$; **$P < 0.01$, as determined using an unpaired two-tailed Student's *t*-test.

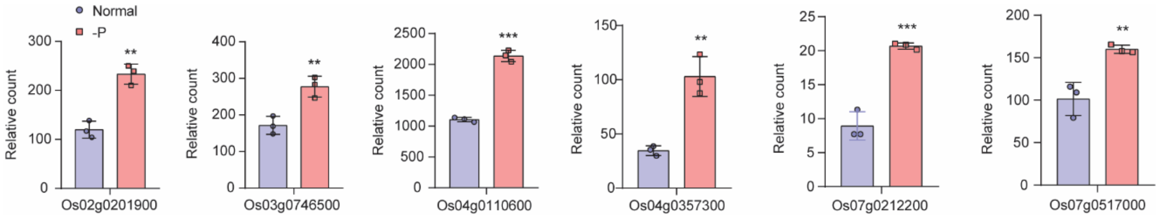

Supplemental Figure 13. Several RNA helicases and RBP genes are induced by –P.

The expression levels of Pi starvation induced RNA helicases and RNA binding protein-encoding genes under normal and –P conditions. ** $P < 0.01$ *** $P < 0.001$; unpaired two-tailed Student's *t*-test. –P, Pi deficiency.
